# Changing trends in anabolic‐androgenic steroid use within Scottish prisons: Detection, prevalence, and quantitation

**DOI:** 10.1002/dta.3790

**Published:** 2024-08-20

**Authors:** Caitlyn Norman, Richard L. Harries, Robert Reid, Lorna A. Nisbet, Niamh Nic Daéid

**Affiliations:** ^1^ Leverhulme Research Centre for Forensic Science, School of Science and Engineering University of Dundee Dundee UK; ^2^ Division of Clinical Chemistry and Pharmacology, Department of Biomedical and Clinical Sciences, Faculty of Medicine and Health Sciences Linköping University Linköping Sweden

**Keywords:** anabolic‐androgenic steroids, image performance enhancing drugs, polydrug use, prisons, tablet markings

## Abstract

Anabolic‐androgenic steroids (AASs) are a subclassification of image performance enhancing drugs (IPEDs). While AAS use is most prevalent among people in athletics, there is also high lifetime prevalence of AAS use among prisoners. This study reports the qualitative detection of AASs in seized samples from the Scottish prisons from 2019–2023. Additionally, methods were developed for the quantitative analysis of AASs using gas chromatography–mass spectrometry (GC–MS) and applied to 61 samples of tablets or powders seized from Scottish prisons between July 2022 and July 2023. Since 2022, there has been an increase in AAS detections in the Scottish prisons. Oxymetholone was the most prevalent AAS, followed by metandienone (methandrostenolone, methandienone), methyltestosterone, oxandrolone, mestanolone (methylandrostanolone), stanozolol, and androstenedione. Multiple AASs were found in 21 samples and 10 samples contained other drugs, including amitriptyline, sertraline, zopiclone, mirtazapine, sildenafil, etizolam, Δ^9^‐tetrahydrocannabinol, and the synthetic cannabinoid MDMB‐INACA. Most AAS samples were tablets (77.0%), although they were also detected in powders, herbal material, e‐cigarettes, and a fragmented soap bar‐type sample. There was a large variation in the concentration of AASs in the tablets and powders seized from the Scottish prisons, demonstrating AASs are another highly variable component of the polydrug use situation in prisons, the effects of which need to be examined further.

## Graphical abstract

This study reports the qualitative and quantitative detection of anabolic‐androgenic steroids (AASs) in seized samples from prisons. Since 2022 there has been an increase in AAS detections with oxymetholone the most prevalent AAS, followed by metandienone, methyltestosterone, and oxandrolone. 34% of samples contained multiple AASs and 16% contained another drug. There was a large variation in the concentration of AASs in the tablets and powders, demonstrating AASs are another highly variable component of the polydrug use situation in prisons.

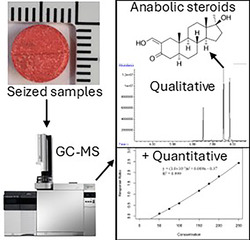

## 1 INTRODUCTION

Anabolic‐androgenic steroids (AASs) are a subclassification of image performance enhancing drugs (IPEDs) synthetically derived from testosterone. They have both muscle building (anabolic) and masculinizing (androgenic) properties, which can lead to an observed increase in fat‐free mass, muscle size, and strength, especially when combined with strength exercise.^1^, ^2^ AAS is often misused to enhance performance, increase strength, and improve physical appearance but also to enhance libido and sense of well‐being.^3^, ^4^

While AAS use is most prevalent among frequent gym goers and athletes, people not involved in any sports activity also use AASs with a reported lifetime prevalence of 0.4–6.7%.^3^ Higher lifetime prevalence of AAS use among illicit substance users, patients in substance use disorder treatment, and prisoners have also been reported.^5^, ^6^ Based on a meta‐analysis of relevant literature, the lifetime prevalence of AAS use among prisoners and arrestees was found to be about 12.4% (average from 6 studies).^7^ In the United Kingdom, the 2016 image and performance enhancing drugs national survey found that 17% (*n* = 112 of 684) of people using IPEDs in the previous 12 months reported prior incarceration in a young offenders institute or prison. Of those, 29% (*n* = 33 of 112) reported having consumed IPEDs during incarceration.^8^ This is in line with the results from four Scottish prisoner surveys conducted from 2011–2017 that found 22–36% of prisoners who reported injecting drugs in prison in the 4 weeks prior to the survey (1–2% of respondents) reported injecting AASs. Unfortunately, prisoners were not asked about their use of non‐injectable AASs.^9^, ^10^, ^11^, ^12^ AAS use during incarceration has also been analytically confirmed with 8.5% of urine samples (*n* = 155 of 1833) collected in May and June 2015 from 31 South East and North West England prisons testing positive for an AAS.^13^

The reasons for AAS use by prisoners remain unclear, but it has not been found to be associated with sports activities or exercise.^5^, ^14^ Instead AAS use has been found to be associated with criminality,^4^, ^6^ psychiatric disorders,^15^ and use of illicit drugs or diverted prescriptions,^4^, ^5^, ^16^ all of which are more prevalent among prisoners than the general population.^13^, ^17^, ^18^ This suggests that it is possible the higher rates of AAS use by prisoners may be an artifact of these three factors. This is supported by the reported low rates of AAS use during incarceration in comparison to that of other drugs,^19^, ^20^, ^21^ indicating the reasons for AAS use by prisoners are unlikely to be associated with imprisonment.

On the other hand, AASs have been observed to have become part of the drug arsenal for polydrug users,^4^ where drugs are often combined in order to counteract adverse side effects or enhance the desired effects of different drugs. Some of the drugs taken concurrently with AASs in order to counteract negative side effects include anti‐depressants for depression^22^, ^23^; cannabis, z‐drugs, or benzodiazepines for poor sleep quality and insomnia^24^, ^25^; anti‐psychotics for psychosis^22^; PDE‐5 inhibitors for sexual dysfunction^26^, ^27^, ^28^, ^29^; and antiestrogens for the formation of breast tissue (gynecomastia).^30^ In addition, stimulants such as amphetamines and cocaine have been found to increase fat metabolism, thereby increasing the desired effects of the AASs.^31^ AASs have also been reported to be used by people in substance use disorder treatment in order to counteract the reduction in weight and muscle mass caused by long‐term substance use.^5^

There is little information available in the literature about the prevalence of different AASs used recreationally, but testosterone esters frequently feature as the most prevalent AAS among the general population.^32^ In addition, within 10 North West English prisons, 151 of 1088 seizures (13.7%) contained AASs, with the most prevalent detections for testosterone esters, oxymetholone, stanozolol, oxandrolone, and trenbolone. In comparison, within 222 AAS positive prisoner urine samples from 31 English prisons, nandrolone and testosterone were the most frequently detected, followed by dehydroepiandrosterone, stanozolol, trenbolone, and oxymetholone. Differing analysis methodologies was provided as reasoning for the variation in the seized sample and urine AAS findings.^13^

This study reports the qualitative detection of AASs within Scottish prisons from 2019 to 2023. Additionally, methods for the quantitation of six different AASs detected over the study period, mestanolone (methylandrostanolone), metandienone (methandrostenolone, methandienone), methyltestosterone, oxandrolone, oxymetholone, and stanozolol, within tablets and powders were developed and applied to seized prison samples.

## 2 MATERIALS AND METHODS

### 2.1 Materials

HPLC grade (purity ≥ 99.9%) methanol and acetonitrile were supplied by Fisher Chemicals (Loughborough, UK). Bupivacaine hydrochloride monohydrate (internal standard) was supplied by Sigma Aldrich (Poole, UK).

#### 2.1.1 Reference standards

Mestanolone (98.8% purity) and metandienone (97.5% purity) were obtained from Tokyo Chemical Industry (TCI) Co Ltd (Tokyo, Japan), 17α‐methyltestosterone (99% purity) from Sigma‐Aldrich (Poole, UK), stanozolol (99.52% purity) from LGC Limited (Teddington, UK), oxymetholone (98% purity) and etizolam (99.4% purity) from Chiron AS (Trondheim, Norway), and oxandrolone (purity ≥ 98%) and MDMB‐INACA (purity ≥ 98%) from Cayman Chemical (Ann Arbor, MI, USA).

#### 2.1.2 Seized samples

Samples included in this study were non‐attributable samples seized by the Scottish Prison Service (SPS) between January 30, 2019, and July 8, 2023. Samples may have been recovered during personal or cell searches or following detection of a drug during the screening of incoming items (mostly mail) by prison staff using ion mobility spectrometry (IMS) instruments.

### 2.2 Methods

#### 2.2.1 Qualitative analysis

The method adopted for the examination, extraction, and analysis of powder, tablet, and herbal material samples has been described previously within studies focusing on detections of SCRAs and novel benzodiazepines.^33^, ^34^ In brief, for powder samples, 10 mg of the material was dissolved in 1 mL of 0.25 mg/mL bupivacaine in methanol and vortexed and/or centrifuged (1 min). For tablet samples, one tablet or tablet fragment was crushed using a mortar and pestle, and the resulting powder was processed as previously described for powder samples. In the event that there were multiple tablets with similar visual characteristics in a seizure, one tablet was randomly selected for analysis. For herbal material samples, 10 mg of the material was extracted in 0.5 mL of 0.25 mg/mL bupivacaine in methanol by ultrasonication (5 min). The supernatant was then transferred to a gas chromatography–mass spectrometry (GC–MS) vial for analysis. For sealed e‐cigarette cartridges, as described previously,^35^ 1 mL of 0.25 mg/mL bupivacaine in methanol was pipetted down the mouthpiece into a beaker and then the entire e‐cigarette cartridge was sonicated (5 min) in the solution. The extract was then transferred to a GC–MS vial for analysis.

#### 2.2.2 Quantitative analysis

Based on the results of the qualitative analysis, quantitative methods for the analysis of mestanolone, metandienone, methyltestosterone, oxandrolone, oxymetholone, and stanozolol were developed.

##### Preparation of calibration and check standards

Individual stock solutions (1 mg/mL) of mestanolone, metandienone, methyltestosterone, oxandrolone, and stanozolol were prepared by dissolving 5 mg of certified reference material into 5‐mL methanol. A 1 mg/mL stock solution of oxymetholone was produced in a similar manner using acetonitrile instead of methanol. Oxymetholone has been shown to degrade to mestanolone and a methanol adduct in methanol and ethanol, likely at the injection port, hence why it was necessary to prepare this solution in acetonitrile.^36^, ^37^

A 7‐point calibration range was prepared for each AAS from the 1 mg/mL stock solutions, 0.125 μg/mL bupivacaine in methanol (acetonitrile for oxymetholone), and methanol (acetonitrile for oxymetholone). Some AASs had slightly different limits of quantitation (LOQs); however, the calibration standards used were kept as similar to each other as possible. A calibration range of 25, 50, 75, 100, 150, 200, and 250 μg/mL was used for mestanolone, metandienone, methyltestosterone, and oxandrolone; 50, 75, 100, 150, 175, 200, and 250 μg/mL were used for oxymetholone; and 50, 75, 100, 150, 200, 250, and 300 μg/mL were used for stanozolol.

For method validation, three check standards were prepared by an independent analyst at 40, 125, and 225 μg/mL for mestanolone, metandienone, methyltestosterone, and oxandrolone; 60, 125, and 225 μg/mL for oxymetholone; and 60, 175, and 275 μg/mL for stanozolol. For the sample runs, the two lowest check standards as listed above were prepared by an independent analyst. The stock solution, calibration standards, and check standards were stored in a freezer (−20°C) until use. Bupivacaine with a final concentration of 62.5 μg/mL was used as the internal standard for all calibration and check standards.

##### Sample extraction

Although AASs were detected in e‐cigarette capsules^35^ and herbal material, there was not enough sample available for extraction efficiency or quantitation to be undertaken. Therefore, only powder and tablet samples were quantitatively analyzed; 10 mg each of the homogenized samples was weighed into a 1.5‐mL Eppendorf tube. The sample was sequentially extracted by centrifugation (1 min) in 0.5‐mL methanol. Five sequential extractions achieved 100% extraction efficiency for mestanolone, metandienone, methyltestosterone, and stanozolol and >99% for oxandrolone, but six sequential extractions in acetonitrile were required to achieve >99% extraction efficiency for oxymetholone. The full extraction efficiency data can be found in the Supporting Information (Section S1).

The extracts were combined and evaporated to dryness under nitrogen at room temperature. Each sample was then reconstituted in 0.5 mL 62.5 μg/mL bupivacaine in methanol (acetonitrile for oxymetholone) and vortex mixed for 30 s. Samples were then diluted as necessary 1–40× with 62.5 μg/mL bupivacaine in methanol (acetonitrile for oxymetholone) until they fell inside the calibration range. Bupivacaine with a final concentration of 62.5 μg/mL was used as the internal standard for all samples.

### 2.3 Instrumental analysis

Analysis was performed using a 7820A gas chromatograph coupled to a 5977E mass spectrometer (Agilent Technologies, Santa Clara, CA, USA). Injection mode: 1‐μL sample injection was used, with a 15:1 split into a 4‐mm internal diameter deactivated glass liner pre‐packed with quartz wool, injection port temperature: 250°C, carrier gas: He, flow: 1 mL/min. Column: HP‐5MS UI, 30 m × 0.25 mm × 0.25 μm (Agilent Technologies). GC oven: 110°C held for 1 min; 30°C/min to 280°C, held for 11 min; total run time: 17.67 min; transfer line: 290°C. The mass spectrometer was operated in electron (EI) ionization mode. Ionization conditions: 70 eV in full scan mode (45–450 amu), ion source: 230°C, quadrupole: 150°C.

#### 2.3.1 Qualitative analysis

Compound identification required comparison of the compound retention times and mass spectra in seized samples to that of a reference standard of known origin analyzed within 24 h of the seized samples under the same instrumental conditions. For a positive identification, the GC–MS retention times from seized sample extracts had to fall within 0.05 min of the retention time of the reference standard or 0.1 min in cases of high concentration leading to distorted peak shape and shifted chromatographic peak apex. In addition, a reverse match (RMatch) factor from the SWGDRUG mass spectral library (version 3.12, released January 16, 2023), which measures the difference between the mass spectrum of the sample chromatographic peak to spectra held in the spectra library, was required to be greater than 850/1000 for positive identification, with no baseline subtraction.

Prior to June 2023, full analytical confirmation of AASs was not the principle focus of the Scottish Prisons Non‐Judicial Seizure Drug Monitoring Project. While most samples were able to be re‐sampled and analyzed to obtain complete compound identification, samples seized before May 2022 were not retained for further analysis, and some samples did not have sufficient sample remaining to allow re‐sampling. Therefore, these compound identifications were solely based on the SWGDRUG mass spectral library results and considered to be preliminary identifications. This is also the case for some of the drugs found in mixtures with AASs that were not the primary focus of the project.

#### 2.3.2 Quantitative analysis

For quantitative analysis, the GC–MS was operated the same as described above, except a 10:1 split was used, and the total run time was reduced to 10.67 min by reducing the hold time at 280°C to 4 min. For the quantitation of samples with stanozolol, a selected ion monitoring (SIM) method was used due to poor peak shape and baseline separation in the total ion chromatogram (TIC). The same GC–MS method was used as above, but for the MS method, the acquisition type was changed to SIM with two time segments. From 3.0 min, the MS scanned for the ion at *m*/*z* 140.00 for bupivacaine with dwell time for each ion of 100 ms. From 8.0 mins, the MS scanned for the ions at *m*/*z* 328.00 (quantitation) and 96.00 (qualifier) for stanozolol with dwell time for each ion of 100 ms. This methodology monitored a reduced number of ions because it was not being used for analyte identification as identification was previously performed based on a full mass spectra and reference standard comparison as described in Section 2.2.1.

For each analysis batch, the instrument was calibrated with a 7‐point calibration curve (25–250 μg/mL for mestanolone, metandienone, methyltestosterone, and oxandrolone; 50–250 μg/mL for oxymetholone; and 50–300 μg/mL for stanozolol). Two independently prepared check standards (40 and 125 μg/mL for mestanolone, metandienone, methyltestosterone, and oxandrolone; 60 and 125 μg/mL for oxymetholone; and 60 and 175 μg/mL for stanozolol) were injected at the beginning and end of each sequence. The maximum allowable bias for these check standards was ±15% based on recommendations by the United Nations Office on Drugs and Crime (UNODC).^38^

All GC peaks were integrated in the Agilent MassHunter Software. The resulting quantitative data were processed using an R script, which produced a quadratic calibration curve and solved for x. This allowed the script to produce an output that included the calculated peak area ratio and calculated concentration (μg/mL) for each sample. The R script is available in the Supporting Information.

#### 2.3.3 Method validation

Method validation of the quantitation of AASs in seized samples was performed according to the UNODC and Scientific Working Group for the Analysis of Seized Drugs (SWGDRUG) recommendations.^38^, ^39^ The following validation parameters were evaluated: limit of detection (LOD), LOQ, linearity, precision, and bias. The LOD was 10 μg/mL for oxandrolone; 20 μg/mL for mestanolone, metandienone, and methyltestosterone; and 50 μg/mL for oxymetholone and stanozolol. The LOQ was 25 μg/mL for all, except oxymetholone and stanozolol with a LOQ of 50 μg/mL. The bias was found to be 10% for mestanolone, 11% for metandienone, 7% for methyltestosterone, 8% for oxandrolone, 14% for oxymetholone, and 10% for stanozolol. Full validation data can be found in the Supporting Information.

## 3 RESULTS AND DISCUSSION

### 3.1 Qualitative results

**FIGURE 1:**
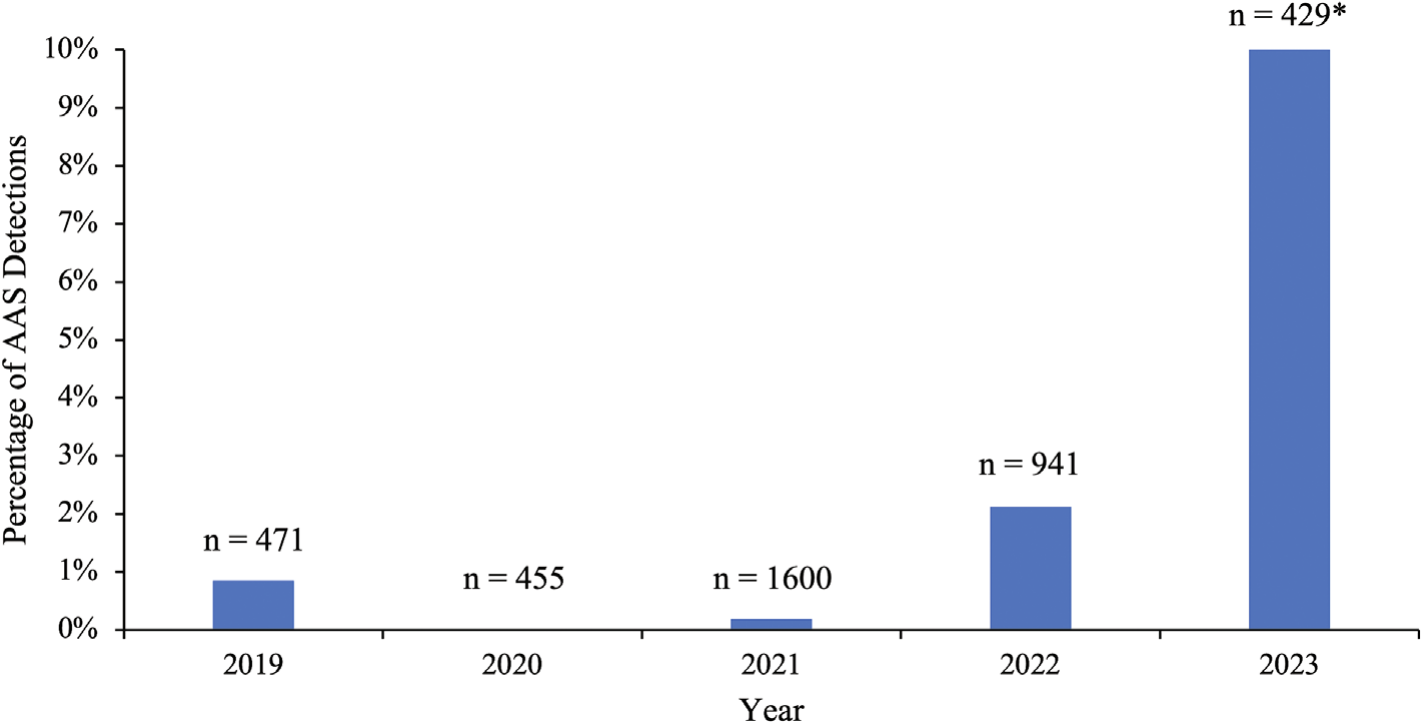
Percentage of samples seized from the Scottish prisons each year found positive for one or more anabolic‐androgenic steroids (AASs). *Data for 2023 is only through July 14, 2023.

Out of 3896 samples seized from Scottish prisons between January 2019 and August 2023 submitted for testing, 71 samples from 63 seizures were found to contain one or more AAS. AASs were rarely detected between 2019 and 2021, having been detected in only 0.85% (*n* = 4) of samples in 2019 and 0.19% (*n* = 3) of samples in 2021, with no AASs detected in 2020. However, as shown in Figure 1, in 2022, detections of AAS increased and became the third most prevalent drug class detected in the Scottish prisons in 2023 (10.26% [*n* = 44] of all samples), behind SCRAs and novel benzodiazepines.

As the AAS detections prior to May 2022 were not confirmed via comparison with reference standards and therefore considered preliminary, this study focuses on the qualitative results from July 2022 to July 2023 (*n* = 61 samples from 48 seizures obtained from 9 of 17 Scottish prisons). The complete qualitative results of the samples can be found in Table S8.1. Oxymetholone was the most prevalent AAS (*n* = 28), followed by metandienone (*n* = 17), methyltestosterone (*n* = 16), oxandrolone (*n* = 13), mestanolone (*n* = 4), stanozolol (*n* = 4), and androstenedione (*n* = 1; preliminary identification). It should be noted that mestanolone is a metabolite and degradation product of oxymetholone in methanol and ethanol.^36^, ^37^ All samples were initially extracted in methanol as part of the routine screening method used for all prison samples; however, upon discovering the degradation of oxymetholone, two of the samples found positive for mestanolone were re‐extracted in acetonitrile, in which oxymetholone is stable, suggesting that these were genuine detections of mestanolone and not a result of sample degradation. Unfortunately, there was not enough material remaining for a second extraction in acetonitrile of the other two mestanolone detections, one of which was found to also contain oxymetholone; therefore, mestanolone could be the result of degradation and cannot be confirmed as genuine detections.

Despite their reported prevalence in other studies,^13^, ^32^ testosterone esters and nandrolone were not detected in this study. This is likely because these AASs are predominantly found as aqueous or oil‐based injectables,^37^, ^40^ whereas the majority of samples in this study were tablets (see Section 3.1.2 for more information) and therefore contained oral AASs.^41^ Available data from user reports and limited analytical studies indicate, in correspondence with this study, that metandienone, oxandrolone, oxymetholone, and stanozolol are commonly used oral AASs.^42^, ^43^, ^44^, ^45^, ^46^, ^47^

#### 3.1.1 Mixtures

Multiple AASs were found in 34.4% of samples (*n* = 21), 10 of which contained methyltestosterone and metandienone; 2 contained methyltestosterone, metandienone, and oxandrolone; 2 contained oxymetholone and mestanolone; 2 contained oxymetholone and metandienone; 1 contained stanozolol and oxandrolone; 1 contained methyltestosterone and mestanolone; 1 contained methyltestosterone, oxymetholone, and androstenedione; 1 contained metandienone, oxymetholone, and stanozolol; and 1 contained stanozolol, oxymetholone, metandienone, and methyltestosterone. The combined use of multiple AAS, known as “stacking,” is a common practice among people who use AASs. Stacking has become increasingly prevalent, with samples seized in Sweden by the police and from prisoners found to contain increasing numbers of compounds. In 1999, urine samples typically contained one or two AASs, whereas in 2009, there were as many as eight different AASs identified.^48^ It has been suggested that stacking is used to exploit different androgen receptors to produce a synergistic effect.^49^

In addition to detecting mixtures of different AASs in individual samples, AASs were also detected with other drugs, most often prescription drugs, including the anti‐depressants amitriptyline (*n* = 2) and sertraline (*n* = 1), the sleeping pill (z‐drug) zopiclone (*n* = 2), the antipsychotic mirtazapine (*n* = 1), and the phosphodiesterase‐5 (PDE‐5) inhibitor sildenafil, also known as Viagra, (*n* = 1). In addition, in one case, an AAS tablet was detected in the same seizure as a tablet found to contain letrozole, an aromatase inhibitor and antiestrogen medication typically used to treat breast cancer. AASs were also detected alongside illicit drugs, including the novel benzodiazepine etizolam (*n* = 2), the SCRA MDMB‐INACA (*n* = 1), and Δ^9^‐tetrahydrocannabinol (THC; *n* = 1). In all these cases, the AAS was present at a low amount, where it always fell below the LOQ (see Section 3.2 for more information). This indicates the AAS may be present due to cross‐contamination, such as being stored or transported in the same vessel by prisoners. None of these samples were placed in the same seizure bag as another sample positive for AAS, reducing the possibility of cross‐contamination at the point of seizure. This is also supported by the tablet markings on some of these samples being the same as tablet markings previously found for the other drugs, as discussed in more detail below in Section 3.1.2.

The additional drugs detected alongside AASs in this study are in agreement with the drugs previously found to be commonly used concurrently with AASs as discussed in the introduction. Unfortunately, the effects of combining AASs with other drugs in a recreational context still require examination to understand the potential for additional harm from this polydrug use.

#### 3.1.2 Sample formats

Most AAS samples were tablets (*n* = 47, 77.0%), although they were also detected in powders (*n* = 7, 11.5%), herbal material (*n* = 1, 1.6%), and a fragmented soap bar‐type sample (*n* = 1, 1.6%). The remaining five samples (8.2%) were e‐cigarette cartridges, which were previously reported in Harries et al.^35^ Oral and intramuscular injection are the most common routes of administration for AASs by recreational users. In the 2015 Global Drug Survey, 35.6% of AAS administering participants (*n* = 1008) reported the sole use of injectable AASs, 35.8% reported sole use of oral AASs, and 28.5% reported the use of both during their lifetime.^50^ Given the limited availability of syringes and injecting equipment in prison, it is unsurprising that the majority of AASs in this study were found to be tablets and powders with oral ingestion the most likely route of administration. However, the detection of AASs in herbal material and vape pods indicates some possible experimentation with novel modes of AAS use, such as smoking or vaping.

**FIGURE 2:**
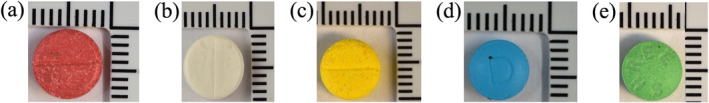
Examples of tablets seized from the Scottish prisons found positive for anabolic‐androgenic steroids (AASs): (a) FL23/0276‐1: 1× red tablet seized March 17, 2023, with oxymetholone and mestanolone; (b) FL22/0773: 1× white tablet seized July 18, 2022, with methyltestosterone and metandienone; (c) FL23/0203: 1× yellow tablet seized April 24, 2023, with oxandrolone; (d) FL23/0360: 1× blue tablet seized May 2, 2023, with oxymetholone and amitriptyline; and (e) FL23/0316: 1× green tablet seized May 15, 2023, with oxandrolone.

Examination photographs of some tablets are provided in Figure 2 as examples. Example photos of the remaining sample types can be found in Figure S9.1. The complete tablet markings data can be found in Table S10.1. Most tablets containing AAS were round (*n* = 39, 83.0%), but there were also oval (*n* = 3), hexagonal (*n* = 1), and egg (*n* = 1) shaped tablets. Three samples contained only tablet fragments, so the shapes of the tablets were unable to be determined. Three of the five non‐round tablets were found to contain oxymetholone mixed with another drug, including sertraline, zopiclone, and mirtazapine, indicating the change in shape may be in relation to the presence of the other drug, rather than the AAS. White was the most prevalent color of tablet (*n* = 21, 44.7%), followed by red/pink (*n* = 9, 19.1%), yellow (*n* = 6, 12.8%), green (*n* = 5, 10.6%), blue (*n* = 2, 4.3%), and purple (*n* = 1, 2.1%), with different shades of these colors observed. There were also three tablets that were white with a colored coating (2 blue and 1 orange), giving them a more commercial appearance. The same colors were found for powders containing AAS, including white (*n* = 2, 28.6%), blue (*n* = 2, 28.6%), pink (*n* = 1, 14.3%), yellow (*n* = 1, 14.3%), and orange (*n* = 1, 14.3%).

Interestingly, all but one of the yellow tablets were found to contain oxandrolone, and the yellow powder contained oxandrolone and stanozolol. This indicates yellow dye is often indicative of the presence of oxandrolone, possibly due to illicit producers trying to emulate one of the most popular brands of oxandrolone, Anavar™, which is often produced as yellow tablets.^51^

Excluding four samples that only contained tablet fragments where markings were unable to be determined, most AAS tablets had a score on one side (*n* = 27, 62.8%) or no visible markings (*n* = 6, 14.0%). The 10 remaining tablets had a variety of different tablet markings, the details of which can be found in the Supporting Information. Six of these tablets with a different tablet marking contained oxymetholone mixed with another drug, including amitriptyline, zopiclone, mirtazapine, and sertraline, so the markings may be reminiscent of tablet markings for these drugs. For example, two of these samples found to contain oxymetholone and amitriptyline were round in shape and had a blue coating inscribed with the letter “D” (e.g., Figure 2d) or “AA” on one side and “C” on the other. Previously, similar tablets have typically been found to only contain amitriptyline (*n* = 4 and *n* = 8, respectively, seized from the Scottish Prisons from 2019–2023), indicating that these were pharmaceutical tablets and the AAS detected was likely due to cross‐contamination from being stored or transported by prisoners with AASs. Two oxymetholone tablets were green with the marking “SHREE” on one side with a score on the other, as can be seen in Figure 2e. “SHREE” may be in reference to a manufacturer of AAS tablets in India, Shree Venkatesh International Limited^52^; however, details of the tablets produced by this company were unable to be found for comparison.

### 3.2 Quantitative results

**FIGURE 3:**
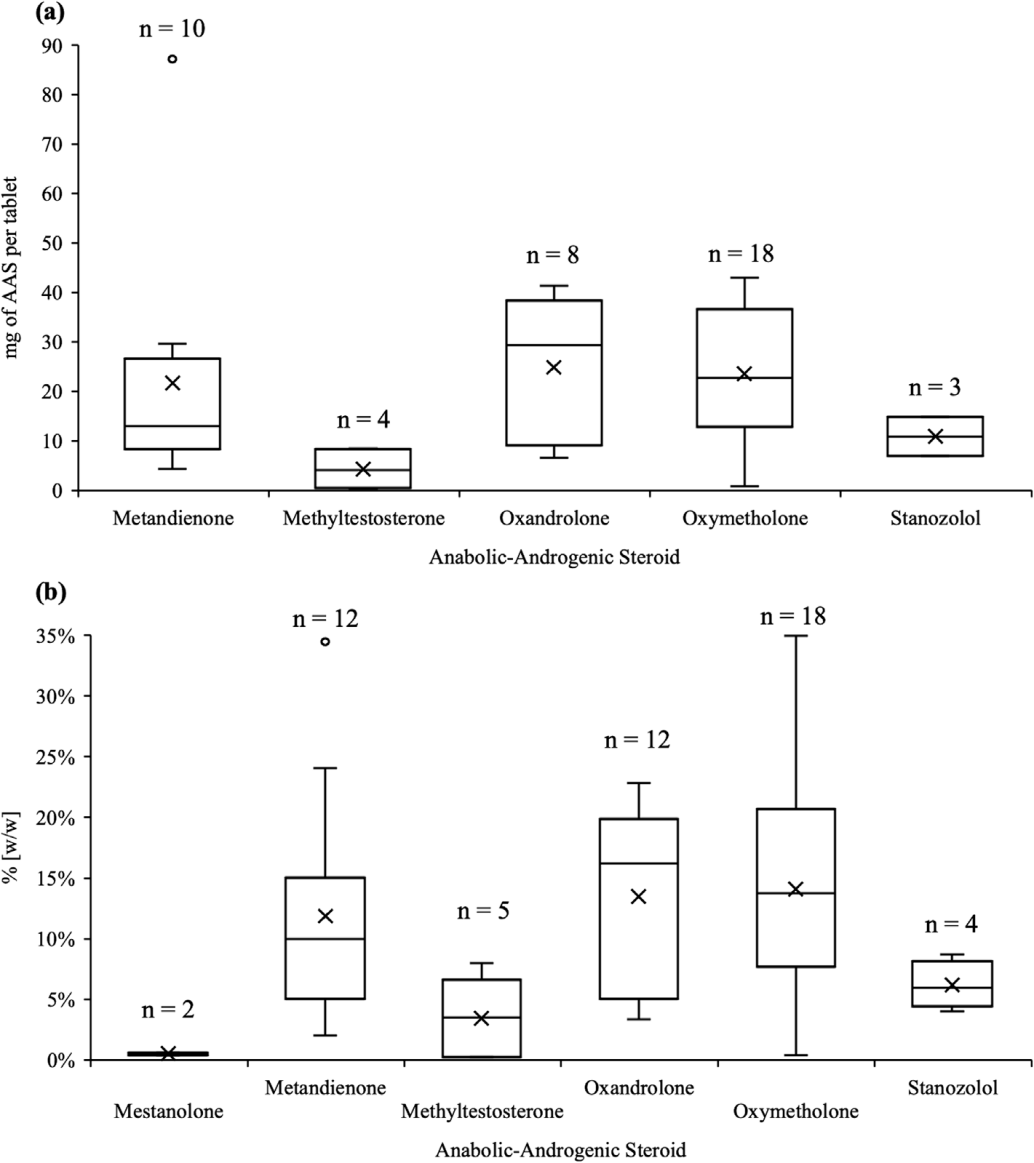
(a) mg of anabolic‐androgenic steroid (AAS) per tablet in samples seized from the Scottish prisons (*n* = 44). Some samples only contained tablet fragments (*n* = 4), so the total mass of the tablet, and thereby mg of AAS per tablet, was unable to be determined. (b) Concentrations (% w/w) of the AASs in tablet and powder samples seized from Scottish prisons (*n* = 53). Three samples were not quantitated due to an insufficient amount of sample material remaining after qualitative analysis. There were also 23 instances where the AAS was present at levels below the limit of quantitation.

The complete quantitative results can be found in Table S11.1. An overview of the quantitative results is provided in Table 1 and Figure 3. Three samples were unable to be quantified due to an insufficient sample remaining after qualitative analysis. Based on the size of the individual tablets, which ranged from 0.10 to 1.52 g with an average of 0.24 g, there was an average of 0.52 mg of mestanolone per tablet (0.51% w/w; *n* = 2), 21.67 ± 24.42 mg of metandienone per tablet (11.87 ± 9.25% w/w; *n* = 12), 4.31 ± 4.37 mg of methyltestosterone per tablet (3.45 ± 3.32% w/w; *n* = 5), 24.86 ± 14.44 mg of oxandrolone per tablet (13.45 ± 7.87% w/w; *n* = 8), 23.59 ± 14.08 mg of oxymetholone per tablet (14.07 ± 9.01% w/w; *n* = 18), and 10.90 ± 3.92 mg of stanozolol per tablet (6.09 ± 2.39% w/w; *n* = 3).

**TABLE 1:** Quantitation of anabolic‐androgenic steroid content in samples seized from Scottish prisons (July 2022 to July 2023) alongside adult daily doses for medical purposes according to the National Institutes of Health (NIH)^53^ and recreational purposes according to the Underground Steroid Handbook (USH).^54^ *n* is the number of samples quantitated with values above the LOQ included in the statistics, whereas *n* < LOQ is the number of samples quantitated with values below the LOQ. Five samples were not able to be quantified due to an insufficient amount of the sample material remaining after the initial qualitative analysis. The concentrations of AASs detected in mixtures were analyzed alongside solo detections in this table.

AAS	Daily dose		Format	Quantitation mg/tablet (% [w/w])					*n* < LOQ
	NIH	USH		*n*	Min	Max	Mean	SD	
Mestanolone	10–30 mg	‐	Tablet	2	0.47 (0.41)	0.58 (0.61)	0.52 (0.51)	‐	1
Metandienone	4 mg	20 mg	Tablet	12	4.30 (2.03)	87.21 (34.47)	21.67 (11.87)	24.42 (9.25)	3
Methyltestosterone	10–50 mg	‐	Tablet	5	0.40 (0.26)	8.54 (8.01)	4.31 (3.45)	4.37 (3.32)	9
Oxandrolone	2.5–20 mg	0.125 mg/lb	Tablet	8	6.56 (3.35)	41.41 (21.93)	24.86 (13.45)	14.44 (7.87)	1
	‐	‐	Powder	4	(6.55)	(22.81)	(13.54)	(7.16)	‐
Oxymetholone	1–5 mg/kg	25–150 mg	Tablet	18	0.85 (0.58)	43.01 (34.98)	23.59 (14.07)	14.08 (9.01)	9
Stanozolol	2–6 mg	‐	Tablet	3	7.01 (4.02)	10.84 (8.71)	10.90 (6.09)	3.92 (2.39)	‐
	‐	‐	Powder	1	(6.41)	‐	‐	‐	‐

Abbreviations: AAS, anabolic‐androgenic steroid; LOQ, limit of quantitation.

There were only powder samples of oxandrolone and stanozolol quantitated with an average of 13.54 ± 7.16% w/w for oxandrolone and 6.41% w/w for the stanozolol powder. Oxandrolone tablets and powders were not found to be statistically different (*t* test, *α* = 0.05, *p* = 0.98), indicating that concentrations were similar between tablets and powders. As there was only one stanozolol powder, statistical analysis was not possible for these samples, although the concentration of the powder was similar to the average stanozolol concentrations in tablets.

There were 23 cases where the AAS was below the LOQ. Of these, 12 (52.2%) were present in a mixture with one or more AAS, which had concentrations above the LOQ, and 7 (30.4%) were present in a mixture with another drug. Because 19 of the 23 samples containing an AAS below the LOQ were detected with another AAS or drug, it is likely that the AAS below the LOQ is present due to cross‐contamination, rather than purposeful addition.

A comparison of the concentrations of oxymetholone in samples where it was detected alone or in a mixture revealed a statistically significant difference (*t* test, *α* = 0.05, *p* = 0.014), where it was present at higher concentrations when in a mixture. In mixtures, there was an average concentration of 30.49 ± 10.37 mg of oxymetholone per tablet versus 14.66 ± 12.64 mg per tablet when it was present alone. Unfortunately, oxymetholone is the only AAS that had enough detections alone and in mixtures to allow for such a comparison. This provides some limited evidence that tablets with a mixture of AASs may be more dangerous, or produce more severe adverse effects, not only due to the polydrug use but also due to the drugs being present at higher concentrations.

Based on the medical dosing guidelines for oral AAS reported by the National Institutes of Health (NIH)^53^ and the reported typical dose for recreational use according to the Underground Steroid Handbook^54^ (see Table 1), the illicit tablets from the Scottish prisons containing mestanolone or methyltestosterone had concentrations below the lowest recommended daily dose, and oxymetholone tablet concentrations were at the low end of the reported typical recreational dose. On the other hand, tablets containing metandienone and stanozolol had concentrations above the recommended medical daily dose. Four metandienone tablets also had concentrations greater than the typical recreational dose (20 mg) as well, one of which was over four times greater (87.21 mg/tablet). Finally, all oxandrolone tablets were above the typical recreational dose based on the weight of an average male, and five tablets had more than the highest recommended medical daily dose.

## 4 CONCLUSIONS

To the best of the authors' knowledge, this is one of the first studies to report the qualitative and quantitative analysis of a large set of seized samples found to contain AASs. Although the detections of AASs in the Scottish prisons have been increasing since 2021, the compounds detected have remained stable with oxymetholone, metandienone, methyltestosterone, and oxandrolone dominating. The high prevalence of mixtures of AASs and other drugs provides further evidence of polydrug use among prisoners and people who use AASs. This demonstrates that AASs should be given more consideration within the forensic and criminal justice fields as they may contribute to criminality and adverse health outcomes, particularly in the context of polydrug use.

The quantitative results show that there is large variation in the concentration of AASs in the tablets and powders seized from the Scottish prisons, which is similar to results from the quantitation of other street‐prepared and illicit drugs.^33^, ^34^, ^55^, ^56^, ^57^ Although the AAS concentrations of illicit tablets and powders were often below or within the recommended medical daily dose, the large variability in the concentrations between samples increases the likelihood of harm from their use, particularly given prisoners are likely unaware of the concentrations of the AASs or maybe even what AAS they are taking.

## CONFLICT OF INTEREST STATEMENT

The authors do not report any conflicts of interest.

## Supporting information


**Table S1.1:** Analytical data for calculation of extraction efficiency using methanol for all drugs, except oxymetholone, which used acetonitrile.
**Table S2.**1 Mestanolone calibration curve data for 22/09/2023
**Table S2.**2 Mestanolone Quality Assurance Data for 22/09/2023
**Table S2.**3: Full analytical data for calculation of limit of detection
**Table S2.**4 Analytical data for calculation of lower limit of quantitation.
**Table S2.**5 Check standard data for bias calculations.
**Table S2.**6 Mestanolone low check standard data for precision calculations (40 μg/ml)
**Table S2.**7 Mestanolone medium check standard data for precision calculations (125 μg/ml)
**Table S2.**8 Mestanolone high check standard data for precision calculations (225 μg/ml)
**Table S3.**1 Methyltestosterone calibration curve data for 22/09/2023
**Table S3.**2 Methyltestosterone Quality Assurance Data for 22/09/2023
**Table S3.**3: Full analytical data for calculation of limit of detection
**Table S3.**4 Analytical data for calculation of lower limit of quantitation.
**Table S3.**5 Methyltestosterone check standard data for bias calculations.
**Table S3.**6 Low check standard data for precision calculations (40 μg/ml)
**Table S3.**7 Medium check standard data for precision calculations (125 μg/ml)
**Table S3.**8 High check standard data for precision calculations (225 μg/ml)
**Table S4.**1 Metandienone calibration curve data for 18/10/2023
**Table S4.**2 Metandienone Quality Assurance Data for 18/10/2023
**Table S4.**3: Full analytical data for calculation of limit of detection
**Table S4.**4 Analytical data for calculation of lower limit of quantitation.
**Table S4.**5 Metandienone check standard data for bias calculations.
**Table S4.**6 Low check standard data for precision calculations (40 μg/ml)
**Table S4.**7 Medium check standard data for precision calculations (125 μg/ml)
**Table S4.**8 High check standard data for precision calculations (225 μg/ml)
**Table S5.**1 Stanozolol calibration curve data for 25/10/2023
**Table S5.**2 Stanozolol Quality Assurance Data for 25/10/2023
**Table S5.**3: Full analytical data for calculation of limit of detection
**Table S5.**4 Analytical data for calculation of lower limit of quantitation.
**Table S5.**5 Stanzolol check standard data for bias calculations.
**Table S4.**6 Low check standard data for precision calculations (40 μg/ml)
**Table S4.**7 Medium check standard data for precision calculations (125 μg/ml)
**Table S4.**8 High check standard data for precision calculations (225 μg/ml)
**Table S6.**1 Oxymetholone calibration curve data for 07/11/2023
**Table S6.**2 Oxymetholone Quality Assurance Data for 07/11/2023
**Table S6.**3: Full analytical data for calculation of limit of detection
**Table S6.**4 Analytical data for calculation of lower limit of quantitation.
**Table S6.**5 Oxymetholone check standard data for bias calculations.
**Table S6.**6 Low check standard data for precision calculations (60 μg/ml)
**Table S6.**7 Medium check standard data for precision calculations (125 μg/ml)
**Table S6.**8 High check standard data for precision calculations (225 μg/ml)
**Table S7.**1 Oxandrolone calibration curve data for 10/11/2023
**Table S7.**2 Oxandrolone Quality Assurance Data for 10/11/2023
**Table S4.**3: Full analytical data for calculation of limit of detection
**Table S7.**4 Analytical data for calculation of lower limit of quantitation.
**Table S7.**5 Oxandrolone check standard data for bias calculations.
**Table S7.**6 Low check standard data for precision calculations (40 μg/ml)
**Table S7.**7 Medium check standard data for precision calculations (125 μg/ml)
**Table S7.**8 High check standard data for precision calculations (225 μg/ml)
**Table S8.1.** Full AAS qualitative data for seized samples from GC–MS analysis, including retention time (RT), RT of reference (ref) standard, MS library R‐Match, and mass‐to‐charge ratio (m/z). Due to security concerns, the prison establishment is provided as a number.
**Table S10.1.** Full tablet markings data for samples of tablets found to contain AASs seized in Scottish prisons. For samples that only contained fragments, so the shape and markings of the tablets could not be determined, “NA” is written in the table. “opp” indicates marking on opposite side of the tablet.
**Table S11.1.** Full AAS quantitation data for seized samples.
**Figure S2.**1 Mestanolone calibration curve from 22/09/2023
**Figure S3.**1 Methyltestosterone calibration curve from 22/09/2023
**Figure S4.**1 Metandienone calibration curve from 18/10/2023
**Figure S5.**1 Stanozolol calibration curve from 25/10/2023
**Figure S6.**1 Oxymetholone calibration curve from 07/11/2023
**Figure S7.**1 Oxandrolone calibration curve from 10/11/2023
**Figure S9.1.** Examples of other sample types seized from the Scottish prisons found positive for AASs: (a) green powder seized 7th February 2022 found positive for oxymetholone and mestanolone; (b) blue powder seized 3rd April 2023 found positive for oxandrolone; (c) herbal material seized 11th March 2023 found positive for metandienone and Δ^9^‐THC; and (d) pieces of crushed soap bar‐like material seized 15th February 2023 found positive for mestanolone.
