# Supplementary material for: Changing trends in anabolic‐androgenic steroid use within Scottish prisons: Detection, prevalence, and quantitation
Source: Drug Test Anal. 2024 Aug 20;17(6):858–67. doi: 10.1002/dta.3790 (PMC12151717; doi:10.1002/dta.3790)
Supplement: Supplementary file 1 — Table S1.1: Analytical data for calculation of extraction efficiency using methanol for all drugs, except oxymetholone, which used acetonitrile. Table S2.1 Mestanolone calibration curve data for 22/09/2023 Table S2.2 Mestanolone Quality Assurance Data for 22/09/2023 Table S2.3: Full analytical data for calculation of limit of detection Table S2.4 Analytical data for calculation of lower limit of quantitation. Table S2.5 Check standard data for bias calculations. Table S2.6 Mestanolone low check standard data for precision calculations (40 μg/ml) Table S2.7 Mestanolone medium check standard data for precision calculations (125 μg/ml) Table S2.8 Mestanolone high check standard data for precision calculations (225 μg/ml) Table S3.1 Methyltestosterone calibration curve data for 22/09/2023 Table S3.2 Methyltestosterone Quality Assurance Data for 22/09/2023 Table S3.3: Full analytical data for calculation of limit of detection Table S3.4 Analytical data for calculation of lower limit of quantitation. Table S3.5 Methyltestosterone check standard data for bias calculations. Table S3.6 Low check standard data for precision calculations (40 μg/ml) Table S3.7 Medium check standard data for precision calculations (125 μg/ml) Table S3.8 High check standard data for precision calculations (225 μg/ml) Table S4.1 Metandienone calibration curve data for 18/10/2023 Table S4.2 Metandienone Quality Assurance Data for 18/10/2023 Table S4.3: Full analytical data for calculation of limit of detection Table S4.4 Analytical data for calculation of lower limit of quantitation. Table S4.5 Metandienone check standard data for bias calculations. Table S4.6 Low check standard data for precision calculations (40 μg/ml) Table S4.7 Medium check standard data for precision calculations (125 μg/ml) Table S4.8 High check standard data for precision calculations (225 μg/ml) Table S5.1 Stanozolol calibration curve data for 25/10/2023 Table S5.2 Stanozolol Quality Assurance Data for 25/10/2023 Table S5.3 [file DTA-17-858-s001.docx]

### **Changing trends in anabolic-androgenic steroid use within Scottish prisons: Detection, prevalence, and quantitation**

**Supplementary Information**

**Section 1: Extraction efficiency for anabolic-androgenic steroid quantitation method**

**Section 2: Method validation data for mestanolone quantitation method**

**Section 3: Method validation data for methyltestosterone quantitation method**

**Section 4: Method validation data for metandienone quantitation method**

**Section 5: Method validation data for stanozolol quantitation method**

**Section 6: Method validation data for oxymetholone quantitation method**

**Section 7: Method validation data for oxandrolone quantitation method**

**Section 8: Full qualitative data for samples found to contain AASs seized in Scottish prisons.**

**Section 9: Example photographs of other samples types seized from the Scottish prisons found positive for AASs.**

**Section 10: Full tablet markings data for samples of tablets found to contain AASs seized in Scottish prisons.**

**Section 11: Full quantitative data for samples found to contain AASs seized in Scottish prisons.**

**Section 12: R Script for quantitation**

**Section 1: Extraction efficiency for anabolic-androgenic steroid quantitation method**

To ensure near-exhaustive extraction of the AAS compounds from test samples, sequential extractions in methanol, or acetonitrile for oxymetholone, were carried out using representative tablet samples known to contain each AAS. Samples were extracted using 1 min centrifuging. These were then analyzed using the quantitative GC-MS method. Five sequential extractions in methanol were found to be sufficient to extract all detectable mestanolone, methyltestosterone, stanozolol, and metandienone and >99% for oxandrolone. Six sequential extractions in acetonitrile were found to be sufficient to extract >99% for oxymetholone.

Table S1.1: Analytical data for calculation of extraction efficiency using methanol for all drugs, except oxymetholone, which used acetonitrile.

| **Drug** | **Extraction** | **RT** | **Peak Area** | **% Extracted** | **Total %** **extracted** |
| --- | --- | --- | --- | --- | --- |
| Mestanolone | 1 | 7.514 | 37659664 | 76.31% | 76.31% |
|  | 2 | 7.510 | 6854354 | 13.89% | 90.19% |
|  | 3 | 7.510 | 2988589 | 6.06% | 96.25% |
|  | 4 | 7.510 | 1211730 | 2.46% | 98.70% |
|  | 5 | 7.512 | 639128 | 1.30% | 100.00% |
|  | 6 | 0 | 0 | 0.00% | - |
| Methyltestosterone | 1 | 7.811 | 4934901 | 92.76% | 92.76% |
|  | 2 | 7.810 | 385014 | 7.24% | 100.00% |
|  | 3 | 0 | 0 | 0.00% | - |
|  | 4 | 0 | 0 | 0.00% | - |
|  | 5 | 0 | 0 | 0.00% | - |
| Stanozolol | 1 | 10.208 | 69925036 | 55.97% | 55.97% |
|  | 2 | 10.206 | 34030327 | 27.24% | 83.20% |
|  | 3 | 10.205 | 15238545 | 12.20% | 95.40% |
|  | 4 | 10.206 | 4844746 | 3.88% | 99.28% |
|  | 5 | 10.208 | 904532 | 0.72% | 100.00% |
|  | 6 | 0 | 0 | 0.00% |  |
| Oxymetholone | 1 | 8.411 | 731422649 | 49.94% | 49.94% |
|  | 2 | 8.384 | 435028376 | 29.71% | 79.65% |
|  | 3 | 8.354 | 154128804 | 10.52% | 90.17% |
|  | 4 | 8.342 | 75038647 | 5.12% | 95.30% |
|  | 5 | 8.339 | 38917105 | 2.66% | 97.96% |
|  | 6 | 8.334 | 20043556 | 1.37% | 99.32% |
|  | 7 | 8.334 | 7048491 | 0.48% | 99.81% |
|  | 8 | 8.337 | 742908 | 0.05% | 99.86% |
|  | 9 | 9.339 | 2106246 | 0.14% | 100.00% |
|  | 10 | 0 | 0 | 0.00% |  |
| Metandienone | 1 | 8.043 | 860659309 | 78.60% | 78.60% |
|  | 2 | 7.984 | 215513070 | 19.68% | 98.28% |
|  | 3 | 7.957 | 9617765 | 0.88% | 99.15% |
|  | 4 | 7.956 | 8883481 | 0.81% | 99.97% |
|  | 5 | 7.955 | 376774 | 0.03% | 100.00% |
|  | 6 | 0 | 0 | 0.00% | - |
| Oxandrolone | 1 | 8.335 | 1120526515 | 39.90% | 39.90% |
|  | 2 | 8.293 | 618325197 | 22.02% | 61.92% |
| Oxandrolone | 3 | 8.274 | 423833059 | 15.09% | 77.01% |
|  | 4 | 8.288 | 568936614 | 20.26% | 97.27% |
|  | 5 | 8.227 | 57973497 | 2.06% | 99.34% |
|  | 6 | 8.224 | 17886068 | 0.64% | 99.98% |
|  | 7 | 8.219 | 504701 | 0.02% | 99.99% |
|  | 8 | 8.216 | 160460 | 0.01% | 100.00% |
|  | 9 | 0 | 0 | 0.00% | - |

**Section 2: Method validation data for mestanolone quantitation method**

**2.1 Example Calibration Data**

Table S2.1 Mestanolone calibration curve data for 22/09/2023

| **Concentration (µg/mL)** | **Internal Standard Peak Area** | **Mestanolone Peak Area** | **Peak Area Ratio** |
| --- | --- | --- | --- |
| 25 | 15093293 | 39379198 | 0.38 |
| 50 | 35831508 | 43244698 | 0.83 |
| 75 | 57271926 | 42564439 | 1.35 |
| 100 | 78719386 | 44745184 | 1.76 |
| 150 | 127540590 | 45192170 | 2.82 |
| 200 | 162178875 | 47096117 | 3.44 |
| 250 | 199446389 | 50660210 | 3.94 |


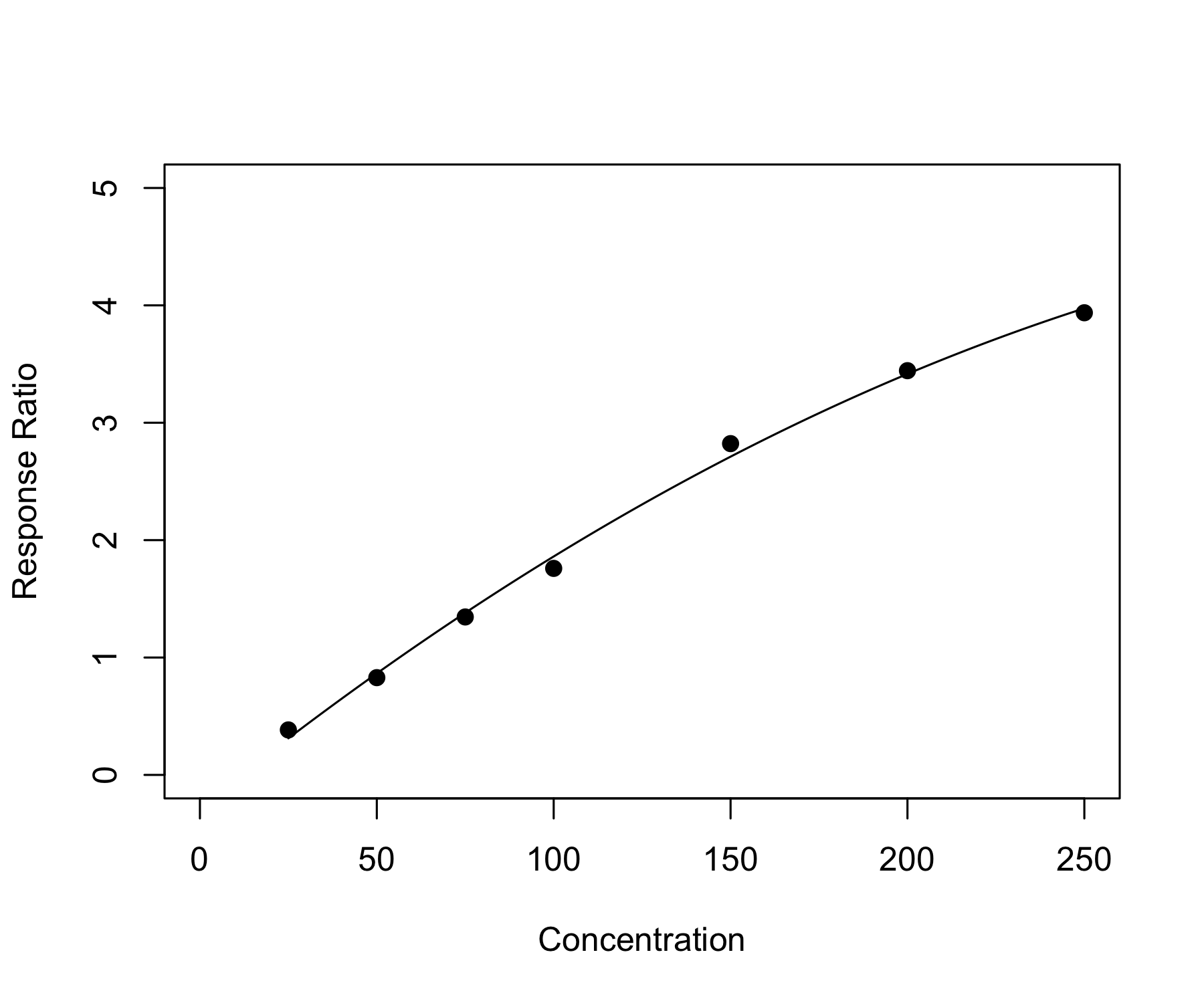


y = (-2.9×10^-5^)x^2^ + 0.024x – 0.28

R^2^ = 0.997

Figure S2.1 Mestanolone calibration curve from 22/09/2023

Table S2.2 Mestanolone Quality Assurance Data for 22/09/2023

| **Check Standard Concentration (µg/mL)** | **Mestanolone Peak Area** | **Internal Standard Peak Area** | **Peak Area Ratio** | **Calculated Concentration (µg/mL)** | **Bias**  **(%)** |
| --- | --- | --- | --- | --- | --- |
| 40 | 28588097 | 42171713 | 0.68 | 41.37 | 3.44 |
| 40 | 31248532 | 46624721 | 0.67 | 41.02 | 2.56 |
| 125 | 110022444 | 47692615 | 2.31 | 125.16 | 0.13 |
| 125 | 113460708 | 50338210 | 2.25 | 122.06 | 2.35 |

**2.2 Limit of Detection (LOD)**

To determine the limit of detection, mestanolone solutions were prepared at 5, 10, 15 and 20 μg/mL. Each solution was analyzed in replicate five times and the signal to noise ratio calculated. For each replicate, the mestanolone peak was compared to the SWGDRUG mass spectral library (version 3.11, released 1 June 2022), and the (reverse) match factor was required to be greater than 850/1,000 for positive identification.

Table S2.3: Full analytical data for calculation of limit of detection

| **Concentration (µg/mL)** | **Injection** | **Max Peak Height** | **Max Noise Height** | **R Match** | **Signal to Noise Ratio** |
| --- | --- | --- | --- | --- | --- |
| 5 | 1 | 74342 | 23469 | 849 | 3.17 |
|  | 2 | 31473 | 12532 | 720 | 2.51 |
|  | 3 | 20607 | 9334 | 622 | 2.21 |
|  | 4 | 12666 | 6199 | 502 | 2.04 |
|  | 5 | 8594 | 5116 | 464 | 1.68 |
| 10 | 1 | 146379 | 24664 | 852 | 5.93 |
|  | 2 | 76971 | 12396 | 851 | 6.21 |
|  | 3 | 51299 | 7932 | 805 | 6.47 |
|  | 4 | 33428 | 5439 | 760 | 6.15 |
|  | 5 | 24255 | 4019 | 686 | 6.04 |
| 15 | 1 | 194701 | 17688 | 911 | 11.01 |
|  | 2 | 115287 | 11927 | 858 | 9.67 |
|  | 3 | 77961 | 8304 | 854 | 9.39 |
|  | 4 | 48956 | 6258 | 793 | 7.82 |
|  | 5 | 39422 | 3799 | 752 | 10.38 |
| 20 | 1 | 299290 | 18071 | 918 | 16.56 |
|  | 2 | 175526 | 13788 | 882 | 12.73 |
|  | 3 | 120510 | 9489 | 844 | 12.70 |
|  | 4 | 82427 | 5786 | 832 | 14.25 |
|  | 5 | 59902 | 4202 | 795 | 14.26 |

Evaluation of the results demonstrated an acceptable signal to noise ratio above 10 μg/mL (>3.3), however these replicates did not meet the requirements specified for positive identification of mestanolone. Therefore, the LOD for this method was determined to be 20 μg/mL.

**2.3 Lower Limit of Quantitation (LLOQ)**

LLOQ was defined using the lowest non-zero calibrator. The 25 μg/mL calibration standard was analyzed across 5 separate runs and the bias was evaluated. As defined in Standard 036 (25), to be accepted, the bias of these replicates was required to fall below 20%.

Table S2.4 Analytical data for calculation of lower limit of quantitation.

| **Nominal Concentration (µg/mL)** | **Calculated Concentration (µg/mL)** |
| --- | --- |
| 25 | 28.30 |
| 25 | 26.96 |
| 25 | 28.15 |
| 25 | 27.08 |
| 25 | 28.61 |
|  |  |
| **Grand Mean** | 27.82 |
| **Bias** | 11.28% |

**Equation 2.1: Calculation of Bias (%)**

$$Bias \left( \% \right)=\left\{ \frac{Grand Mean of Calculated Concentration-Nominal Concentration}{Nominal Concentration} \right\} \times100$$

The LLOQ was defined as the lowest non-zero calibrator at 25 μg/mL with bias within 11.28% of the nominal concentration.

| **2.4 Calculation of Bias** |  |  |
| --- | --- | --- |

To evaluate bias, low, medium and high check standards of mestanolone were analyzed over five separate runs. Each run was carried out on a different day, with a new calibration curve. Each check standard was analyzed in triplicate within each run, giving a total of 15 replicates for each concentration. The maximum acceptable bias for each concentration was defined as ±20%. This method was determined to be accurate with bias within ­­±17% across all check standards.

Table S2.5 Check standard data for bias calculations.

|  | **Low Check Standard (40 µg/mL)** | | **Medium Check Standard (125 µg/mL)** | | **High Check Standard (225 µg/mL)** | |
| --- | --- | --- | --- | --- | --- | --- |
|  | **Calculated Conc.** | **Bias** | **Calculated Conc.** | **Bias** | **Calculated Conc.** | **Bias** |
| **Run 1** | 41.54 | 3.85 | 118.98 | 4.82 | 249.45 | 10.87 |
|  | 42.35 | 5.87 | 128.45 | 2.76 | 232.90 | 3.51 |
|  | 41.34 | 3.35 | 127.97 | 2.37 | 253.60 | 12.71 |
| **Run 2** | 41.37 | 3.44 | 125.16 | 0.13 | 244.70 | 8.76 |
|  | 41.02 | 2.56 | 122.06 | 2.35 | 237.37 | 5.50 |
|  | 40.23 | 0.58 | 122.53 | 1.97 | 253.44 | 12.64 |
| **Run 3** | 41.43 | 3.58 | 127.74 | 2.19 | 247.68 | 10.08 |
|  | 41.04 | 2.60 | 133.18 | 6.54 | 253.43 | 12.64 |
|  | 42.09 | 5.22 | 132.85 | 6.28 | 253.74 | 12.77 |
| **Run 4** | 38.38 | 4.05 | 118.44 | 5.25 | 207.18 | 7.92 |
|  | 37.33 | 6.67 | 115.01 | 7.99 | 211.31 | 6.09 |
|  | 36.03 | 9.91 | 116.72 | 6.62 | 212.61 | 5.51 |
| **Run 5** | 43.34 | 8.35 | 126.50 | 1.20 | 261.13 | 16.06 |
|  | 42.71 | 6.77 | 128.03 | 2.42 | 258.92 | 15.07 |
|  | 44.49 | 11.23 | 126.49 | 1.19 | 256.06 | 13.81 |
|  |  |  |  |  |  |  |
| **Grand Mean** | 41.07 | 5.24 | 124.91 | 3.55 | 241.65 | 9.84 |

**2.5 Calculation of Precision**

To evaluate precision, low, medium and high check standards were analyzed over five separate runs. Each run was carried out on a different day, with a new calibration curve. Each check standard was analyzed in triplicate within each run, giving a total of 15 replicates for each concentration. Within-run and between-run precision was calculated as CV% using a single factor ANOVA. The maximum acceptable CV% for each concentration was defined as 20%. This method was determined reproducible with precision determined as being within 3% for within-run results and 17% for between-run results.

Within-run precision was calculated for each concentration using Equation 2.2, where MS_wg_ is the mean square within groups obtained from the ANOVA table.

**Equation 2.2: Calculation of Within-Group Precision.**

$$Within-Run CV \left( \% \right)= \left[ \frac{\sqrt{{MS}_{wg}}}{Grand Mean for each concentration} \right]\times100$$

Between-run precision was calculated for each concentration using Equation 2.3, where MS_bg_ is the mean square between groups obtained from the ANOVA table and *n* is the number of observations in each group.

**Equation 2.3: Calculation of Between-Group Precision.**

$$Between-Run CV \left( \% \right)= \left[ \frac{\frac{\sqrt{{MS}_{bg}}+\left( n-1 \right) \times{MS}_{wg}}{n}}{Grand Mean for each concentration} \right]\times100$$

Table 2.6 Mestanolone low check standard data for precision calculations (40 µg/mL)

| Run 1  (µg/mL) | Run 2  (µg/mL) | Run 3  (µg/mL) | Run 4  (µg/mL) | Run 5  (µg/mL) |
| --- | --- | --- | --- | --- |
| 41.54 | 41.37 | 41.43 | 38.38 | 43.34 |
| 42.35 | 41.02 | 41.04 | 37.33 | 42.71 |
| 41.34 | 40.23 | 42.09 | 36.03 | 44.49 |

Anova: Single Factor

| Groups | Count | Sum | Average | Variance |
| --- | --- | --- | --- | --- |
| Run 1 | 3.00 | 125.23 | 41.74 | 0.29 |
| Run 2 | 3.00 | 122.62 | 40.87 | 0.34 |
| Run 3 | 3.00 | 124.56 | 41.52 | 0.28 |
| Run 4 | 3.00 | 111.75 | 37.25 | 1.38 |
| Run 5 | 3.00 | 130.54 | 43.51 | 0.81 |

| **Grand Mean (µg/mL)** | 41.07 |
| --- | --- |
| **Within Run Precision** | 1.92% |
| **Between Run Precision** | 4.25% |

| Source of Variation | SS | df | MS | F | P-value | F crit |
| --- | --- | --- | --- | --- | --- | --- |
| Between Groups | 63.68 | 4.00 | 15.92 | 25.65 | 0.00 | 3.48 |
| Within Groups | 6.21 | 10.00 | 0.62 |  |  |  |
| Total | 69.89 | 14.00 |  |  |  |  |

Table 2.7 Mestanolone medium check standard data for precision calculations (125 µg/mL)

| Run 1  (µg/mL) | Run 2  (µg/mL) | Run 3  (µg/mL) | Run 4  (µg/mL) | Run 5  (µg/mL) |
| --- | --- | --- | --- | --- |
| 118.98 | 125.16 | 127.74 | 118.44 | 126.50 |
| 128.45 | 122.06 | 133.18 | 115.01 | 128.03 |
| 127.97 | 122.53 | 132.85 | 116.72 | 126.49 |

Anova: Single Factor

| Groups | Count | Sum | Average | Variance |
| --- | --- | --- | --- | --- |
| Run 1 | 3.00 | 375.40 | 125.13 | 28.46 |
| Run 2 | 3.00 | 369.75 | 123.25 | 2.79 |
| Run 3 | 3.00 | 393.77 | 131.26 | 9.30 |
| Run 4 | 3.00 | 350.17 | 116.72 | 2.94 |
| Run 5 | 3.00 | 381.02 | 127.01 | 0.79 |

| **Grand Mean (µg/mL)** | 124.91 |
| --- | --- |
| **Within Run Precision** | 2.38% |
| **Between Run Precision** | 7.20% |

| Source of Variation | SS | df | MS | F | P-value | F crit |
| --- | --- | --- | --- | --- | --- | --- |
| Between Groups | 342.61 | 4.00 | 85.65 | 9.67 | 0.02 | 3.48 |
| Within Groups | 88.55 | 10.00 | 8.86 |  |  |  |
| Total | 431.16 | 14.00 |  |  |  |  |

Table 2.8 Mestanolone high check standard data for precision calculations (225 µg/mL)

| Run 1  (µg/mL) | Run 2  (µg/mL) | Run 3  (µg/mL) | Run 4  (µg/mL) | Run 5  (µg/mL) |
| --- | --- | --- | --- | --- |
| 249.45 | 244.7 | 247.68 | 207.18 | 261.13 |
| 232.90 | 237.37 | 253.43 | 211.31 | 258.92 |
| 253.60 | 253.44 | 253.74 | 212.61 | 256.06 |

Anova: Single Factor

| Groups | Count | Sum | Average | Variance |
| --- | --- | --- | --- | --- |
| Run 1 | 3.00 | 631.10 | 210.37 | 8.04 |
| Run 2 | 3.00 | 735.51 | 245.17 | 64.73 |
| Run 3 | 3.00 | 754.85 | 251.62 | 11.65 |
| Run 4 | 3.00 | 631.09 | 210.36 | 8.03 |
| Run 5 | 3.00 | 776.11 | 258.70 | 6.46 |

| **Grand Mean (µg/mL)** | 241.65 |
| --- | --- |
| **Within Run Precision** | 2.69% |
| **Between Run Precision** | 16.09% |

| Source of Variation | SS | df | MS | F | P-value | F crit |
| --- | --- | --- | --- | --- | --- | --- |
| Between Groups | 4179.21 | 4.00 | 1044.80 | 24.78 | 0.00 | 3.48 |
| Within Groups | 421.61 | 10.00 | 42.16 |  |  |  |
| Total | 4600.82 | 14.00 |  |  |  |  |

**2.6 Carryover**

To evaluate carryover, the highest mestanolone calibration standard (250 µg/mL) and subsequent methanol blank were analyzed over three separate runs. Each run was carried out on a different day, with a new calibration curve. This method was determined to produce no carryover.

A


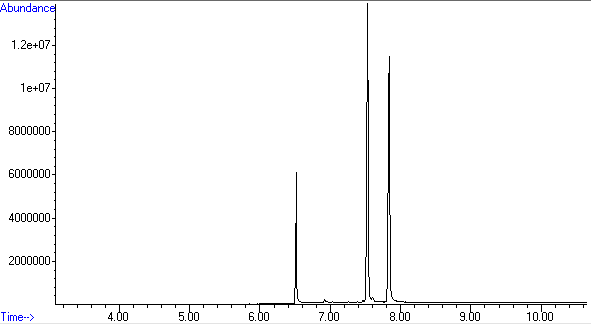

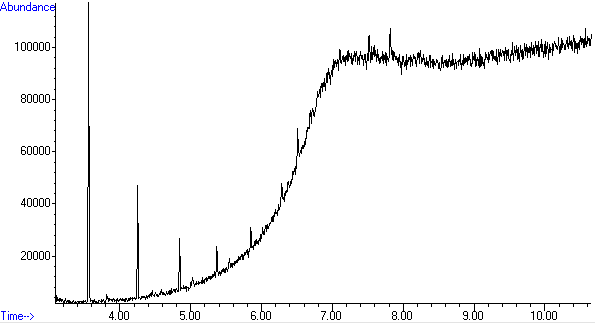


B


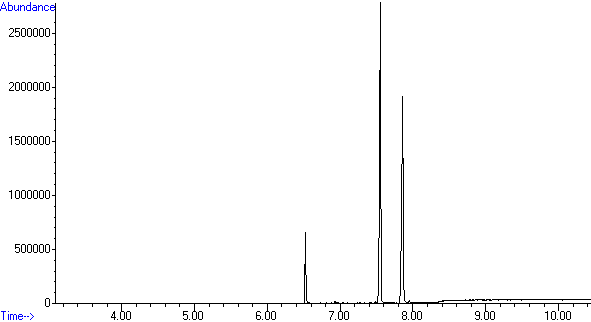

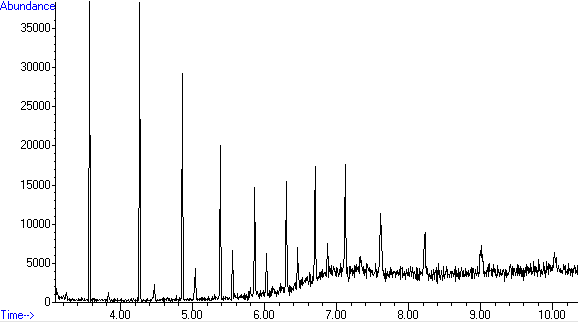


C


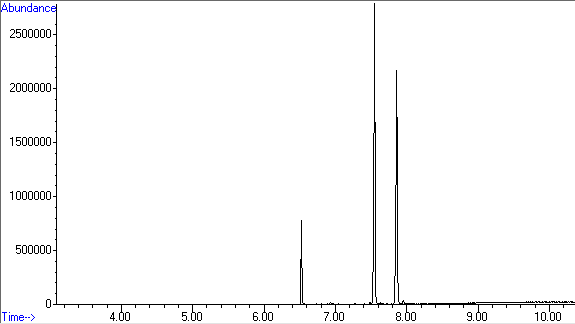

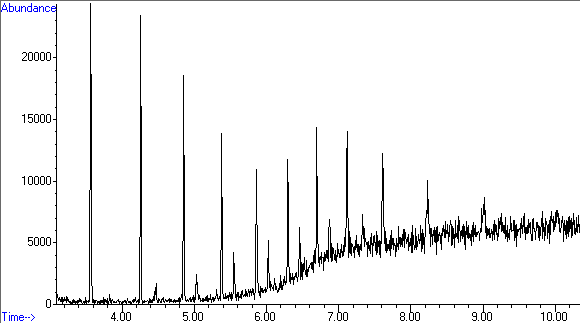


Figure S2.2 Mestanolone and methyltestosterone 250 µg/mL calibration standard (left) and subsequent methanol blank (right) run on three different days: (A) on 22-09-2023, (B) on 30-08-2023, and (C) on 29-08-2023.

**Section 3: Method validation data for methyltestosterone quantitation method**

**3.1 Example Calibration Data**

Table S3.1 Methyltestosterone calibration curve data for 22/09/2023

| **Concentration (µg/mL)** | **Internal Standard Peak Area** | **Methyltestosterone Peak Area** | **Peak Area Ratio** |
| --- | --- | --- | --- |
| 25 | 39379198 | 11484786 | 0.29 |
| 50 | 43244698 | 32095176 | 0.74 |
| 75 | 42564439 | 50278803 | 1.18 |
| 100 | 44745184 | 70205093 | 1.57 |
| 150 | 45192170 | 116065893 | 2.57 |
| 200 | 47096117 | 153323791 | 3.26 |
| 250 | 50660210 | 187416707 | 3.70 |


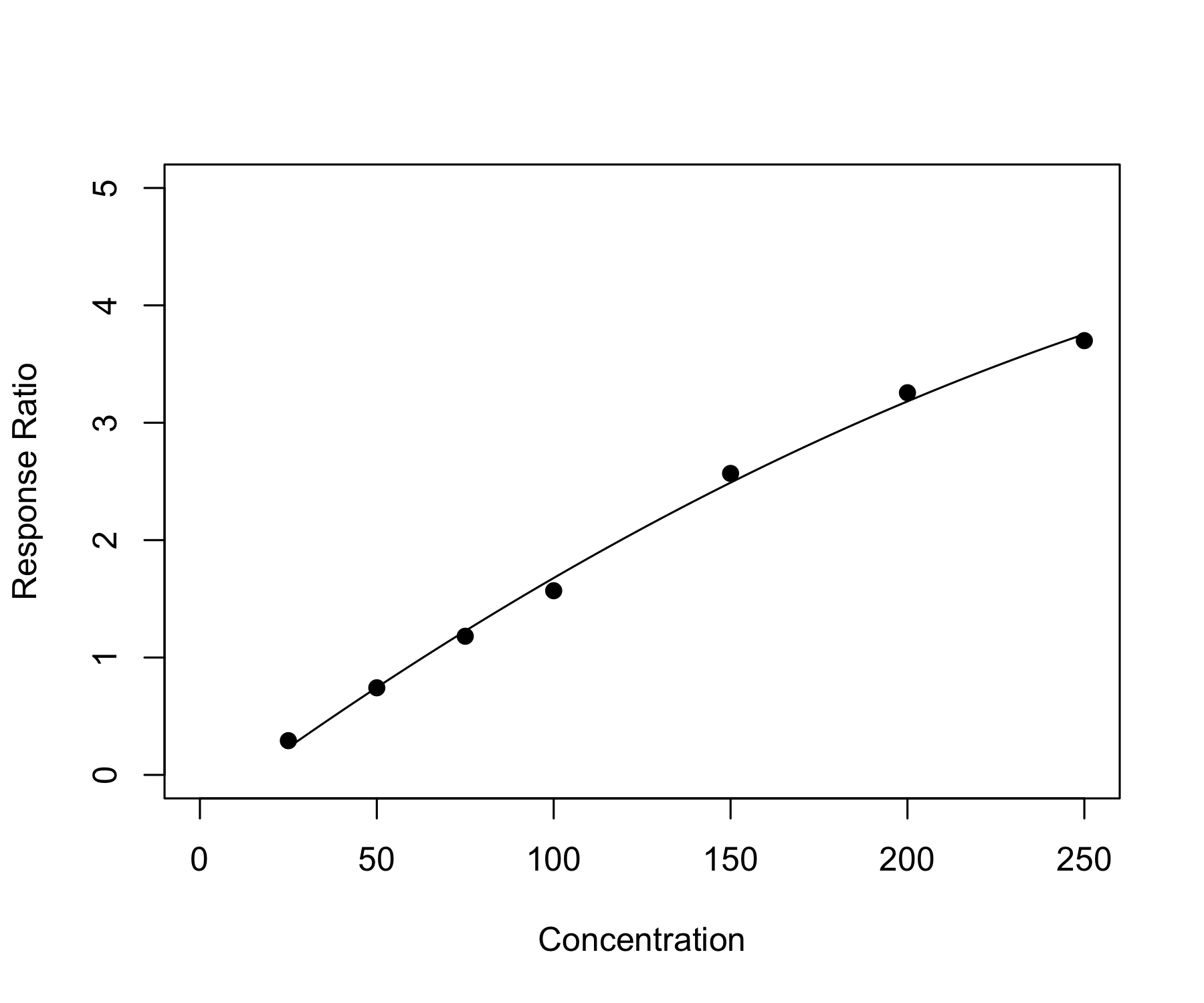


y = (-2.4×10^-5^)x^2^ + 0.022x – 0.31

R^2^ = 0.997

Figure S3.1 Methyltestosterone calibration curve from 22/09/2023

Table S3.2 Methyltestosterone Quality Assurance Data for 22/09/2023

| **Check Standard Concentration (µg/mL)** | **Methyltestosterone Peak Area** | **Internal Standard Peak Area** | **Peak Area Ratio** | **Calculated Concentration (µg/mL)** | **Bias**  **(%)** |
| --- | --- | --- | --- | --- | --- |
| 40 | 24744364 | 42171713 | 0.59 | 42.12 | 5.29 |
| 40 | 26415058 | 46624721 | 0.57 | 41.12 | 2.80 |
| 125 | 102708244 | 47692615 | 2.15 | 128.40 | 2.72 |
| 125 | 106184469 | 50338210 | 2.11 | 125.67 | 0.53 |

**3.2 Limit of Detection (LOD)**

To determine the limit of detection, methyltestosterone solutions were prepared at 5, 10, 15 and 20 μg/mL. Each solution was analyzed in replicate five times and the signal to noise ratio calculated. For each replicate, the methyltestosterone peak was compared to the SWGDRUG mass spectral library (version 3.11, released 1 June 2022), and the (reverse) match factor was required to be greater than 850/1,000 for positive identification.

Table S3.3: Full analytical data for calculation of limit of detection

| **Concentration (µg/mL)** | **Injection** | **Max Peak Height** | **Max Noise Height** | **R Match** | **Signal to Noise Ratio** |
| --- | --- | --- | --- | --- | --- |
| 5 | 1 | 27851 | 18209 | 618 | 1.53 |
|  | 2 | 16977 | 9613 | 568 | 1.77 |
|  | 3 | 9569 | 7192 | 495 | 1.33 |
|  | 4 | 7469 | 4257 | 401 | 1.75 |
|  | 5 | - | - | - | - |
| 10 | 1 | 47217 | 18600 | 799 | 2.54 |
|  | 2 | 31785 | 11348 | 715 | 2.80 |
|  | 3 | 18159 | 5556 | 691 | 3.27 |
|  | 4 | 13106 | 4674 | 597 | 2.80 |
|  | 5 | 9206 | 3292 | 504 | 2.80 |
| 15 | 1 | 94718 | 16721 | 822 | 5.66 |
|  | 2 | 61838 | 9122 | 813 | 6.78 |
|  | 3 | 38108 | 6063 | 786 | 6.29 |
|  | 4 | 28038 | 4579 | 742 | 6.12 |
|  | 5 | 22735 | 2901 | 696 | 7.84 |
| 20 | 1 | 140564 | 16720 | 863 | 8.41 |
|  | 2 | 86310 | 10288 | 852 | 8.39 |
|  | 3 | 64611 | 5920 | 829 | 10.91 |
|  | 4 | 42794 | 4064 | 792 | 10.53 |
|  | 5 | 36207 | 3399 | 763 | 10.65 |

Evaluation of the results demonstrated an acceptable signal to noise ratio above 10 μg/mL (>3.3), however these replicates did not meet the requirements specified for positive identification of methyltestosterone. Therefore, the LOD for this method was determined to be 20 μg/mL.

**3.3 Lower Limit of Quantitation (LLOQ)**

LLOQ was defined using the lowest non-zero calibrator. The 25 μg/mL calibration standard was analyzed across 5 separate runs and the bias was evaluated. As defined in Standard 036 (25), to be accepted, the bias of these replicates was required to fall below 20%.

Table S3.4 Analytical data for calculation of lower limit of quantitation.

| **Nominal Concentration (µg/mL)** | **Calculated Concentration (µg/mL)** |
| --- | --- |
| 25 | 26.81 |
| 25 | 29.47 |
| 25 | 27.78 |
| 25 | 25.75 |
| 25 | 28.43 |
|  |  |
| **Grand Mean** | 27.65 |
| **Bias** | 10.59 |

**Equation 3.1: Calculation of Bias (%)**

$$Bias \left( \% \right)=\left\{ \frac{Grand Mean of Calculated Concentration-Nominal Concentration}{Nominal Concentration} \right\} \times100$$

The LLOQ was defined as the lowest non-zero calibrator at 25 μg/mL with bias within 10.59% of the nominal concentration.

| **3.4 Calculation of Bias** |  |  |
| --- | --- | --- |

To evaluate bias, low, medium and high check standards for methyltestosterone were analyzed over five separate runs. Each run was carried out on a different day, with a new calibration curve. Each check standard was analyzed in triplicate within each run, giving a total of 15 replicates for each concentration. The maximum acceptable bias for each concentration was defined as ±20%. This method was determined to be accurate with bias within ­­±12% across all check standards.

Table S3.5 Methyltestosterone check standard data for bias calculations.

|  | **Low Check Standard (40 µg/mL)** | | **Medium Check Standard (125 µg/mL)** | | **High Check Standard (225 µg/mL)** | |
| --- | --- | --- | --- | --- | --- | --- |
|  | **Calculated Conc.** | **Bias** | **Calculated Conc.** | **Bias** | **Calculated Conc.** | **Bias** |
| **Run 1** | 42.02 | 5.06 | 125.63 | 0.51 | 239.88 | 6.61 |
|  | 42.34 | 5.85 | 136.07 | 8.86 | 224.88 | 0.05 |
|  | 42.01 | 5.03 | 136.04 | 8.83 | 243.54 | 8.24 |
| **Run 2** | 38.38 | 4.04 | 117.06 | 6.35 | 225.96 | 0.43 |
|  | 39.66 | 0.86 | 115.53 | 7.57 | 228.02 | 1.34 |
|  | 39.09 | 2.28 | 118.93 | 4.85 | 233.74 | 3.88 |
| **Run 3** | 42.12 | 5.29 | 128.40 | 2.72 | 234.56 | 4.25 |
|  | 41.12 | 2.80 | 125.67 | 0.53 | 232.21 | 3.20 |
|  | 39.72 | 0.70 | 125.93 | 0.74 | 250.06 | 11.14 |
| **Run 4** | 40.67 | 1.69 | 130.99 | 4.79 | 239.28 | 6.35 |
|  | 39.50 | 1.24 | 137.12 | 9.69 | 245.91 | 9.29 |
|  | 41.50 | 3.75 | 137.70 | 10.16 | 247.98 | 10.21 |
| **Run 5** | 42.79 | 6.97 | 130.52 | 4.42 | 252.99 | 12.44 |
|  | 43.12 | 7.81 | 131.82 | 5.46 | 251.71 | 11.87 |
|  | 43.99 | 9.98 | 131.20 | 4.96 | 244.22 | 8.54 |
|  |  |  |  |  |  |  |
| **Grand Mean** | 41.20 | 4.22 | 128.57 | 5.36 | 239.66 | 6.52 |

**3.5 Calculation of Precision**

To evaluate precision, low, medium and high check standards were analyzed over five separate runs. Each run was carried out on a different day, with a new calibration curve. Each check standard was analyzed in triplicate within each run, giving a total of 15 replicates for each concentration. Within-run and between-run precision was calculated as CV% using a single factor ANOVA. The maximum acceptable CV% for each concentration was defined as 20%. This method was determined reproducible with precision determined as being within 3% for within-run and within 16% for between-run results.

Within-run precision was calculated for each concentration using Equation 3.2, where MS_wg_ is the mean square within groups obtained from the ANOVA table.

**Equation 3.2: Calculation of Within-Group Precision.**

$$Within-Run CV \left( \% \right)= \left[ \frac{\sqrt{{MS}_{wg}}}{Grand Mean for each concentration} \right]\times100$$

Between-run precision was calculated for each concentration using Equation 3.3, where MS_bg_ is the mean square between groups obtained from the ANOVA table and *n* is the number of observations in each group.

**Equation 3.3: Calculation of Between-Group Precision.**

$$Between-Run CV \left( \% \right)= \left[ \frac{\frac{\sqrt{{MS}_{bg}}+\left( n-1 \right) \times{MS}_{wg}}{n}}{Grand Mean for each concentration} \right]\times100$$

Table 3.6 Low check standard data for precision calculations (40 µg/mL)

| Run 1  (µg/mL) | Run 2  (µg/mL) | Run 3  (µg/mL) | Run 4  (µg/mL) | Run 5  (µg/mL) |
| --- | --- | --- | --- | --- |
| 42.02 | 38.38 | 41.43 | 41.75 | 43.34 |
| 42.34 | 39.66 | 41.04 | 42.06 | 42.71 |
| 42.01 | 39.09 | 42.09 | 43.79 | 44.49 |

Anova: Single Factor

| Groups | Count | Sum | Average | Variance |
| --- | --- | --- | --- | --- |
| Run 1 | 3.00 | 126.37 | 42.12 | 0.04 |
| Run 2 | 3.00 | 117.13 | 39.04 | 0.41 |
| Run 3 | 3.00 | 124.56 | 41.52 | 0.28 |
| Run 4 | 3.00 | 127.60 | 42.53 | 1.21 |
| Run 5 | 3.00 | 130.54 | 43.51 | 0.81 |

| **Grand Mean (µg/mL)** | 41.20 |
| --- | --- |
| **Within Run Precision** | 1.97% |
| **Between Run Precision** | 3.32% |

| Source of Variation | SS | df | MS | F | P-value | F crit |
| --- | --- | --- | --- | --- | --- | --- |
| Between Groups | 31.12 | 4.00 | 7.78 | 11.81 | 0.00 | 3.48 |
| Within Groups | 6.59 | 10.00 | 0.66 |  |  |  |
| Total | 37.71 | 14.00 |  |  |  |  |

Table 3.7 Medium check standard data for precision calculations (125 µg/mL)

| Run 1  (µg/mL) | Run 2  (µg/mL) | Run 3  (µg/mL) | Run 4  (µg/mL) | Run 5  (µg/mL) |
| --- | --- | --- | --- | --- |
| 125.63 | 117.06 | 128.40 | 130.99 | 130.52 |
| 136.07 | 115.53 | 125.67 | 137.12 | 131.82 |
| 136.04 | 118.93 | 125.93 | 137.70 | 131.20 |

Anova: Single Factor

| Groups | Count | Sum | Average | Variance |
| --- | --- | --- | --- | --- |
| Run 1 | 3.00 | 397.74 | 132.58 | 36.23 |
| Run 2 | 3.00 | 351.52 | 117.17 | 2.90 |
| Run 3 | 3.00 | 380.00 | 126.67 | 2.27 |
| Run 4 | 3.00 | 405.81 | 135.27 | 13.82 |
| Run 5 | 3.00 | 393.54 | 131.18 | 0.42 |

| **Grand Mean (µg/mL)** | 128.57 |
| --- | --- |
| **Within Run Precision** | 2.59% |
| **Between Run Precision** | 8.96% |

| Source of Variation | SS | df | MS | F | P-value | F crit |
| --- | --- | --- | --- | --- | --- | --- |
| Between Groups | 603.87 | 4.00 | 150.97 | 13.57 | 0.00 | 3.48 |
| Within Groups | 111.29 | 10.00 | 11.13 |  |  |  |
| Total | 715.15 | 14.00 |  |  |  |  |

Table 3.8 High check standard data for precision calculations (225 µg/mL)

| Run 1  (µg/mL) | Run 2  (µg/mL) | Run 3  (µg/mL) | Run 4  (µg/mL) | Run 5  (µg/mL) |
| --- | --- | --- | --- | --- |
| 239.88 | 225.96 | 234.56 | 239.28 | 252.99 |
| 224.88 | 228.02 | 232.21 | 245.91 | 251.71 |
| 243.54 | 233.74 | 250.06 | 247.98 | 244.22 |

Anova: Single Factor

| Groups | Count | Sum | Average | Variance |
| --- | --- | --- | --- | --- |
| Run 1 | 3.00 | 708.30 | 236.10 | 97.77 |
| Run 2 | 3.00 | 687.72 | 229.24 | 16.25 |
| Run 3 | 3.00 | 716.83 | 238.94 | 94.07 |
| Run 4 | 3.00 | 733.17 | 244.39 | 20.66 |
| Run 5 | 3.00 | 748.92 | 249.64 | 22.44 |

| **Grand Mean (µg/mL)** | 239.66 |
| --- | --- |
| **Within Run Precision** | 2.96% |
| **Between Run Precision** | 15.85% |

| Source of Variation | SS | df | MS | F | P-value | F crit |
| --- | --- | --- | --- | --- | --- | --- |
| Between Groups | 731.21 | 4.00 | 182.80 | 3.64 | 0.04 | 3.48 |
| Within Groups | 502.35 | 10.00 | 50.24 |  |  |  |
| Total | 1233.56 | 14.00 |  |  |  |  |

**3.6 Carryover**

To evaluate carryover, the highest methyltestosterone calibration standard (250 µg/mL) and subsequent methanol blank were analyzed over three separate runs. Each run was carried out on a different day, with a new calibration curve. This method was determined to produce no carryover.

A


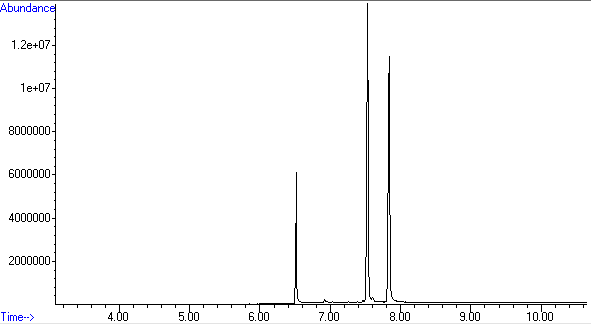

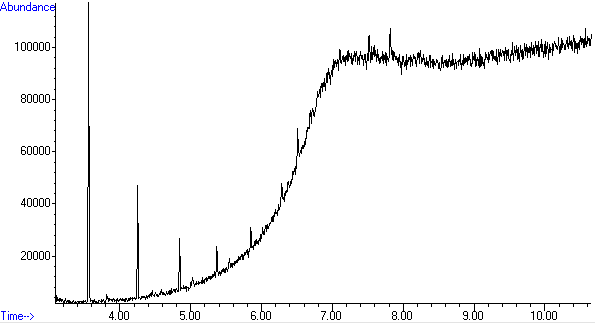


B


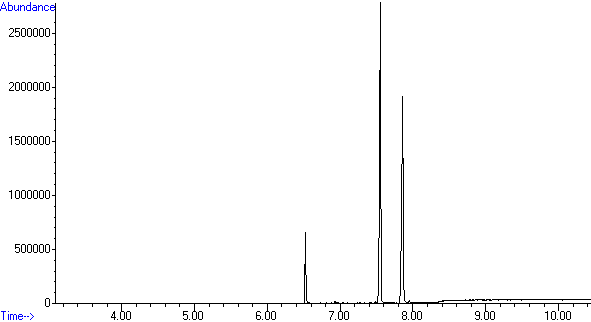

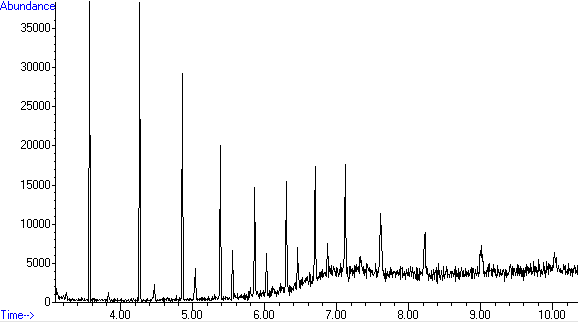


C


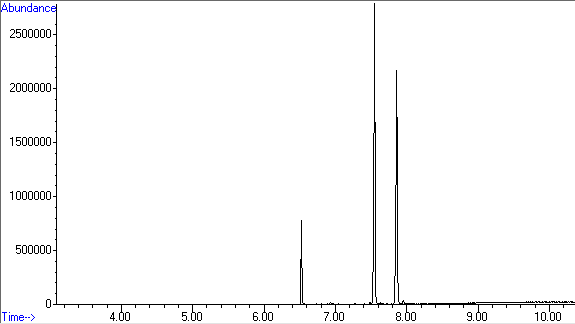

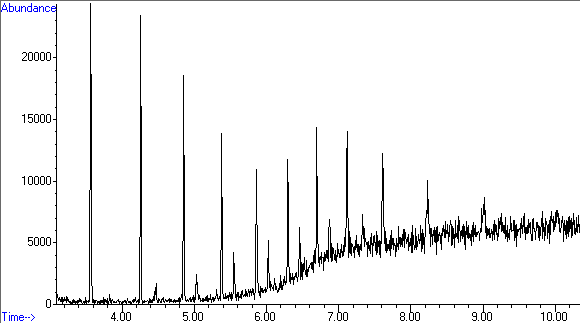


Figure S3.2 Mestanolone and methyltestosterone 250 µg/mL calibration standard (left) and subsequent methanol blank (right) run on three different days: (A) on 22-09-2023, (B) on 30-08-2023, and (C) on 29-08-2023.

**Section 4: Method validation data for metandienone quantitation method**

**4.1 Example Calibration Data**

Table S4.1 Metandienone calibration curve data for 18/10/2023

| **Concentration (µg/mL)** | **Internal Standard Peak Area** | **Metandienone Peak Area** | **Peak Area Ratio** |
| --- | --- | --- | --- |
| 25 | 18643461 | 44260495 | 0.42 |
| 50 | 38421351 | 52511134 | 0.73 |
| 75 | 62067689 | 53177887 | 1.17 |
| 100 | 86966713 | 55607701 | 1.56 |
| 150 | 126863367 | 50860212 | 2.49 |
| 200 | 174361476 | 52631100 | 3.31 |
| 250 | 198828468 | 53726873 | 3.70 |


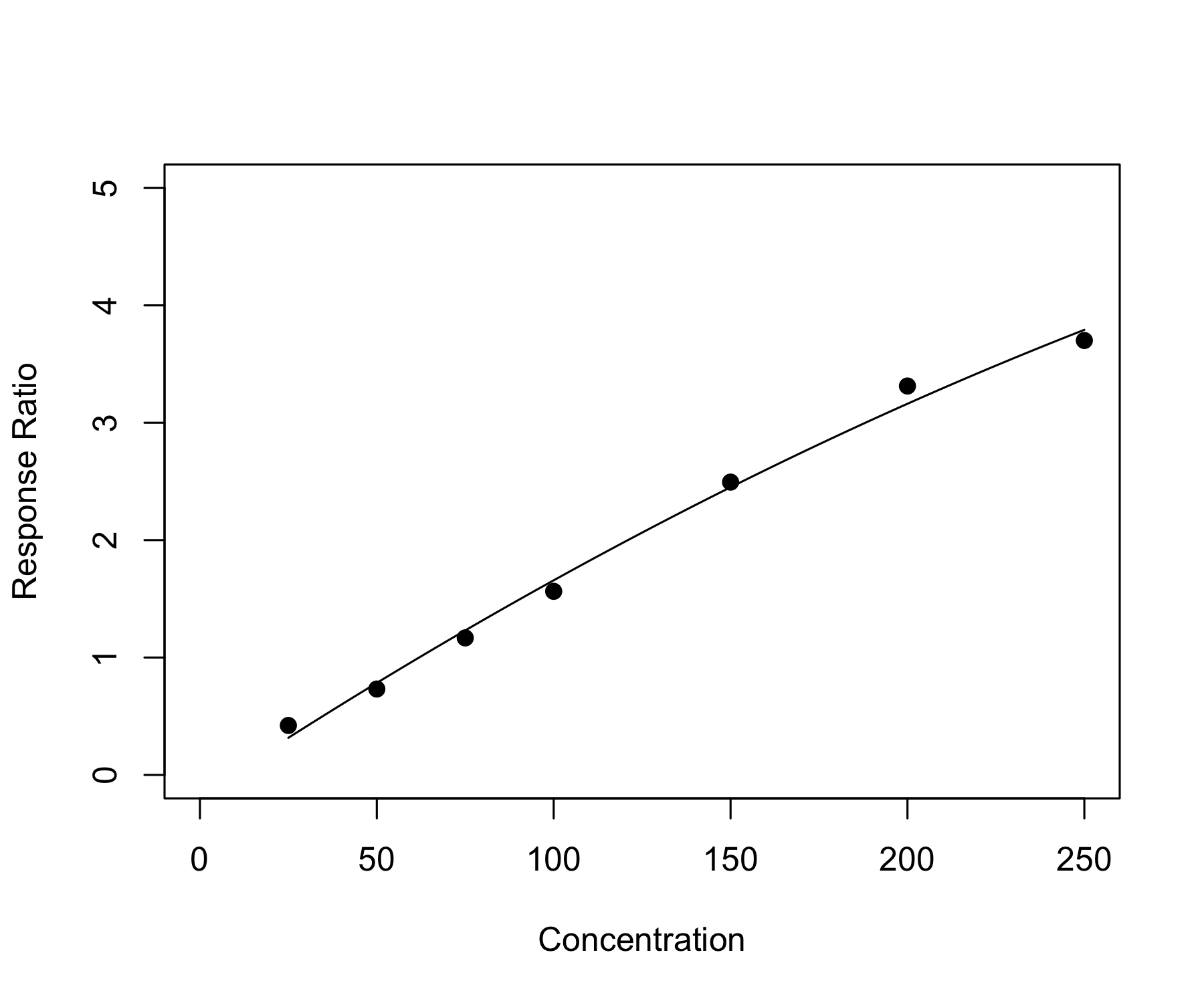


y = (-1.6×10^-5^)x^2^ + 0.020x – 0.17

R^2^ = 0.994

Figure S4.1 Metandienone calibration curve from 18/10/2023

Table S4.2 Metandienone Quality Assurance Data for 18/10/2023

| **Check Standard Concentration (µg/mL)** | **Metandienone Peak Area** | **Internal Standard Peak Area** | **Peak Area Ratio** | **Calculated Concentration (µg/mL)** | **Bias**  **(%)** |
| --- | --- | --- | --- | --- | --- |
| 40 | 26590570 | 50821344 | 0.52 | 35.94 | 10.15 |
| 40 | 23950287 | 47618809 | 0.50 | 34.86 | 12.85 |
| 125 | 98059699 | 46064323 | 2.13 | 129.09 | 3.27 |
| 125 | 104677631 | 52295719 | 2.00 | 121.07 | 3.15 |

**4.2 Limit of Detection (LOD)**

To determine the limit of detection, metandienone solutions were prepared at 5, 10, 15 and 20 μg/mL. Each solution was analyzed in replicate five times and the signal to noise ratio calculated. For each replicate, the metandienone peak was compared to the SWGDRUG mass spectral library (version 3.11, released 1 June 2022), and the (reverse) match factor was required to be greater than 850/1,000 for positive identification.

Table S4.3: Full analytical data for calculation of limit of detection

| **Concentration (µg/mL)** | **Injection** | **Max Peak Height** | **Max Noise Height** | **R Match** | **Signal to Noise Ratio** |
| --- | --- | --- | --- | --- | --- |
| 5 | 1 | 34278 | 18070 | 685 | 1.90 |
|  | 2 | 20329 | 11896 | 599 | 1.71 |
|  | 3 | 12725 | 8176 | 559 | 1.56 |
|  | 4 | - | - | - | - |
|  | 5 | - | - | - | - |
| 10 | 1 | 66977 | 18839 | 827 | 3.56 |
|  | 2 | 39849 | 12139 | 796 | 3.28 |
|  | 3 | 23261 | 6673 | 749 | 3.49 |
|  | 4 | 16448 | 4890 | 690 | 3.36 |
|  | 5 | 11719 | 3385 | 697 | 3.46 |
| 15 | 1 | 106059 | 18195 | 883 | 5.83 |
|  | 2 | 63192 | 11508 | 861 | 5.49 |
|  | 3 | 34307 | 6751 | 792 | 5.08 |
|  | 4 | 24514 | 4489 | 780 | 5.46 |
|  | 5 | 21273 | 3123 | 740 | 6.81 |
| 20 | 1 | 130518 | 18276 | 877 | 7.14 |
|  | 2 | 75594 | 11141 | 865 | 6.79 |
|  | 3 | 47718 | 6409 | 844 | 7.45 |
|  | 4 | 33585 | 4709 | 822 | 7.13 |
|  | 5 | 26054 | 3481 | 776 | 7.48 |

Evaluation of the results demonstrated an acceptable signal to noise ratio above 10 μg/mL (>3.3), however these replicates did not meet the requirements specified for positive identification of metandienone. Therefore, the LOD for this method was determined to be 20 μg/mL.

**4.3 Lower Limit of Quantitation (LLOQ)**

LLOQ was defined using the lowest non-zero calibrator. The 25 μg/mL calibration standard was analyzed across 5 separate runs and the bias was evaluated. As defined in Standard 036 (25), to be accepted, the bias of these replicates was required to fall below 20%.

Table S4.4 Analytical data for calculation of lower limit of quantitation.

| **Nominal Concentration (µg/mL)** | **Calculated Concentration (µg/mL)** |
| --- | --- |
| 25 | 29.18 |
| 25 | 30.29 |
| 25 | 28.62 |
| 25 | 30.26 |
| 25 | 30.03 |
|  |  |
| **Grand Mean** | 29.68 |
| **Bias** | 18.71% |

**Equation 4.1: Calculation of Bias (%)**

$$Bias \left( \% \right)=\left\{ \frac{Grand Mean of Calculated Concentration-Nominal Concentration}{Nominal Concentration} \right\} \times100$$

The LLOQ was defined as the lowest non-zero calibrator at 25 μg/mL with bias within 18.71% of the nominal concentration.

| **4.4 Calculation of Bias** |  |  |
| --- | --- | --- |

To evaluate bias, low, medium and high check standards for metandienone were analyzed over five separate runs. Each run was carried out on a different day, with a new calibration curve. Each check standard was analyzed in triplicate within each run, giving a total of 15 replicates for each concentration. The maximum acceptable bias for each concentration was defined as ±20%. This method was determined to be accurate with bias within ­­±13% across all check standards.

Table 4.5 Metandienone check standard data for bias calculations.

|  | **Low Check Standard (40 µg/mL)** | | **Medium Check Standard (125 µg/mL)** | | **High Check Standard (225 µg/mL)** | |
| --- | --- | --- | --- | --- | --- | --- |
|  | **Calculated Conc.** | **Bias** | **Calculated Conc.** | **Bias** | **Calculated Conc.** | **Bias** |
| **Run 1** | 34.88 | 12.81 | 129.12 | 3.30 | 228.52 | 1.56 |
|  | 35.31 | 11.73 | 131.32 | 5.05 | 216.63 | 3.72 |
|  | 35.74 | 10.64 | 114.67 | 8.26 | 221.16 | 1.70 |
| **Run 2** | 35.82 | 10.45 | 127.63 | 2.10 | 229.58 | 2.03 |
|  | 36.04 | 9.91 | 135.25 | 8.20 | 245.92 | 9.30 |
|  | 35.97 | 10.08 | 124.25 | 0.60 | 243.71 | 8.31 |
| **Run 3** | 37.73 | 5.69 | 123.15 | 1.48 | 208.42 | 7.37 |
|  | 37.24 | 6.89 | 127.43 | 1.94 | 217.62 | 3.28 |
|  | 36.40 | 9.00 | 134.08 | 7.27 | 224.71 | 0.13 |
| **Run 4** | 35.23 | 11.93 | 120.66 | 3.47 | 225.66 | 0.29 |
|  | 35.89 | 10.28 | 121.21 | 3.03 | 212.33 | 5.63 |
|  | 35.43 | 11.43 | 118.22 | 5.43 | 218.37 | 2.95 |
| **Run 5** | 35.94 | 10.15 | 129.09 | 3.27 | 220.14 | 2.16 |
|  | 34.86 | 12.85 | 121.07 | 3.15 | 224.20 | 0.35 |
|  | 35.57 | 11.08 | 124.24 | 0.60 | 233.73 | 3.88 |
|  |  |  |  |  |  |  |
| **Grand Mean** | 35.87 | 10.33 | 125.43 | 3.81 | 224.71 | 3.51 |

**4.5 Calculation of Precision**

To evaluate precision, low, medium and high check standards were analyzed over five separate runs. Each run was carried out on a different day, with a new calibration curve. Each check standard was analyzed in triplicate within each run, giving a total of 15 replicates for each concentration. Within-run and between-run precision was calculated as CV% using a single factor ANOVA. The maximum acceptable CV% for each concentration was defined as 20%. This method was determined reproducible with precision determined as being within 5% for within-run and within 19% for between-run results.

Within-run precision was calculated for each concentration using Equation 4.2, where MS_wg_ is the mean square within groups obtained from the ANOVA table.

**Equation 4.2: Calculation of Within-Group Precision.**

$$Within-Run CV \left( \% \right)= \left[ \frac{\sqrt{{MS}_{wg}}}{Grand Mean for each concentration} \right]\times100$$

Between-run precision was calculated for each concentration using Equation 4.3, where MS_bg_ is the mean square between groups obtained from the ANOVA table and *n* is the number of observations in each group.

**Equation 4.3: Calculation of Between-Group Precision.**

$$Between-Run CV \left( \% \right)= \left[ \frac{\frac{\sqrt{{MS}_{bg}}+\left( n-1 \right) \times{MS}_{wg}}{n}}{Grand Mean for each concentration} \right]\times100$$

Table 4.6 Low check standard data for precision calculations (40 µg/mL)

| Run 1  (µg/mL) | Run 2  (µg/mL) | Run 3  (µg/mL) | Run 4  (µg/mL) | Run 5  (µg/mL) |
| --- | --- | --- | --- | --- |
| 34.88 | 35.82 | 37.73 | 35.23 | 35.94 |
| 35.31 | 36.04 | 37.24 | 35.89 | 34.86 |
| 35.74 | 35.97 | 36.4 | 35.43 | 35.57 |

Anova: Single Factor

| Groups | Count | Sum | Average | Variance |
| --- | --- | --- | --- | --- |
| Run 1 | 3.00 | 105.93 | 35.31 | 0.18 |
| Run 2 | 3.00 | 107.83 | 35.94 | 0.01 |
| Run 3 | 3.00 | 111.37 | 37.12 | 0.45 |
| Run 4 | 3.00 | 106.55 | 35.52 | 0.11 |
| Run 5 | 3.00 | 106.37 | 35.46 | 0.30 |

| **Grand Mean (µg/mL)** | 35.87 |
| --- | --- |
| **Within Run Precision** | 1.28% |
| **Between Run Precision** | 3.59% |

| Source of Variation | SS | df | MS | F | P-value | F crit |
| --- | --- | --- | --- | --- | --- | --- |
| Between Groups | 6.56 | 4.00 | 1.64 | 7.69 | 0.00 | 3.48 |
| Within Groups | 2.13 | 10.00 | 0.21 |  |  |  |
| Total | 8.69 | 14.00 |  |  |  |  |

Table 4.7 Medium check standard data for precision calculations (125 µg/mL)

| Run 1  (µg/mL) | Run 2  (µg/mL) | Run 3  (µg/mL) | Run 4  (µg/mL) | Run 5  (µg/mL) |
| --- | --- | --- | --- | --- |
| 129.12 | 127.63 | 123.15 | 120.66 | 129.09 |
| 131.32 | 135.25 | 127.43 | 121.21 | 121.07 |
| 114.67 | 124.25 | 134.08 | 118.22 | 124.24 |

Anova: Single Factor

| Groups | Count | Sum | Average | Variance |
| --- | --- | --- | --- | --- |
| Run 1 | 3.00 | 375.11 | 125.04 | 81.81 |
| Run 2 | 3.00 | 387.13 | 129.04 | 31.75 |
| Run 3 | 3.00 | 384.66 | 128.22 | 30.33 |
| Run 4 | 3.00 | 360.09 | 120.03 | 2.53 |
| Run 5 | 3.00 | 374.40 | 124.80 | 16.32 |

| **Grand Mean (µg/mL)** | 125.43 |
| --- | --- |
| **Within Run Precision** | 4.55% |
| **Between Run Precision** | 18.94% |

| Source of Variation | SS | df | MS | F | P-value | F crit |
| --- | --- | --- | --- | --- | --- | --- |
| Between Groups | 151.66 | 4.00 | 37.91 | 1.16 | 0.38 | 3.48 |
| Within Groups | 325.48 | 10.00 | 32.55 |  |  |  |
| Total | 477.14 | 14.00 |  |  |  |  |

Table 4.8 High check standard data for precision calculations (225 µg/mL)

| Run 1  (µg/mL) | Run 2  (µg/mL) | Run 3  (µg/mL) | Run 4  (µg/mL) | Run 5  (µg/mL) |
| --- | --- | --- | --- | --- |
| 228.52 | 229.58 | 208.42 | 225.66 | 220.14 |
| 216.63 | 245.92 | 217.62 | 212.33 | 224.2 |
| 221.16 | 243.71 | 224.71 | 218.37 | 233.73 |

Anova: Single Factor

| Groups | Count | Sum | Average | Variance |
| --- | --- | --- | --- | --- |
| Run 1 | 3.00 | 666.31 | 222.10 | 36.01 |
| Run 2 | 3.00 | 719.21 | 239.74 | 78.59 |
| Run 3 | 3.00 | 650.75 | 216.92 | 66.71 |
| Run 4 | 3.00 | 656.36 | 218.79 | 44.55 |
| Run 5 | 3.00 | 678.07 | 226.02 | 48.67 |

| **Grand Mean (µg/mL)** | 224.71 |
| --- | --- |
| **Within Run Precision** | 3.30% |
| **Between Run Precision** | 18.62% |

| Source of Variation | SS | df | MS | F | P-value | F crit |
| --- | --- | --- | --- | --- | --- | --- |
| Between Groups | 990.43 | 4.00 | 247.61 | 4.51 | 0.02 | 3.48 |
| Within Groups | 549.06 | 10.00 | 54.91 |  |  |  |
| Total | 1539.49 | 14.00 |  |  |  |  |

**4.6 Carryover**

To evaluate carryover, the highest metandienone calibration standard (250 µg/mL) and subsequent methanol blank were analyzed over three separate runs. Each run was carried out on a different day, with a new calibration curve. This method was determined to produce no carryover.

A


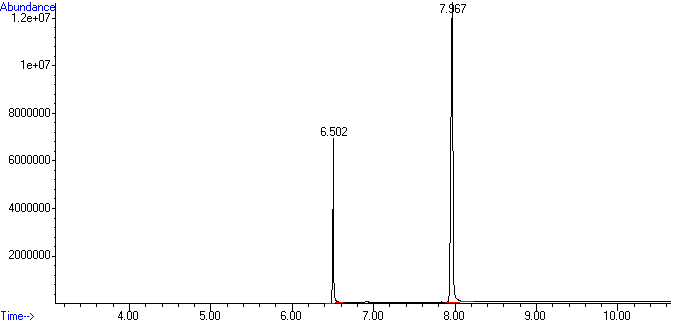

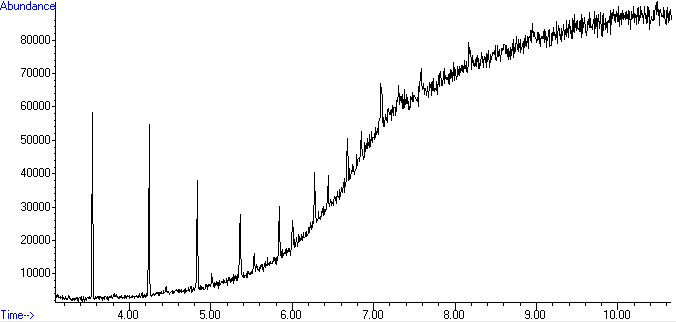


B


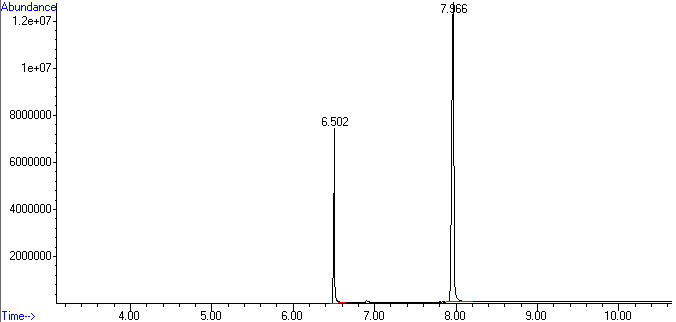

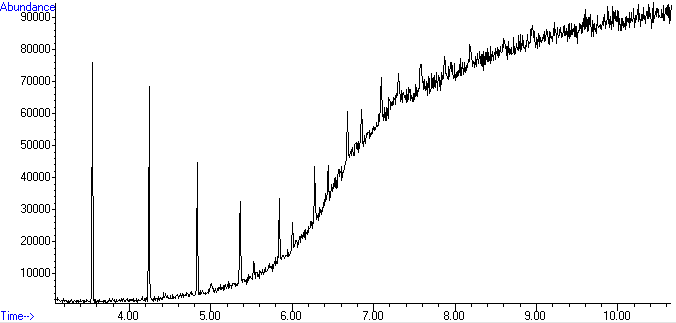


C


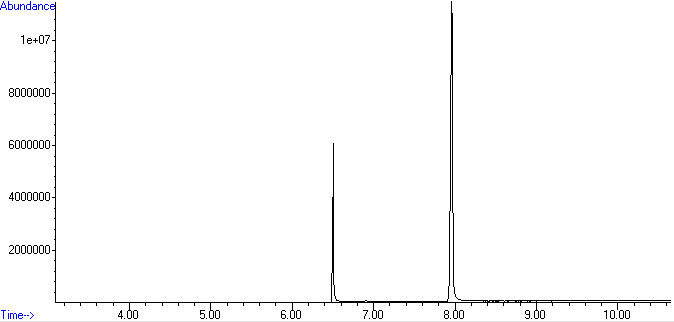

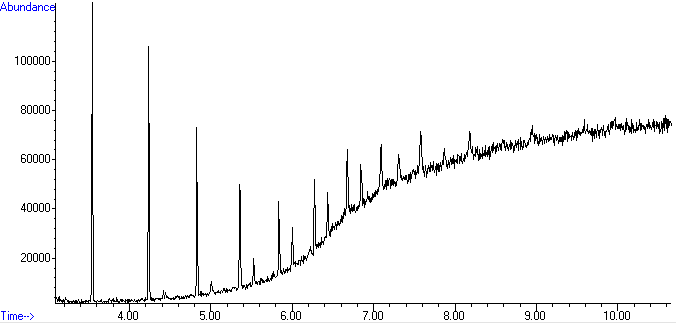


Figure S4.2 Metandienone 250 µg/mL calibration standard (left) and subsequent methanol blank (right) run on three different days: (A) on 10-10-2023, (B) on 11-10-2023, and (C) on 12-10-2023.

**Section 5: Method validation data for stanozolol quantitation method**

**5.1 Example Calibration Data**

Table S5.1 Stanozolol calibration curve data for 25/10/2023

| **Concentration (µg/mL)** | **Internal Standard Peak Area** | **Stanozolol Peak Area** | **Peak Area Ratio** |
| --- | --- | --- | --- |
| 50 | 794289 | 20210144 | 0.04 |
| 75 | 1960902 | 20188713 | 0.10 |
| 100 | 3867668 | 21659419 | 0.18 |
| 150 | 9853401 | 24216046 | 0.41 |
| 200 | 16215116 | 25063344 | 0.65 |
| 250 | 21898062 | 26968174 | 0.81 |
| 300 | 33271189 | 28060543 | 1.19 |


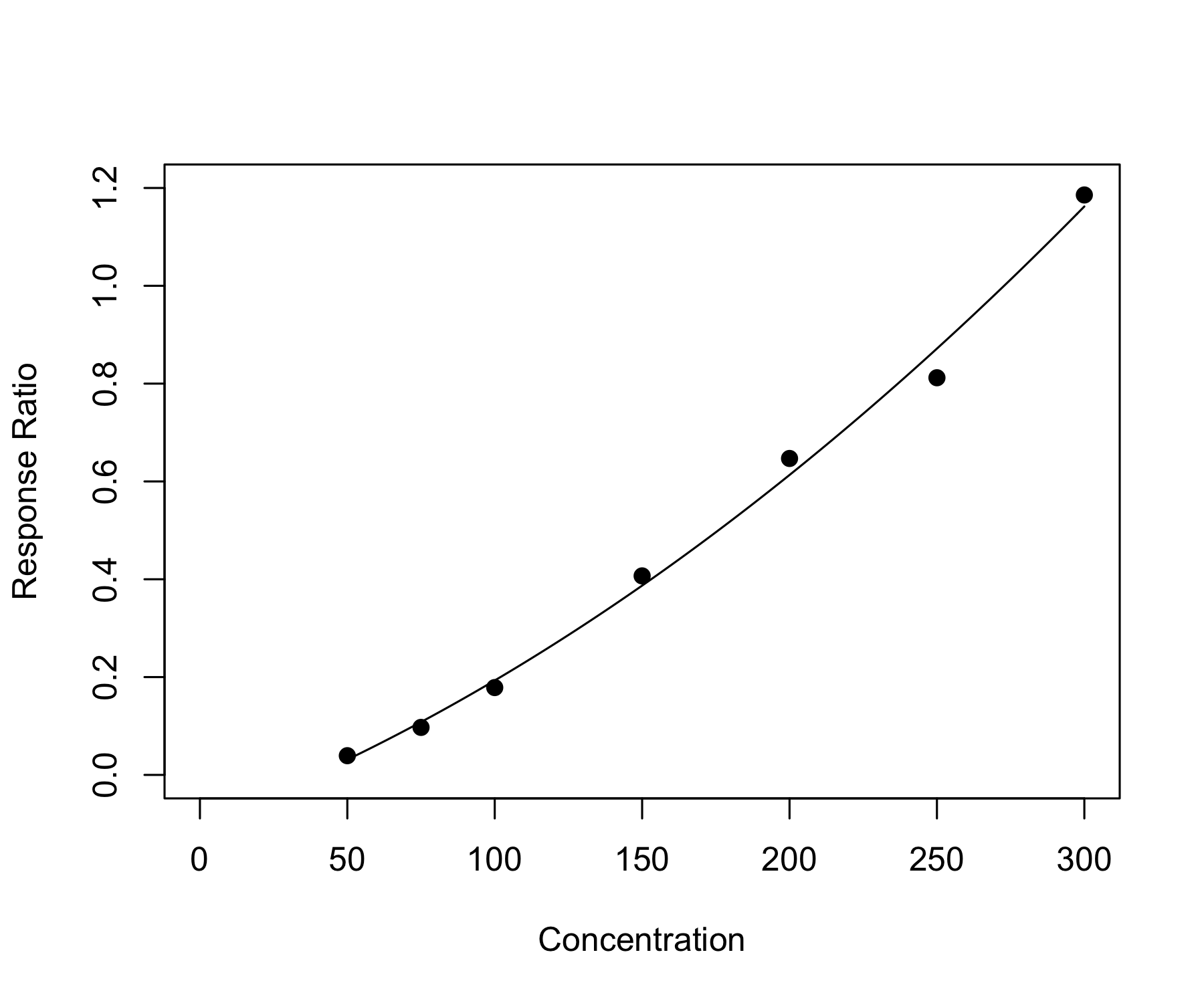


y = (6.4×10^-6^)x^2^ + 0.002x – 0.098

R^2^ = 0.994

Figure S5.1 Stanozolol calibration curve from 25/10/2023

Table S5.2 Stanozolol Quality Assurance Data for 25/10/2023

| **Check Standard Concentration (µg/mL)** | **Stanozolol Peak Area** | **Internal Standard Peak Area** | **Peak Area Ratio** | **Calculated Concentration (µg/mL)** | **Bias**  **(%)** |
| --- | --- | --- | --- | --- | --- |
| 60 | 2637042 | 28134908 | 0.09 | 70.51 | 17.51 |
| 60 | 2286534 | 27621269 | 0.08 | 67.04 | 11.73 |
| 175 | 14221603 | 26058691 | 0.55 | 185.80 | 6.17 |
| 175 | 14558008 | 26869282 | 0.54 | 184.96 | 5.69 |

**5.2 Limit of Detection (LOD)**

To determine the limit of detection, stanozolol solutions were prepared at 5, 10, 15 and 20 μg/mL. Each solution was analyzed in replicate five times and the signal to noise ratio calculated. Due to using a SIM method, each replicate of the stanozolol peak was not compared to the SWGDRUG mass spectral library as it was for the other reference standards.

Table S5.3: Full analytical data for calculation of limit of detection

| **Concentration (µg/mL)** | **Injection** | **Max Peak Height** | **Max Noise Height** | **Signal to Noise Ratio** |
| --- | --- | --- | --- | --- |
| 10 | 1 | 5765 | 5327 | 1.08 |
|  | 2 | 5227 | 4489 | 1.16 |
|  | 3 | 5025 | 4280 | 1.17 |
|  | 4 | 5011 | 4203 | 1.19 |
|  | 5 | 4968 | 4331 | 1.15 |
| 25 | 1 | 7818 | 4273 | 1.83 |
|  | 2 | 7854 | 4448 | 1.77 |
|  | 3 | 8122 | 4701 | 1.73 |
|  | 4 | 8217 | 4726 | 1.74 |
|  | 5 | 7743 | 4740 | 1.63 |
| 50 | 1 | 12058 | 2828 | 4.26 |
|  | 2 | 15990 | 2838 | 5.63 |
|  | 3 | 17793 | 2938 | 6.06 |
|  | 4 | 19591 | 3015 | 6.50 |
|  | 5 | 15601 | 4763 | 3.28 |
| 75 | 1 | 34778 | 3279 | 10.61 |
|  | 2 | 43360 | 3412 | 12.71 |
|  | 3 | 43739 | 3508 | 12.47 |
|  | 4 | 42311 | 3549 | 11.92 |
|  | 5 | 30720 | 4916 | 6.25 |

Evaluation of the results demonstrated an acceptable signal to noise ratio above 50 μg/mL (>3.3); therefore, the LOD for this method was determined to be 50 μg/mL.

**5.3 Lower Limit of Quantitation (LLOQ)**

LLOQ was defined using the lowest non-zero calibrator. The 50 μg/mL calibration standard was analyzed across 5 separate runs and the bias was evaluated. As defined in Standard 036 (25), to be accepted, the bias of these replicates was required to fall below 20%.

Table S5.4 Analytical data for calculation of lower limit of quantitation.

| **Nominal Concentration (µg/mL)** | **Calculated Concentration (µg/mL)** |
| --- | --- |
| 50 | 52.80 |
| 50 | 46.83 |
| 50 | 55.75 |
| 50 | 55.08 |
| 50 | 52.75 |
|  |  |
| **Grand Mean** | 52.64 |
| **Bias** | 5.28% |

**Equation 4.1: Calculation of Bias (%)**

$$Bias \left( \% \right)=\left\{ \frac{Grand Mean of Calculated Concentration-Nominal Concentration}{Nominal Concentration} \right\} \times100$$

The LLOQ was defined as the lowest non-zero calibrator at 50 μg/mL with bias within 5.28% of the nominal concentration.

| **5.4 Calculation of Bias** |  |  |
| --- | --- | --- |

To evaluate bias, low, medium and high check standards for stanozolol were analyzed over five separate runs. Each run was carried out on a different day, with a new calibration curve. Each check standard was analyzed in triplicate within each run, giving a total of 15 replicates for each concentration. The maximum acceptable bias for each concentration was defined as ±20%. This method was determined to be accurate with bias within ­­±18% across all check standards.

Table 5.5 Stanzolol check standard data for bias calculations.

|  | **Low Check Standard (60 µg/mL)** | | **Medium Check Standard (175 µg/mL)** | | **High Check Standard (275 µg/mL)** | |
| --- | --- | --- | --- | --- | --- | --- |
|  | **Calculated Conc.** | **Bias** | **Calculated Conc.** | **Bias** | **Calculated Conc.** | **Bias** |
| **Run 1** | 58.51 | 2.49 | 177.89 | 1.65 | 267.57 | 2.70 |
|  | 62.08 | 3.46 | 178.04 | 1.74 | 269.46 | 2.02 |
|  | 61.74 | 2.90 | 176.08 | 0.61 | 270.41 | 1.67 |
| **Run 2** | 64.44 | 7.41 | 188.89 | 7.94 | 272.44 | 0.93 |
|  | 65.08 | 8.47 | 193.11 | 10.35 | 284.29 | 3.38 |
|  | 63.39 | 5.66 | 192.30 | 9.89 | 283.70 | 3.16 |
| **Run 3** | 69.10 | 15.17 | 182.29 | 4.17 | 260.61 | 5.23 |
|  | 65.97 | 9.94 | 176.17 | 0.67 | 261.96 | 4.74 |
|  | 65.87 | 9.78 | 171.97 | 1.73 | 282.39 | 2.69 |
| **Run 4** | 67.40 | 12.33 | 179.99 | 2.85 | 287.13 | 4.41 |
|  | 65.26 | 8.77 | 190.73 | 8.99 | 279.21 | 1.53 |
|  | 65.83 | 9.71 | 183.43 | 4.82 | 273.86 | 0.42 |
| **Run 5** | 70.51 | 17.51 | 185.80 | 6.17 | 288.23 | 4.81 |
|  | 67.04 | 11.73 | 184.96 | 5.69 | 271.85 | 1.15 |
|  | 67.48 | 12.47 | 181.25 | 3.57 | 291.91 | 6.15 |
|  |  |  |  |  |  |  |
| **Grand Mean** | 65.31 | 9.19 | 182.86 | 4.72 | 276.33 | 3.00 |

**5.5 Calculation of Precision**

To evaluate precision, low, medium and high check standards were analyzed over five separate runs. Each run was carried out on a different day, with a new calibration curve. Each check standard was analyzed in triplicate within each run, giving a total of 15 replicates for each concentration. Within-run and between-run precision was calculated as CV% using a single factor ANOVA. The maximum acceptable CV% for each concentration was defined as 20%. This method was determined reproducible with precision determined as being within 4% for within-run and within 20% for between-run results.

Within-run precision was calculated for each concentration using Equation 5.2, where MS_wg_ is the mean square within groups obtained from the ANOVA table.

**Equation 5.2: Calculation of Within-Group Precision.**

$$Within-Run CV \left( \% \right)= \left[ \frac{\sqrt{{MS}_{wg}}}{Grand Mean for each concentration} \right]\times100$$

Between-run precision was calculated for each concentration using Equation 5.3, where MS_bg_ is the mean square between groups obtained from the ANOVA table and *n* is the number of observations in each group.

**Equation 5.3: Calculation of Between-Group Precision.**

$$Between-Run CV \left( \% \right)= \left[ \frac{\frac{\sqrt{{MS}_{bg}}+\left( n-1 \right) \times{MS}_{wg}}{n}}{Grand Mean for each concentration} \right]\times100$$

Table 4.6 Low check standard data for precision calculations (40 µg/mL)

| Run 1  (µg/mL) | Run 2  (µg/mL) | Run 3  (µg/mL) | Run 4  (µg/mL) | Run 5  (µg/mL) |
| --- | --- | --- | --- | --- |
| 58.51 | 64.44 | 69.10 | 67.40 | 70.51 |
| 62.08 | 65.08 | 65.97 | 65.26 | 67.04 |
| 61.74 | 63.39 | 65.87 | 65.83 | 67.48 |

Anova: Single Factor

| Groups | Count | Sum | Average | Variance |
| --- | --- | --- | --- | --- |
| Run 1 | 3.00 | 182.33 | 60.78 | 3.88 |
| Run 2 | 3.00 | 192.91 | 64.30 | 0.73 |
| Run 3 | 3.00 | 200.94 | 66.98 | 3.37 |
| Run 4 | 3.00 | 198.49 | 66.16 | 1.23 |
| Run 5 | 3.00 | 205.03 | 68.34 | 3.57 |

| **Grand Mean (µg/mL)** | 65.31 |
| --- | --- |
| **Within Run Precision** | 2.45% |
| **Between Run Precision** | 5.20% |

| Source of Variation | SS | df | MS | F | P-value | F crit |
| --- | --- | --- | --- | --- | --- | --- |
| Between Groups | 102.85 | 4.00 | 25.71 | 10.06 | 0.00 | 3.48 |
| Within Groups | 25.56 | 10.00 | 2.56 |  |  |  |
| Total | 128.41 | 14.00 |  |  |  |  |

Table 4.7 Medium check standard data for precision calculations (125 µg/mL)

| Run 1  (µg/mL) | Run 2  (µg/mL) | Run 3  (µg/mL) | Run 4  (µg/mL) | Run 5  (µg/mL) |
| --- | --- | --- | --- | --- |
| 177.89 | 188.89 | 182.29 | 179.99 | 185.80 |
| 178.04 | 193.11 | 176.17 | 190.73 | 184.96 |
| 176.08 | 192.30 | 171.97 | 183.43 | 181.25 |

Anova: Single Factor

| Groups | Count | Sum | Average | Variance |
| --- | --- | --- | --- | --- |
| Run 1 | 3.00 | 532.01 | 177.34 | 1.19 |
| Run 2 | 3.00 | 574.30 | 191.43 | 5.02 |
| Run 3 | 3.00 | 530.43 | 176.81 | 26.93 |
| Run 4 | 3.00 | 554.15 | 184.72 | 30.08 |
| Run 5 | 3.00 | 552.01 | 184.00 | 5.86 |

| **Grand Mean (µg/mL)** | 182.86 |
| --- | --- |
| **Within Run Precision** | 2.03% |
| **Between Run Precision** | 6.94% |

| Source of Variation | SS | df | MS | F | P-value | F crit |
| --- | --- | --- | --- | --- | --- | --- |
| Between Groups | 436.10 | 4.00 | 109.02 | 7.89 | 0.00z | 3.48 |
| Within Groups | 138.16 | 10.00 | 13.82 |  |  |  |
| Total | 574.26 | 14.00 |  |  |  |  |

Table 4.8 High check standard data for precision calculations (225 µg/mL)

| Run 1  (µg/mL) | Run 2  (µg/mL) | Run 3  (µg/mL) | Run 4  (µg/mL) | Run 5  (µg/mL) |
| --- | --- | --- | --- | --- |
| 267.57 | 272.44 | 260.61 | 287.13 | 288.23 |
| 269.46 | 284.29 | 261.96 | 279.21 | 271.85 |
| 270.41 | 283.7 | 282.39 | 273.86 | 291.91 |

Anova: Single Factor

| Groups | Count | Sum | Average | Variance |
| --- | --- | --- | --- | --- |
| Run 1 | 3.00 | 807.44 | 269.15 | 2.09 |
| Run 2 | 3.00 | 840.43 | 280.14 | 44.59 |
| Run 3 | 3.00 | 804.96 | 268.32 | 148.93 |
| Run 4 | 3.00 | 832.84 | 277.61 | 68.95 |
| Run 5 | 3.00 | 851.99 | 284.00 | 114.04 |

| **Grand Mean (µg/mL)** | 276.33 |
| --- | --- |
| **Within Run Precision** | 3.15% |
| **Between Run Precision** | 19.71% |

| Source of Variation | SS | df | MS | F | P-value | F crit |
| --- | --- | --- | --- | --- | --- | --- |
| Between Groups | 583.97 | 4.00 | 145.99 | 1.29 | 0.34 | 3.48 |
| Within Groups | 1135.94 | 10.00 | 113.59 |  |  |  |
| Total | 1719.90 | 14.00 |  |  |  |  |

**4.6 Carryover**

To evaluate carryover, the highest stanozolol calibration standard (300 µg/mL) and subsequent methanol blank were analyzed over three separate runs. Each run was carried out on a different day, with a new calibration curve. This method was determined to produce some carryover, so a methanol blank.

A


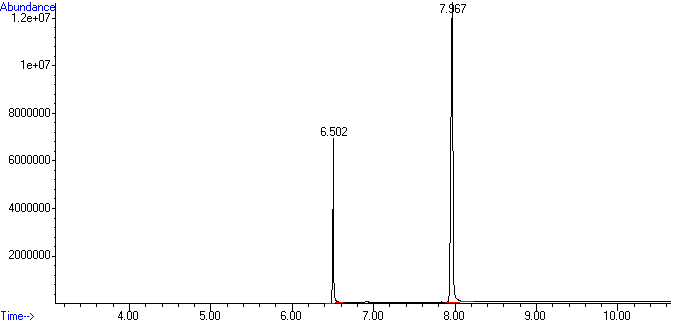

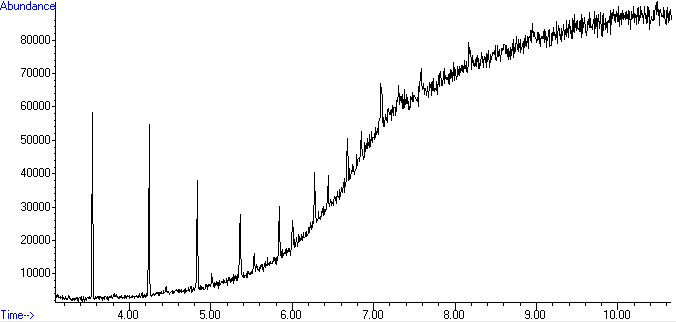


B


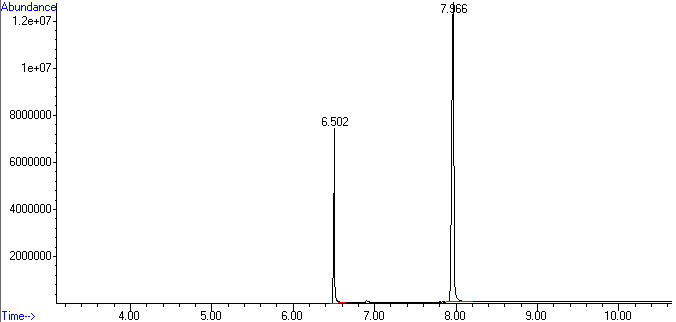

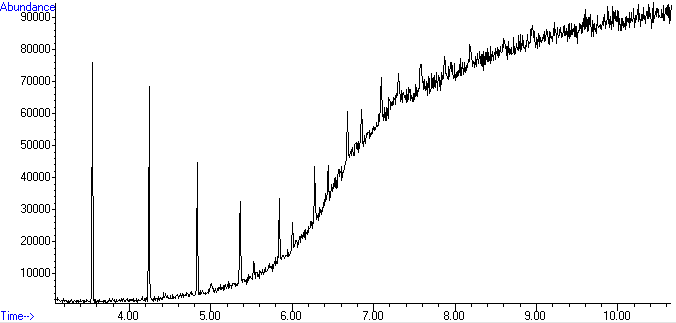


C


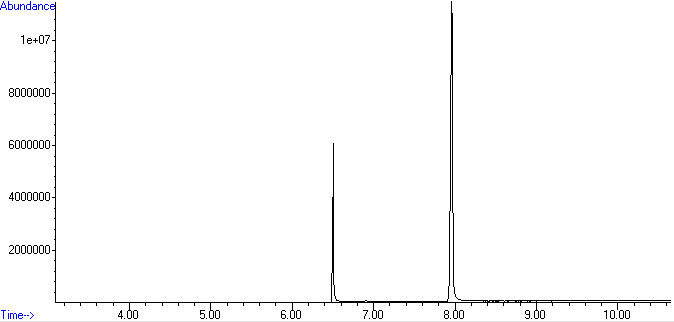

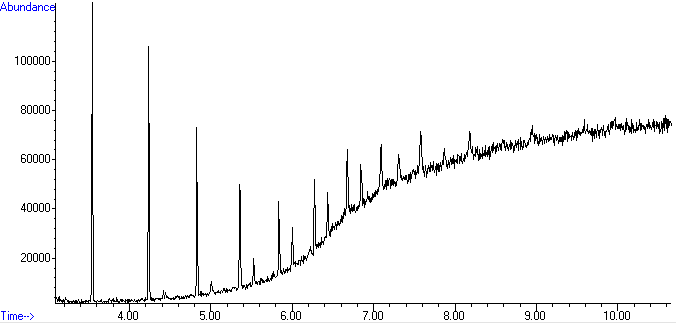


Figure S4.2 Metandienone 250 µg/mL calibration standard (left) and subsequent methanol blank (right) run on three different days: (A) on 10-10-2023, (B) on 11-10-2023, and (C) on 12-10-2023.

**Section 6: Method validation data for oxymetholone quantitation method**

**6.1 Example Calibration Data**

Table S6.1 Oxymetholone calibration curve data for 07/11/2023

| **Concentration (µg/mL)** | **Internal Standard Peak Area** | **Oxymetholone Peak Area** | **Peak Area Ratio** |
| --- | --- | --- | --- |
| 50 | 4161793 | 36550670 | 0.11 |
| 75 | 13083537 | 39483453 | 0.33 |
| 100 | 22980584 | 39342463 | 0.58 |
| 150 | 47308437 | 40876844 | 1.16 |
| 175 | 63057299 | 42575114 | 1.48 |
| 200 | 81891155 | 45102542 | 1.82 |
| 250 | 110933116 | 45636442 | 2.43 |


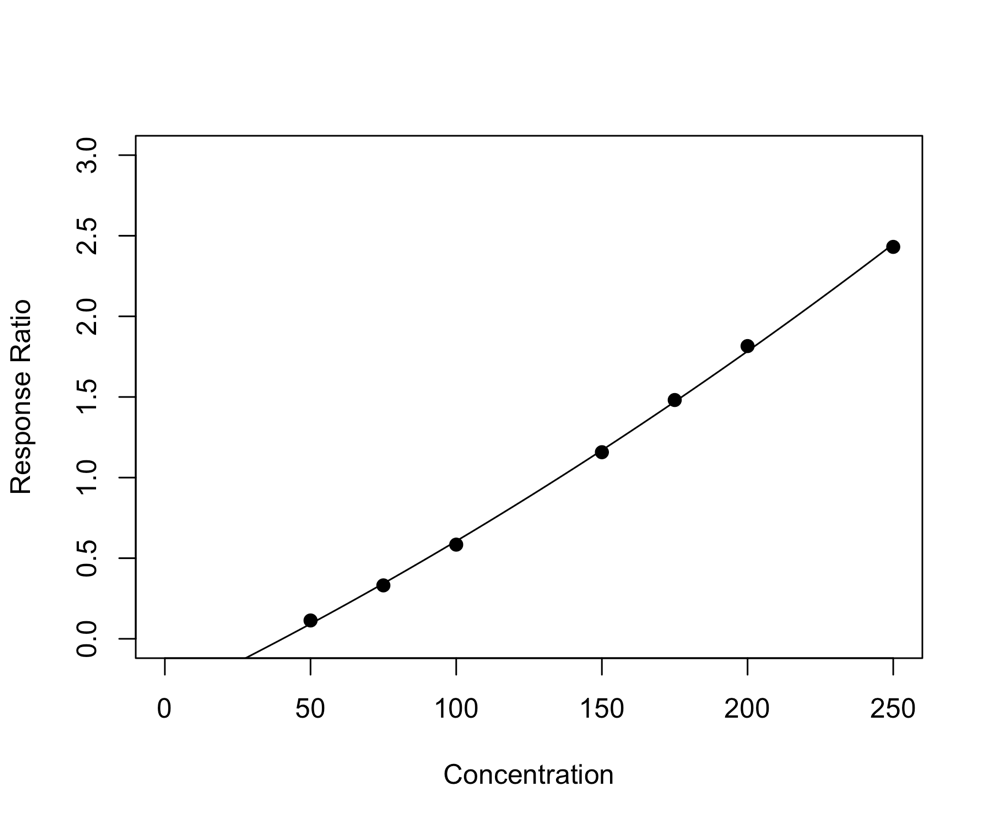


y = (1.0×10^-5^)x^2^ + 0.009x – 0.37

R^2^ = 0.999

Figure S6.1 Oxymetholone calibration curve from 07/11/2023

Table S6.2 Oxymetholone Quality Assurance Data for 07/11/2023

| **Check Standard Concentration (µg/mL)** | **Oxymetholone Peak Area** | **Internal Standard Peak Area** | **Peak Area Ratio** | **Calculated Concentration (µg/mL)** | **Bias**  **(%)** |
| --- | --- | --- | --- | --- | --- |
| 60 | 11957041 | 44018618 | 0.27 | 67.98 | 13.30 |
| 60 | 10792128 | 42902914 | 0.25 | 65.99 | 9.99 |
| 125 | 36211304 | 41485237 | 0.87 | 124.23 | 0.62 |
| 125 | 42988088 | 46221993 | 0.93 | 129.28 | 3.43 |

**6.2 Limit of Detection (LOD)**

To determine the limit of detection, oxymetholone solutions were prepared at 5, 10, 15 and 20 μg/mL. Each solution was analyzed in replicate five times and the signal to noise ratio calculated. For each replicate, the oxymetholone peak was compared to the SWGDRUG mass spectral library (version 3.11, released 1 June 2022), and the (reverse) match factor was required to be greater than 850/1,000 for positive identification.

Table S6.3: Full analytical data for calculation of limit of detection

| **Concentration (µg/mL)** | **Injection** | **Max Peak Height** | **Max Noise Height** | **R Match** | **Signal to Noise Ratio** |
| --- | --- | --- | --- | --- | --- |
| 20 | 1 | 68314 | 54931 | 561 | 1.24 |
|  | 2 | 93188 | 59987 | 637 | 1.55 |
|  | 3 | 105689 | 69104 | 641 | 1.53 |
|  | 4 | 112802 | 77294 | 648 | 1.46 |
|  | 5 | 118160 | 80219 | 648 | 1.47 |
| 50 | 1 | 357212 | 71348 | 871 | 5.01 |
|  | 2 | 398710 | 81026 | 877 | 4.92 |
|  | 3 | 384904 | 94247 | 867 | 4.08 |
|  | 4 | 457721 | 97794 | 902 | 4.68 |
|  | 5 | 397428 | 103621 | 873 | 3.84 |
| 100 | 1 | 1447655 | 137944 | 925 | 10.49 |
|  | 2 | 1708406 | 143890 | 935 | 11.87 |
|  | 3 | 1691457 | 158974 | 930 | 10.64 |
|  | 4 | 1698003 | 150386 | 938 | 11.29 |
|  | 5 | 1813182 | 155348 | 938 | 11.67 |
| 150 | 1 | 2873325 | 166561 | 943 | 17.25 |
|  | 2 | 3340950 | 181344 | 946 | 18.42 |
|  | 3 | 3459972 | 191806 | 945 | 18.04 |
|  | 4 | 3366542 | 193002 | 941 | 17.44 |
|  | 5 | 3549280 | 191929 | 950 | 18.49 |

Evaluation of the results demonstrated an acceptable signal to noise ratio above 50 μg/mL (>3.3) and these replicates met the requirements specified for positive identification of oxymetholone. Therefore, the LOD for this method was determined to be 50 μg/mL.

**6.3 Lower Limit of Quantitation (LLOQ)**

LLOQ was defined using the lowest non-zero calibrator. The 50 μg/mL calibration standard was analyzed across 5 separate runs and the bias was evaluated. As defined in Standard 036 (25), to be accepted, the bias of these replicates was required to fall below 20%.

Table S6.4 Analytical data for calculation of lower limit of quantitation.

| **Nominal Concentration (µg/mL)** | **Calculated Concentration (µg/mL)** |
| --- | --- |
| 50 | 51.11 |
| 50 | 54.56 |
| 50 | 50.76 |
| 50 | 52.49 |
| 50 | 52.14 |
|  |  |
| **Grand Mean** | 52.21 |
| **Bias** | 4.42% |

**Equation 4.1: Calculation of Bias (%)**

$$Bias \left( \% \right)=\left\{ \frac{Grand Mean of Calculated Concentration-Nominal Concentration}{Nominal Concentration} \right\} \times100$$

The LLOQ was defined as the lowest non-zero calibrator at 50 μg/mL with bias within 4.42% of the nominal concentration.

| **6.4 Calculation of Bias** |  |  |
| --- | --- | --- |

To evaluate bias, low, medium and high check standards for oxymetholone were analyzed over five separate runs. Each run was carried out on a different day, with a new calibration curve. Each check standard was analyzed in triplicate within each run, giving a total of 15 replicates for each concentration. The maximum acceptable bias for each concentration was defined as ±20%. This method was determined to be accurate with bias within ­­±19% across all check standards.

Table 6.5 Oxymetholone check standard data for bias calculations.

|  | **Low Check Standard (60 µg/mL)** | | **Medium Check Standard (125 µg/mL)** | | **High Check Standard (225 µg/mL)** | |
| --- | --- | --- | --- | --- | --- | --- |
|  | **Calculated Conc.** | **Bias** | **Calculated Conc.** | **Bias** | **Calculated Conc.** | **Bias** |
| **Run 1** | 67.65 | 12.75 | 128.09 | 2.47 | 222.26 | 1.22 |
|  | 69.78 | 16.30 | 128.04 | 2.44 | 226.67 | 0.74 |
|  | 70.99 | 18.32 | 136.84 | 9.47 | 228.70 | 1.65 |
| **Run 2** | 66.41 | 10.68 | 113.27 | 9.38 | 225.86 | 0.38 |
|  | 69.06 | 15.09 | 129.78 | 3.82 | 234.50 | 4.22 |
|  | 69.41 | 15.68 | 122.25 | 2.20 | 238.45 | 5.98 |
| **Run 3** | 66.58 | 10.96 | 126.47 | 1.17 | 213.39 | 5.16 |
|  | 68.95 | 14.92 | 132.46 | 5.97 | 222.19 | 1.25 |
|  | 68.62 | 14.37 | 131.09 | 4.87 | 220.52 | 1.99 |
| **Run 4** | 66.93 | 11.56 | 120.33 | 3.73 | 217.52 | 3.33 |
|  | 69.40 | 15.66 | 129.19 | 3.35 | 227.32 | 1.03 |
|  | 71.27 | 18.78 | 126.34 | 1.07 | 222.47 | 1.12 |
| **Run 5** | 67.98 | 13.30 | 124.23 | 0.62 | 227.96 | 1.32 |
|  | 65.99 | 9.99 | 129.28 | 3.43 | 233.92 | 3.96 |
|  | 65.46 | 9.10 | 127.78 | 2.22 | 236.68 | 5.19 |
|  |  |  |  |  |  |  |
| **Grand Mean** | 68.30 | 13.83 | 127.03 | 3.75 | 226.56 | 2.57 |

**6.5 Calculation of Precision**

To evaluate precision, low, medium and high check standards were analyzed over five separate runs. Each run was carried out on a different day, with a new calibration curve. Each check standard was analyzed in triplicate within each run, giving a total of 15 replicates for each concentration. Within-run and between-run precision was calculated as CV% using a single factor ANOVA. The maximum acceptable CV% for each concentration was defined as 20%. This method was determined reproducible with precision determined as being within 5% for within-run and within 16% for between-run results.

Within-run precision was calculated for each concentration using Equation 6.2, where MS_wg_ is the mean square within groups obtained from the ANOVA table.

**Equation 6.2: Calculation of Within-Group Precision.**

$$Within-Run CV \left( \% \right)= \left[ \frac{\sqrt{{MS}_{wg}}}{Grand Mean for each concentration} \right]\times100$$

Between-run precision was calculated for each concentration using Equation 6.3, where MS_bg_ is the mean square between groups obtained from the ANOVA table and *n* is the number of observations in each group.

**Equation 6.3: Calculation of Between-Group Precision.**

$$Between-Run CV \left( \% \right)= \left[ \frac{\frac{\sqrt{{MS}_{bg}}+\left( n-1 \right) \times{MS}_{wg}}{n}}{Grand Mean for each concentration} \right]\times100$$

Table 6.6 Low check standard data for precision calculations (60 µg/mL)

| Run 1  (µg/mL) | Run 2  (µg/mL) | Run 3  (µg/mL) | Run 4  (µg/mL) | Run 5  (µg/mL) |
| --- | --- | --- | --- | --- |
| 67.65 | 66.41 | 66.58 | 66.93 | 67.98 |
| 69.78 | 69.06 | 68.95 | 69.4 | 65.99 |
| 70.99 | 69.41 | 68.62 | 71.27 | 65.46 |

Anova: Single Factor

| Groups | Count | Sum | Average | Variance |
| --- | --- | --- | --- | --- |
| Run 1 | 3.00 | 208.42 | 69.47 | 2.86 |
| Run 2 | 3.00 | 204.88 | 68.29 | 2.69 |
| Run 3 | 3.00 | 204.15 | 68.05 | 1.65 |
| Run 4 | 3.00 | 207.60 | 69.20 | 4.74 |
| Run 5 | 3.00 | 199.43 | 66.48 | 1.77 |

| **Grand Mean (µg/mL)** | 68.30 |
| --- | --- |
| **Within Run Precision** | 2.42% |
| **Between Run Precision** | 3.67% |

| Source of Variation | SS | df | MS | F | P-value | F crit |
| --- | --- | --- | --- | --- | --- | --- |
| Between Groups | 16.72 | 4.00 | 4.18 | 1.53 | 0.27 | 3.48 |
| Within Groups | 27.40 | 10.00 | 2.74 |  |  |  |
| Total | 44.13 | 14.00 |  |  |  |  |

Table 6.7 Medium check standard data for precision calculations (125 µg/mL)

| Run 1  (µg/mL) | Run 2  (µg/mL) | Run 3  (µg/mL) | Run 4  (µg/mL) | Run 5  (µg/mL) |
| --- | --- | --- | --- | --- |
| 128.09 | 113.27 | 126.47 | 120.33 | 124.23 |
| 128.04 | 129.78 | 132.46 | 129.19 | 129.28 |
| 136.84 | 122.25 | 131.09 | 126.34 | 127.78 |

Anova: Single Factor

| Groups | Count | Sum | Average | Variance |
| --- | --- | --- | --- | --- |
| Run 1 | 3.00 | 392.97 | 130.99 | 25.67 |
| Run 2 | 3.00 | 365.30 | 121.77 | 68.32 |
| Run 3 | 3.00 | 390.02 | 130.01 | 9.85 |
| Run 4 | 3.00 | 375.86 | 125.29 | 20.46 |
| Run 5 | 3.00 | 381.29 | 127.10 | 6.73 |

| **Grand Mean (µg/mL)** | 127.03 |
| --- | --- |
| **Within Run Precision** | 4.03% |
| **Between Run Precision** | 15.44% |

| Source of Variation | SS | df | MS | F | P-value | F crit |
| --- | --- | --- | --- | --- | --- | --- |
| Between Groups | 165.87 | 4.00 | 41.47 | 1.58 | 0.25 | 3.48 |
| Within Groups | 262.04 | 10.00 | 26.20 |  |  |  |
| Total | 427.91 | 14.00 |  |  |  |  |

Table 6.8 High check standard data for precision calculations (225 µg/mL)

| Run 1  (µg/mL) | Run 2  (µg/mL) | Run 3  (µg/mL) | Run 4  (µg/mL) | Run 5  (µg/mL) |
| --- | --- | --- | --- | --- |
| 222.26 | 225.86 | 213.39 | 217.52 | 227.96 |
| 226.67 | 234.5 | 222.19 | 227.32 | 233.92 |
| 228.7 | 238.45 | 220.52 | 222.47 | 236.68 |

Anova: Single Factor

| Groups | Count | Sum | Average | Variance |
| --- | --- | --- | --- | --- |
| Run 1 | 3.00 | 677.63 | 225.88 | 10.84 |
| Run 2 | 3.00 | 698.81 | 232.94 | 41.46 |
| Run 3 | 3.00 | 656.10 | 218.70 | 21.84 |
| Run 4 | 3.00 | 667.31 | 222.44 | 24.01 |
| Run 5 | 3.00 | 698.56 | 232.85 | 19.86 |

| **Grand Mean (µg/mL)** | 226.56 |
| --- | --- |
| **Within Run Precision** | 2.14% |
| **Between Run Precision** | 8.55% |

| Source of Variation | SS | df | MS | F | P-value | F crit |
| --- | --- | --- | --- | --- | --- | --- |
| Between Groups | 478.55 | 4.00 | 119.64 | 5.07 | 0.02 | 3.48 |
| Within Groups | 236.04 | 10.00 | 23.60 |  |  |  |
| Total | 714.59 | 14.00 |  |  |  |  |

**6.6 Carryover**

To evaluate carryover, the highest oxymetholone calibration standard (250 µg/mL) and subsequent methanol blank were analyzed over three separate runs. Each run was carried out on a different day, with a new calibration curve. This method was determined to produce no carryover.

A


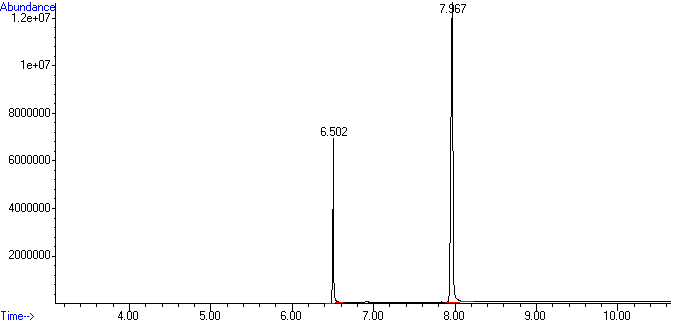

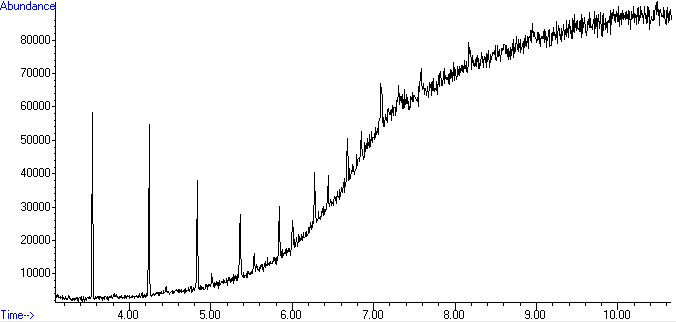


B


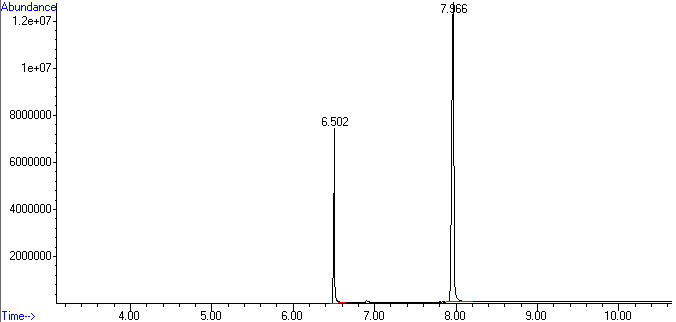

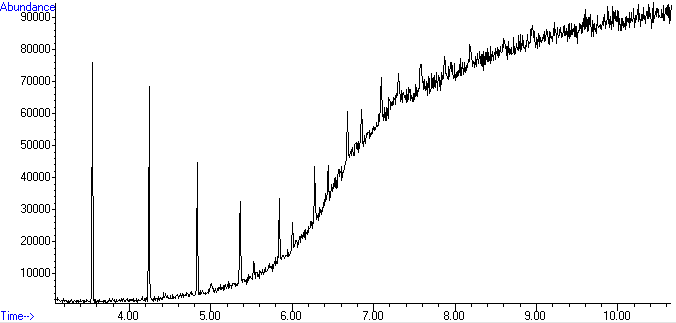


C


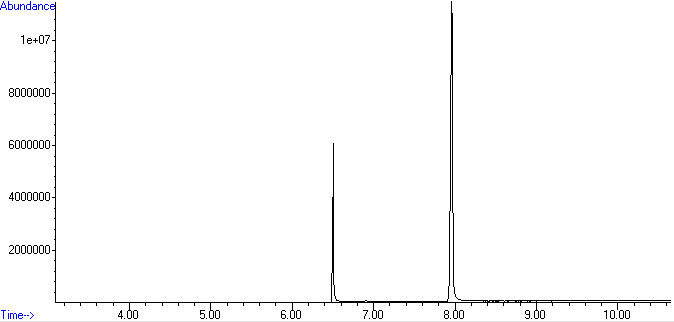

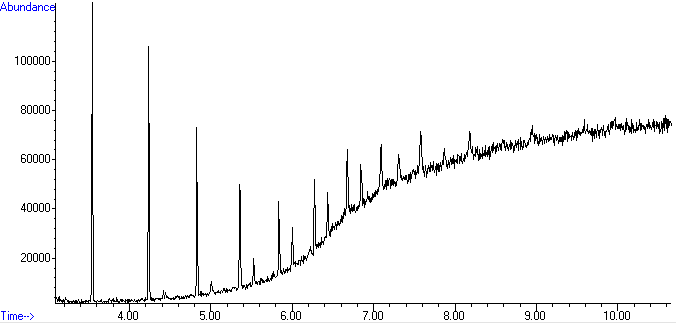


Figure S6.2 Oxymetholone 250 µg/mL calibration standard (left) and subsequent methanol blank (right) run on three different days: (A) on 26-10-2023, (B) on 27-10-2023, and (C) on 31-10-2023.

**Section 7: Method validation data for oxandrolone quantitation method**

**7.1 Example Calibration Data**

Table S7.1 Oxandrolone calibration curve data for 10/11/2023

| **Concentration (µg/mL)** | **Internal Standard Peak Area** | **Oxandrolone Peak Area** | **Peak Area Ratio** |
| --- | --- | --- | --- |
| 25 | 1297696 | 3515473 | 0.37 |
| 50 | 3083593 | 3658858 | 0.84 |
| 75 | 4737125 | 3507270 | 1.35 |
| 100 | 6363221 | 3557872 | 1.79 |
| 150 | 9905552 | 3695302 | 2.68 |
| 200 | 12812244 | 3447088 | 3.72 |
| 250 | 17091712 | 3430814 | 4.98 |


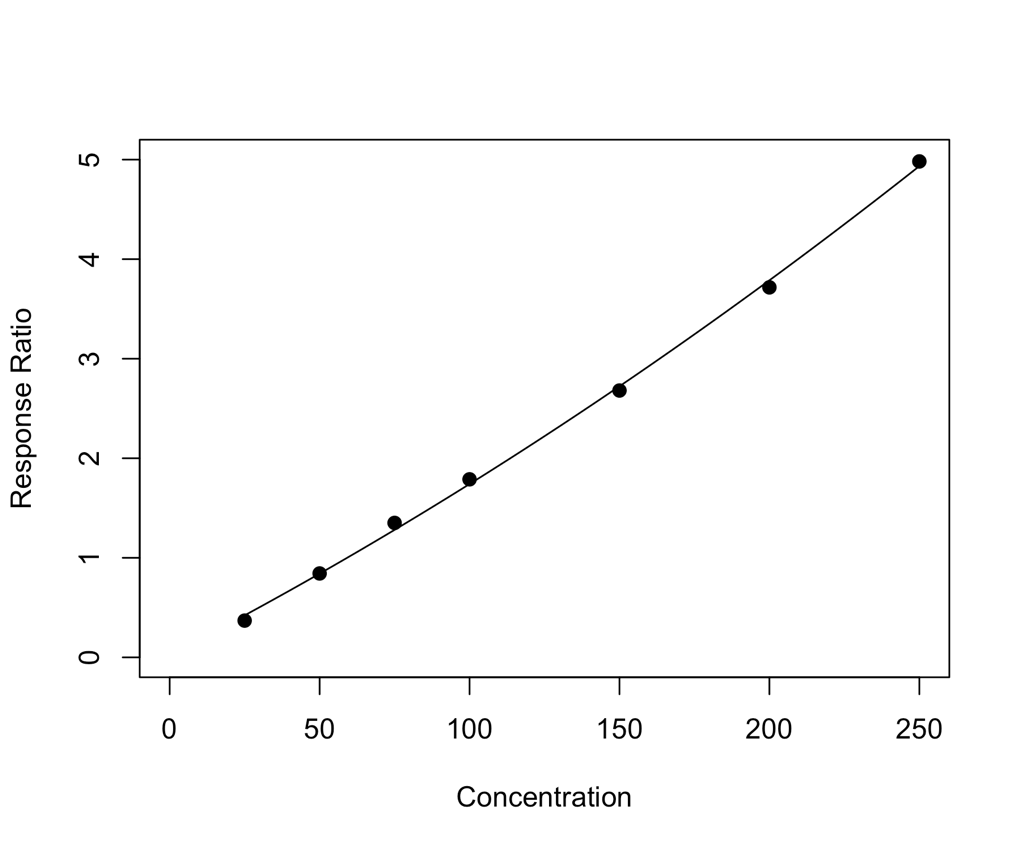


y = (1.7×10^-5^)x^2^ + 0.016x + 0.02

R^2^ = 0.999

Figure S7.1 Oxandrolone calibration curve from 10/11/2023

Table S7.2 Oxandrolone Quality Assurance Data for 10/11/2023

| **Check Standard Concentration (µg/mL)** | **Oxandrolone Peak Area** | **Internal Standard Peak Area** | **Peak Area Ratio** | **Calculated Concentration (µg/mL)** | **Bias**  **(%)** |
| --- | --- | --- | --- | --- | --- |
| 40 | 2080313 | 3204699 | 0.65 | 38.70 | 3.24 |
| 40 | 2055033 | 3149671 | 0.65 | 38.90 | 2.75 |
| 125 | 7850378 | 3396034 | 2.31 | 129.55 | 3.64 |
| 125 | 7635191 | 3175868 | 2.40 | 134.21 | 7.37 |

**7.2 Limit of Detection (LOD)**

To determine the limit of detection, oxandrolone solutions were prepared at 5, 10, 15 and 20 μg/mL. Each solution was analyzed in replicate five times and the signal to noise ratio calculated. For each replicate, the oxandrolone peak was compared to the SWGDRUG mass spectral library (version 3.11, released 1 June 2022), and the (reverse) match factor was required to be greater than 850/1,000 for positive identification.

Table S4.3: Full analytical data for calculation of limit of detection

| **Concentration (µg/mL)** | **Injection** | **Max Peak Height** | **Max Noise Height** | **R Match** | **Signal to Noise Ratio** |
| --- | --- | --- | --- | --- | --- |
| 5 | 1 | 205680 | 89782 | 899 | 2.29 |
|  | 2 | 196437 | 91475 | 873 | 2.15 |
|  | 3 | 187691 | 92286 | 890 | 2.03 |
|  | 4 | 176327 | 90352 | 876 | 1.95 |
|  | 5 | 171233 | 89867 | 882 | 1.91 |
| 10 | 1 | 306667 | 89551 | 925 | 3.42 |
|  | 2 | 313677 | 89570 | 919 | 3.50 |
|  | 3 | 301590 | 89868 | 925 | 3.36 |
|  | 4 | 305573 | 89078 | 929 | 3.43 |
|  | 5 | 317947 | 89573 | 892 | 3.55 |
| 15 | 1 | 408855 | 89583 | 933 | 4.56 |
|  | 2 | 425986 | 92690 | 937 | 4.60 |
|  | 3 | 427180 | 89167 | 935 | 4.79 |
|  | 4 | 420635 | 90225 | 933 | 4.66 |
|  | 5 | 412643 | 87208 | 938 | 4.73 |
| 20 | 1 | 566881 | 89578 | 943 | 6.33 |
|  | 2 | 586902 | 89629 | 943 | 6.55 |
|  | 3 | 572869 | 89021 | 948 | 6.44 |
|  | 4 | 560308 | 88315 | 944 | 6.34 |
|  | 5 | 565270 | 89789 | 947 | 6.30 |

Evaluation of the results demonstrated an acceptable signal to noise ratio above 10 μg/mL (>3.3) and these replicates met the requirements specified for positive identification of oxandrolone. Therefore, the LOD for this method was determined to be 10 μg/mL.

**7.3 Lower Limit of Quantitation (LLOQ)**

LLOQ was defined using the lowest non-zero calibrator. The 25 μg/mL calibration standard was analyzed across 5 separate runs and the bias was evaluated. As defined in Standard 036 (25), to be accepted, the bias of these replicates was required to fall below 20%.

Table S7.4 Analytical data for calculation of lower limit of quantitation.

| **Nominal Concentration (µg/mL)** | **Calculated Concentration (µg/mL)** |
| --- | --- |
| 25 | 25.07 |
| 25 | 22.90 |
| 25 | 22.13 |
| 25 | 24.77 |
| 25 | 21.75 |
|  |  |
| **Grand Mean** | 23.33 |
| **Bias** | 6.70% |

**Equation 4.1: Calculation of Bias (%)**

$$Bias \left( \% \right)=\left\{ \frac{Grand Mean of Calculated Concentration-Nominal Concentration}{Nominal Concentration} \right\} \times100$$

The LLOQ was defined as the lowest non-zero calibrator at 25 μg/mL with bias within 6.70% of the nominal concentration.

| **7.4 Calculation of Bias** |  |  |
| --- | --- | --- |

To evaluate bias, low, medium and high check standards for oxandrolone were analyzed over five separate runs. Each run was carried out on a different day, with a new calibration curve. Each check standard was analyzed in triplicate within each run, giving a total of 15 replicates for each concentration. The maximum acceptable bias for each concentration was defined as ±20%. This method was determined to be accurate with bias within ­­±13% across all check standards.

Table 7.5 Oxandrolone check standard data for bias calculations.

|  | **Low Check Standard (40 µg/mL)** | | **Medium Check Standard (125 µg/mL)** | | **High Check Standard (225 µg/mL)** | |
| --- | --- | --- | --- | --- | --- | --- |
|  | **Calculated Conc.** | **Bias** | **Calculated Conc.** | **Bias** | **Calculated Conc.** | **Bias** |
| **Run 1** | 40.70 | 1.76 | 133.95 | 7.16 | 240.54 | 6.91 |
|  | 39.91 | 0.22 | 136.70 | 9.36 | 238.11 | 5.82 |
|  | 38.60 | 3.50 | 132.07 | 5.66 | 229.95 | 2.20 |
| **Run 2** | 39.50 | 1.26 | 131.71 | 5.37 | 235.47 | 4.66 |
|  | 37.70 | 5.75 | 131.14 | 4.91 | 230.99 | 2.66 |
|  | 38.19 | 4.53 | 130.14 | 4.11 | 224.74 | 0.11 |
| **Run 3** | 39.17 | 2.08 | 139.53 | 11.62 | 247.72 | 10.10 |
|  | 36.11 | 9.72 | 138.00 | 10.40 | 240.53 | 6.90 |
|  | 36.91 | 7.72 | 133.81 | 7.05 | 239.89 | 6.62 |
| **Run 4** | 41.01 | 2.54 | 137.86 | 10.29 | 250.36 | 11.27 |
|  | 41.36 | 3.41 | 139.85 | 11.88 | 242.67 | 7.85 |
|  | 41.37 | 3.44 | 132.14 | 5.71 | 232.20 | 3.20 |
| **Run 5** | 38.70 | 3.24 | 129.55 | 3.64 | 238.12 | 5.83 |
|  | 38.90 | 2.75 | 134.21 | 7.37 | 231.12 | 2.72 |
|  | 37.40 | 6.50 | 128.92 | 3.13 | 223.50 | 0.67 |
|  |  |  |  |  |  |  |
| **Grand Mean** | 39.04 | 3.89 | 133.97 | 7.18 | 236.39 | 5.17 |

**7.5 Calculation of Precision**

To evaluate precision, low, medium and high check standards were analyzed over five separate runs. Each run was carried out on a different day, with a new calibration curve. Each check standard was analyzed in triplicate within each run, giving a total of 15 replicates for each concentration. Within-run and between-run precision was calculated as CV% using a single factor ANOVA. The maximum acceptable CV% for each concentration was defined as 20%. This method was determined reproducible with precision determined as being within 3% for within-run and within 14% for between-run results.

Within-run precision was calculated for each concentration using Equation 7.2, where MS_wg_ is the mean square within groups obtained from the ANOVA table.

**Equation 7.2: Calculation of Within-Group Precision.**

$$Within-Run CV \left( \% \right)= \left[ \frac{\sqrt{{MS}_{wg}}}{Grand Mean for each concentration} \right]\times100$$

Between-run precision was calculated for each concentration using Equation 7.3, where MS_bg_ is the mean square between groups obtained from the ANOVA table and *n* is the number of observations in each group.

**Equation 7.3: Calculation of Between-Group Precision.**

$$Between-Run CV \left( \% \right)= \left[ \frac{\frac{\sqrt{{MS}_{bg}}+\left( n-1 \right) \times{MS}_{wg}}{n}}{Grand Mean for each concentration} \right]\times100$$

Table 7.6 Low check standard data for precision calculations (40 µg/mL)

| Run 1  (µg/mL) | Run 2  (µg/mL) | Run 3  (µg/mL) | Run 4  (µg/mL) | Run 5  (µg/mL) |
| --- | --- | --- | --- | --- |
| 40.70 | 39.50 | 39.17 | 41.01 | 38.70 |
| 39.91 | 37.70 | 36.11 | 41.36 | 38.90 |
| 38.60 | 38.19 | 36.91 | 41.37 | 37.40 |

Anova: Single Factor

| Groups | Count | Sum | Average | Variance |
| --- | --- | --- | --- | --- |
| Run 1 | 3.00 | 119.21 | 39.74 | 1.13 |
| Run 2 | 3.00 | 115.39 | 38.46 | 0.87 |
| Run 3 | 3.00 | 112.19 | 37.40 | 2.52 |
| Run 4 | 3.00 | 123.74 | 41.25 | 0.04 |
| Run 5 | 3.00 | 115.00 | 38.33 | 0.66 |

| **Grand Mean (µg/mL)** | 39.04 |
| --- | --- |
| **Within Run Precision** | 2.62% |
| **Between Run Precision** | 3.99% |

| Source of Variation | SS | df | MS | F | P-value | F crit |
| --- | --- | --- | --- | --- | --- | --- |
| Between Groups | 26.66 | 4.00 | 6.67 | 6.39 | 0.01 | 3.48 |
| Within Groups | 10.43 | 10.00 | 1.04 |  |  |  |
| Total | 37.09 | 14.00 |  |  |  |  |

Table 7.7 Medium check standard data for precision calculations (125 µg/mL)

| Run 1  (µg/mL) | Run 2  (µg/mL) | Run 3  (µg/mL) | Run 4  (µg/mL) | Run 5  (µg/mL) |
| --- | --- | --- | --- | --- |
| 133.95 | 131.71 | 139.53 | 137.86 | 129.55 |
| 136.70 | 131.14 | 138.00 | 139.85 | 134.21 |
| 132.07 | 130.14 | 133.81 | 132.14 | 128.92 |

Anova: Single Factor

| Groups | Count | Sum | Average | Variance |
| --- | --- | --- | --- | --- |
| Run 1 | 3.00 | 402.72 | 134.24 | 5.42 |
| Run 2 | 3.00 | 392.99 | 131.00 | 0.63 |
| Run 3 | 3.00 | 411.34 | 137.11 | 8.77 |
| Run 4 | 3.00 | 409.85 | 136.62 | 16.02 |
| Run 5 | 3.00 | 392.68 | 130.89 | 8.35 |

| **Grand Mean (µg/mL)** | 133.97 |
| --- | --- |
| **Within Run Precision** | 2.09% |
| **Between Run Precision** | 5.18% |

| Source of Variation | SS | df | MS | F | P-value | F crit |
| --- | --- | --- | --- | --- | --- | --- |
| Between Groups | 105.79 | 4.00 | 26.45 | 3.37 | 0.05 | 3.48 |
| Within Groups | 78.39 | 10.00 | 7.84 |  |  |  |
| Total | 184.18 | 14.00 |  |  |  |  |

Table 7.8 High check standard data for precision calculations (225 µg/mL)

| Run 1  (µg/mL) | Run 2  (µg/mL) | Run 3  (µg/mL) | Run 4  (µg/mL) | Run 5  (µg/mL) |
| --- | --- | --- | --- | --- |
| 240.54 | 235.47 | 247.72 | 250.36 | 238.12 |
| 238.11 | 230.99 | 240.53 | 242.67 | 231.12 |
| 229.95 | 224.74 | 239.89 | 232.20 | 223.50 |

Anova: Single Factor

| Groups | Count | Sum | Average | Variance |
| --- | --- | --- | --- | --- |
| Run 1 | 3.00 | 708.60 | 236.20 | 30.77 |
| Run 2 | 3.00 | 691.20 | 230.40 | 29.04 |
| Run 3 | 3.00 | 728.14 | 242.71 | 18.90 |
| Run 4 | 3.00 | 725.23 | 241.74 | 83.09 |
| Run 5 | 3.00 | 692.74 | 230.91 | 53.47 |

| **Grand Mean (µg/mL)** | 236.39 |
| --- | --- |
| **Within Run Precision** | 2.78% |
| **Between Run Precision** | 13.56% |

| Source of Variation | SS | df | MS | F | P-value | F crit |
| --- | --- | --- | --- | --- | --- | --- |
| Between Groups | 403.66 | 4.00 | 100.91 | 2.34 | 0.13 | 3.48 |
| Within Groups | 430.56 | 10.00 | 43.06 |  |  |  |
| Total | 834.21 | 14.00 |  |  |  |  |

**7.6 Carryover**

To evaluate carryover, the highest oxandrolone calibration standard (250 µg/mL) and subsequent methanol blank were analyzed over three separate runs. Each run was carried out on a different day, with a new calibration curve. This method was determined to produce no carryover.

A


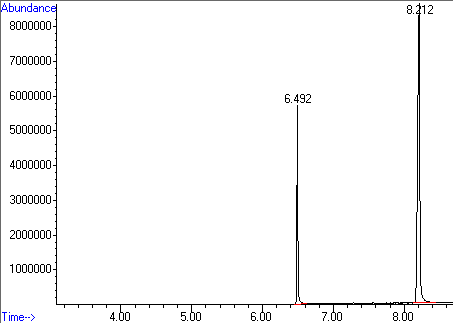

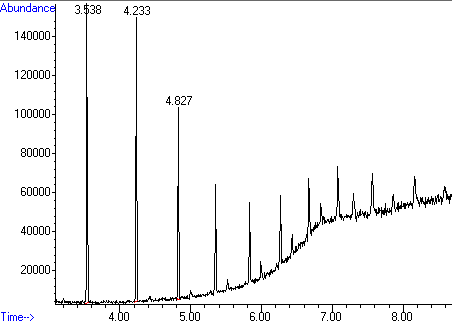


B


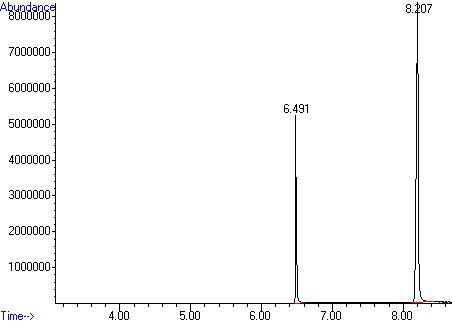

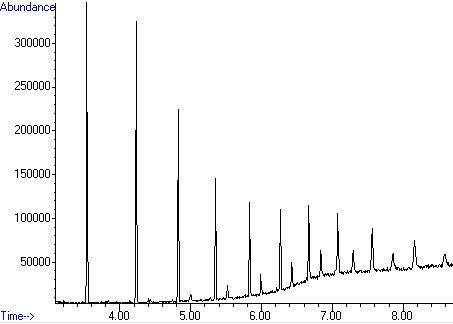


C


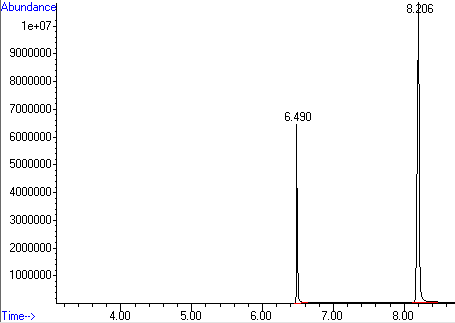

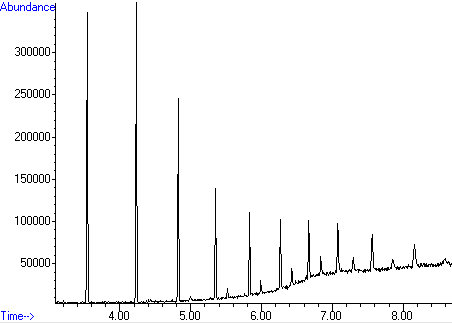


Figure S7.2 Oxandrolone 250 µg/mL calibration standard (left) and subsequent methanol blank (right) run on three different days: (A) on 19-10-2023, (B) on 23-10-2023, and (C) on 25-10-2023.

**Section 8: Full qualitative data for samples found to contain AASs seized in Scottish prisons.**

**Table 8.1.** Full AAS qualitative data for seized samples from GC-MS analysis, including retention time (RT), RT of reference (ref) standard, MS library R-Match, and mass-to-charge ratio (m/z). Due to security concerns, the prison establishment is provided as a number.

| **Sample ID** | **Prison ID** | **Date** | **Initial visual description** | **Compound Identified** | **RT (mins)** | **Ref Standard**  **RT (mins)** | **R-Match (x/1000)** | **m/z (a.m.u)** |
| --- | --- | --- | --- | --- | --- | --- | --- | --- |
|  |  |  |  |  |  |  |  |  |
| FL22/0706-1 | 3 | 15/07/2022 | 17x Orange tablets | Stanozolol | 10.144 | 10.165 | 912 | 328 |
| FL22/0706-2 |  |  | 1x Pink tablet | Methyltestosterone | 8.138 | 7.812 | 946 | 302 |
| FL22/0741 | 1 | 22/08/2022 | 1x White tablet fragment | Methyltestosterone | 7.810 | 7.812 | 960 | 302 |
|  |  |  |  | Metandienone | 7.950 | 7.928 | 986 | 300 |
| FL22/0773 | 1 | 18/07/2022 | 1x Round white tablet | Methyltestosterone | 7.809 | 7.812 | 947 | 302 |
|  |  |  |  | Metandienone | 7.947 | 7.928 | 970 | 300 |
| FL22/0815 | 3 | 25/09/2022 | 38x White round tablets | Oxandrolone | 8.125 | 8.105 | 965 | 306 |
|  |  |  |  | Methyltestosterone | 7.809 | 7.812 | 950 | 302 |
|  |  |  |  | Metandienone | 7.967 | 7.928 | 927 | 300 |
| FL22/0957 | 1 | 10/10/2022 | 1x Sample tube containing white powder | Oxandrolone | 8.020 | NA | 930 | 306 |
|  |  |  |  | Etizolam | 9.887 | 9.955 | 923 | 342 |
| FL22/0958 | 1 | 10/10/2022 | 1x Sample tube containing yellow powder | Oxandrolone | 8.101 | 8.105 | 965 | 306 |
|  |  |  |  | Stanozolol | 10.142 | 10.165 | 942 | 328 |
| FL22/0979 | 1 | 27/10/2022 | 1x Sample tube containing white powder | Oxandrolone | 8.077 | 8.105 | 967 | 306 |
| FL22/0982 | 1 | 23/11/2022 | 1x Round white tablet | Metandienone | 7.946 | 7.928 | 968 | 300 |
|  |  |  |  | Methyltestosterone | 7.812 | 7.812 | 949 | 302 |
| FL22/0993-1 | 11 | 04/06/2022 | 1x Round white tablet | Methyltestosterone | 7.815 | 7.812 | 973 | 302 |
|  |  |  |  | Metandienone | 7.948 | 7.928 | 965 | 300 |
| FL22/0993-2 |  |  | 1x Round light blue tablet | Oxandrolone | 8.101 | 8.105 | 969 | 306 |
|  |  |  |  | Methyltestosterone | 7.809 | 7.812 | 897 | 302 |
|  |  |  |  | Metandienone | 7.955 | 7.928 | 930 | 300 |
| FL22/0993-3 |  |  | 1x Round pink tablet | Oxandrolone | 8.099 | 8.105 | 966 | 306 |
| FL22/0993-4 |  |  | 3x White tablets | Oxymetholone | 8.254 | 8.251 | 958 | 332 |
| FL23/0055 | 5 | 10/10/2022 | 2x Fragments of green tablet | Methyltestosterone | 7.810 | 7.812 | 967 | 302 |
|  |  |  |  | Oxymetholone | 8.343 | 8.339 | 937 | 332 |
|  |  |  |  | Androstenedione | 7.829 | NA | 935 | 286 |
| FL23/0081 | 9 | 29/08/2022 | 49x White round tablets | Mestanolone | 7.510 | 7.517 | 963 | 304 |
|  |  |  |  | Oxymetholone | 8.253 | 8.251 | 957 | 332 |
| FL23/0095 | 4 | 09/02/2023 | 5x Round yellow tablets | Oxandrolone | 8.117 | 8.105 | 969 | 306 |
| FL23/0097-1 | 4 | 14/03/2023 | 57x Pink tablets | Oxymetholone | 8.272 | 8.251 | 960 | 332 |
| FL23/0097-2 |  |  | 1.5x white tablets | Oxymetholone | 8.215 | 8.251 | 949 | 332 |
|  |  |  |  | Zopiclone | 10.475 | NA | 963 | 388 |
| FL23/0099 | 4 | 06/03/2023 | 3x Round yellow tablets | Oxandrolone | 8.123 | 8.105 | 969 | 306 |
| FL23/0102 | 1 | 11/04/2023 | 1x Round white tablet | Metandienone | 7.947 | 7.928 | 964 | 300 |
|  |  |  |  | Methyltestosterone | 7.807 | 7.812 | 942 | 302 |
| FL23/0124 | 11 | 08/02/2023 | Loose blue powder in bag | Oxandrolone | 8.119 | 8.105 | 957 | 306 |
| FL23/0133 | 11 | 03/04/2023 | 1x Clear wrap blue powder | Oxandrolone | 8.112 | 8.105 | 953 | 306 |
| FL23/0180-2 | 3 | 03/01/2023 | 50x White round tablets | Stanozolol | 10.141 | 10.165 | 926 | 328 |
|  |  |  |  | Oxymetholone | 8.346 | 8.339 | 956 | 332 |
|  |  |  |  | Metandienone | 7.956 | 7.928 | 971 | 300 |
|  |  |  |  | Methyltestosterone | 7.822 | 7.812 | 935 | 302 |
| FL23/0189 | 1 | 15/05/2023 | 1x Round green tablet | Oxymetholone | 8.345 | 8.339 | 944 | 332 |
| FL23/0203 | 3 | 24/04/2023 | 2x Whole yellow tablets | Oxandrolone | 8.097 | 8.105 | 966 | 306 |
| FL23/0205-1 | 6 | 14/03/2023 | 1x White tablet fragment | Metandienone | 7.958 | 7.928 | 968 | 300 |
|  |  |  |  | Methyltestosterone | 7.808 | 7.812 | 919 | 302 |
| FL23/0205-2 |  |  | 12x Round white tablets | Metandienone | 7.958 | 7.928 | 968 | 300 |
|  |  |  |  | Methyltestosterone | 7.809 | 7.812 | 924 | 302 |
| FL23/0205-3 |  |  | Clingfilm wrap with trace amount of pink powder | Metandienone | 7.937 | 7.928 | 970 | 300 |
|  |  |  |  | Methyltestosterone | 7.955 | NA | 913 | 302 |
| FL23/0210 | 6 | 24/03/2023 | 20x Red tablets | Metandienone | 7.937 | 7.928 | 966 | 300 |
|  |  |  |  | Methyltestosterone | 7.807 | 7.812 | 927 | 302 |
| FL23/0227 | 3 | 20/03/2023 | Red tablets | Metandienone | 7.944 | 7.928 | 964 | 300 |
|  |  |  |  | Methyltestosterone | 7.804 | 7.812 | 925 | 302 |
| FL23/0251 | 3 | 03/06/2023 | 1x paper wrap with green powder, crushed tablets, and whole tablet with score | Oxymetholone | 8.256 | 8.251 | 955 | 332 |
| FL23/0269 | 4 | 27/03/2023 | 20x Broken yellow tablets | Oxandrolone | 8.131 | 8.105 | 966 | 306 |
| FL23/0270-1 | 4 | 03/03/2023 | 2x Round light pink tablets | Oxymetholone | 8.333 | 8.345 | 948 | 332 |
| FL23/0270-2 |  |  | 3x Round reddish pink tablets | Oxymetholone | 8.337 | 8.345 | 957 | 332 |
| FL23/0271 | 4 | 13/05/2023 | Approx. 20x round white tablets | Oxymetholone | 8.346 | 8.345 | 949 | 332 |
| FL23/0272 | 4 | 13/05/2023 | 1x Blue oblong shaped tablet | Oxymetholone | 8.346 | 8.345 | 951 | 332 |
| FL23/0274 | 4 | 13/05/2023 | 1x Round white tablet | Oxymetholone | 8.256 | 8.251 | 892 | 332 |
|  |  |  |  | Zopiclone | 10.991 | NA | 925 | 388 |
|  |  |  |  | Sildenafil | 9.114 | NA | 855 | 474 |
| FL23/0275-1 | 4 | 20/04/2023 | 1x Round yellow tablet | Oxandrolone | 8.127 | 8.105 | 959 | 306 |
| FL23/0276-1 | 4 | 17/03/2023 | 7x Round pink tablets | Oxymetholone | 8.343 | 8.345 | 957 | 332 |
| FL23/0277 | 4 | 07/04/2023 | 6x Whole & 1 half tablet | Oxymetholone | 8.318 | 8.345 | 943 | 332 |
|  |  |  |  | Mirtazapine | 6.501 | NA | 971 | 265 |
| FL23/0278 | 4 | 28/04/2023 | 1x White hexagonal shaped tablet | Oxymetholone | 8.334 | 8.345 | 951 | 332 |
| FL23/0280 | 4 | 13/05/2023 | Large amount of round white tablets | Oxymetholone | 8.255 | 8.251 | 955 | 332 |
| FL23/0292 | 3 | 23/05/2023 | 2x Round white tablet | Metandienone | 7.935 | 7.928 | 964 | 300 |
|  |  |  |  | Oxymetholone | 8.248 | 8.251 | 945 | 332 |
|  |  |  |  | Stanozolol | 10.134 | 10.165 | 888 | 328 |
| FL23/0308 | 12 | 15/11/2022 | 1x white tablet | Oxymetholone | 8.385 | 8.345 | 889 | 332 |
|  |  |  |  | Sertraline | 6.792 | NA | 970 | 305 |
| FL23/0316 | 5 | 27/02/2023 | 50x round white tablets | MDMB-INACA | 7.004 | 7.019 | 885 | 289 |
|  |  |  |  | Oxymetholone | 8.374 | 8.389 | 927 | 332 |
| FL23/0317 | 5 | Jul-23 | Large amount of green tablet fragments | Methyltestosterone | 7.857 | 7.811 | 964 | 302 |
|  |  |  |  | Mestanolone | 7.500 | 7.509 | 947 | 304 |
| FL23/0320 | 5 | 11/03/2023 | 4x whole purple tablets | Oxymetholone | 8.257 | 8.251 | 949 | 332 |
|  |  |  |  | Metandienone | 7.933 | 7.928 | 966 | 300 |
| FL23/0323 | 5 | 11/03/2023 | Loose herbal material | Metandienone | 9.245 | NA | 928 | 300 |
|  |  |  |  | Δ^9^-THC | 8.640 | NA | 970 | 314 |
| FL23/0328-1 | 6 | 14/07/2023 | 1x Round blue tablet | Oxymetholone | 8.351 | 8.345 | 930 | 332 |
|  |  |  |  | Amitriptyline | 6.267 | NA | 960 | 277 |
| FL23/0341 | 7 | 25/07/2023 | 1x Round white tablet | Oxymetholone | 8.370 | 8.389 | 931 | 332 |
| FL23/0344 | 7 | 08/07/2023 | Small amount of orange substance | Etizolam | 10.468 | 10.267 | 945 | 342 |
|  |  |  |  | Oxymetholone | 8.372 | 8.389 | 950 | 332 |
|  |  |  |  | Mestanolone | 7.500 | 7.518 | 950 | 304 |
| FL23/0346 | 7 | 22/06/2023 | 23x round yellow tablets | Oxymetholone | 8.374 | 8.389 | 946 | 332 |
| FL23/0352 | 10 | 15/02/2023 | 1x bag crushed soap-like material | Mestanolone | 7.500 | 7.518 | 948 | 304 |
| FL23/0358 | 10 | 15/03/2023 | Approx. 20x round pink tablets | Oxymetholone | 8.271 | 8.251 | 944 | 332 |
|  |  |  |  | Metandienone | 7.945 | 7.928 | 886 | 304 |
| FL23/0359 | 10 | 05/04/2023 | 4x Round green tablets + 2x fragments | Oxymetholone | 8.347 | 8.345 | 947 | 332 |
| FL23/0360 | 10 | 02/05/2023 | 10x Round blue tablets | Oxymetholone | 8.351 | 8.345 | 905 | 332 |
|  |  |  |  | Amitriptyline | 6.264 | NA | 967 | 277 |

**Section 9: Example photographs of other samples types seized from the Scottish prisons found positive for AASs.**


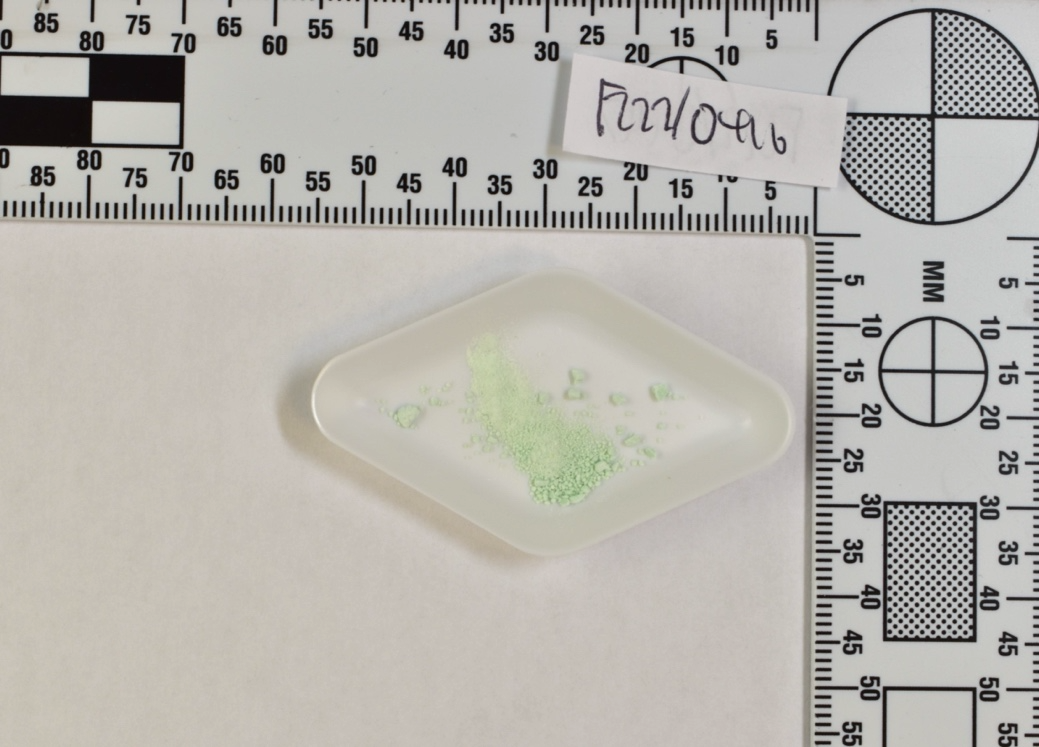

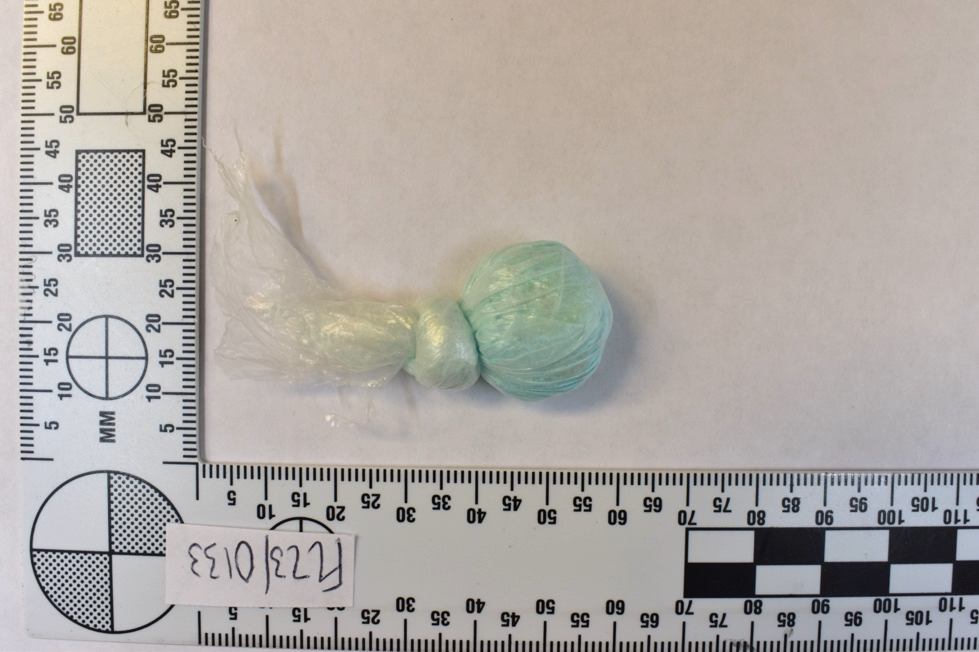


(b)

(a)

(c)

(d)


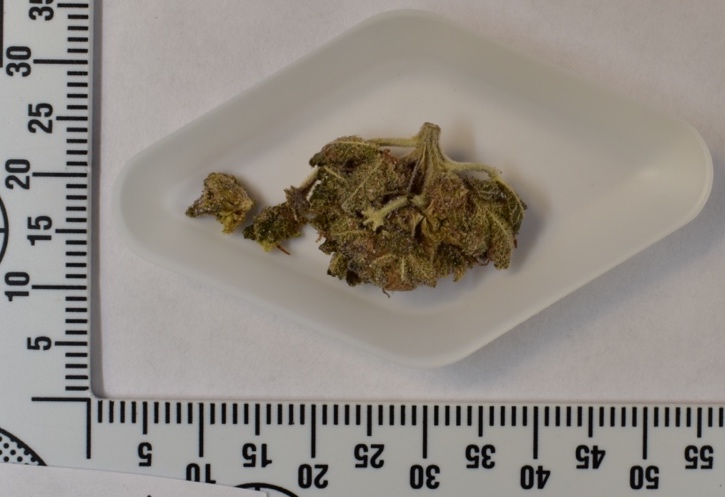

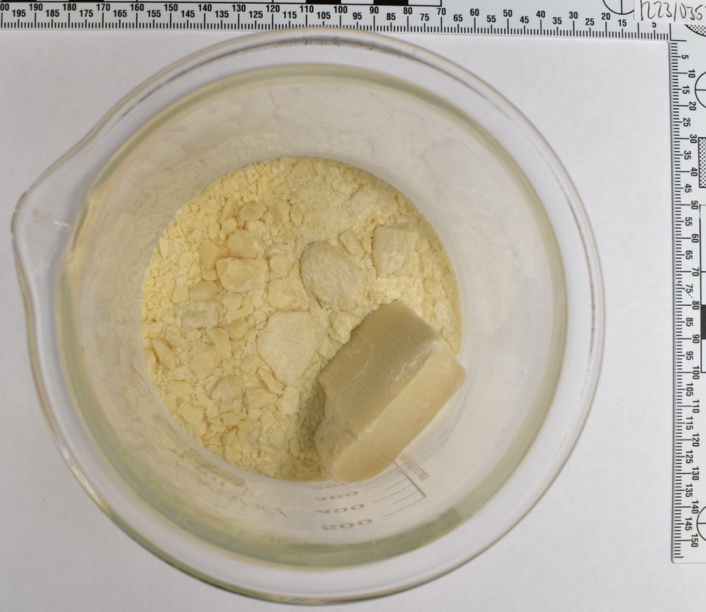


**Figure 9.1.** Examples of other sample types seized from the Scottish prisons found positive for AASs: (a) green powder seized 7^th^ February 2022 found positive for oxymetholone and mestanolone; (b) blue powder seized 3^rd^ April 2023 found positive for oxandrolone; (c) herbal material seized 11^th^ March 2023 found positive for metandienone and Δ^9^-THC; and (d) pieces of crushed soap bar-like material seized 15^th^ February 2023 found positive for mestanolone.

**Section 10: Full tablet markings data for samples of tablets found to contain AASs seized in Scottish prisons.**

**Table 10.1.** Full tablet markings data for samples of tablets found to contain AASs seized in Scottish prisons. For samples that only contained fragments, so the shape and markings of the tablets could not be determined, “NA” is written in the table. “opp” indicates marking on opposite side of the tablet.

| **Sample ID** | **Prison ID** | **Date** | **Steroid Identified** | **Colour** | **Shape** | **Markings** |
| --- | --- | --- | --- | --- | --- | --- |
| FL22/0706-1 | 3 | 15/07/2022 | Stanozolol | Peach | Round | Score |
| FL22/0706-2 |  |  | Methyltestosterone | Pink | Round | Unknown |
| FL22/0741 | 1 | 22/08/2022 | Methyltestosterone | White | Round | Score |
|  |  |  | Metandienone |  |  |  |
| FL22/0773 | 1 | 18/07/2022 | Methyltestosterone | White | Round | Score |
|  |  |  | Metandienone |  |  |  |
| FL22/0815 | 3 | 25/09/2022 | Oxandrolone | White | Round | Score |
|  |  |  | Methyltestosterone |  |  |  |
|  |  |  | Metandienone |  |  |  |
| FL22/0982 | 1 | 23/11/2022 | Metandienone | White | Round | Score |
|  |  |  | Methyltestosterone |  |  |  |
| FL22/0993-1 | 11 | 04/06/2022 | Methyltestosterone | White | Round | Score |
|  |  |  | Metandienone |  |  |  |
| FL22/0993-2 |  |  | Oxandrolone | Light blue | Round | Score |
|  |  |  | Methyltestosterone |  |  |  |
|  |  |  | Metandienone |  |  |  |
| FL22/0993-3 |  |  | Oxandrolone | Light pink | Round | Score |
| FL22/0993-4 |  |  | Oxymetholone | White | Round | Score |
| FL23/0055 | 5 | 10/10/2022 | Methyltestosterone | Green | NA | NA |
|  |  |  | Oxymetholone |  |  |  |
|  |  |  | Androstenedione |  |  |  |
| FL23/0081 | 9 | 29/08/2022 | Mestanolone | White | Round | Score |
|  |  |  | Oxymetholone |  |  |  |
| FL23/0095 | 4 | 09/02/2023 | Oxandrolone | Yellow | Round | Score |
| FL23/0097-1 | 4 | 14/03/2023 | Oxymetholone | Pink | Round | Score |
| FL23/0097-2 |  |  | Oxymetholone | White | Oval | E/L |
|  |  |  | Zopiclone |  |  |  |
| FL23/0099 | 4 | 06/03/2023 | Oxandrolone | Yellow | Round | Score |
| FL23/0102 | 1 | 11/04/2023 | Metandienone | White | Round | Score |
|  |  |  | Methyltestosterone |  |  |  |
| FL23/0180-2 | 3 | 03/01/2023 | Stanozolol | White | Round | Score |
|  |  |  | Oxymetholone |  |  |  |
|  |  |  | Metandienone |  |  |  |
|  |  |  | Methyltestosterone |  |  |  |
| FL23/0189 | 1 | 15/05/2023 | Oxymetholone | Green | Round | SHREE opp Score |
| FL23/0203 | 3 | 24/04/2023 | Oxandrolone | Yellow | Round | C/R |
| FL23/0205-1 | 6 | 14/03/2023 | Metandienone | White | NA | NA |
|  |  |  | Methyltestosterone |  |  |  |
| FL23/0205-2 |  |  | Metandienone | White | Round | None |
|  |  |  | Methyltestosterone |  |  |  |
| FL23/0210 | 6 | 24/03/2023 | Metandienone | Red | Round | Score |
|  |  |  | Methyltestosterone |  |  |  |
| FL23/0227 | 3 | 20/03/2023 | Metandienone | Red | Round | Score |
|  |  |  | Methyltestosterone |  |  |  |
| FL23/0251 | 3 | 03/06/2023 | Oxymetholone | Green | Round | Score |
| FL23/0269 | 4 | 27/03/2023 | Oxandrolone | Yellow | Round | Score |
| FL23/0270-1 | 4 | 03/03/2023 | Oxymetholone | Light pink | Round | None |
| FL23/0270-2 |  |  | Oxymetholone | Red | Round | Score |
| FL23/0271 | 4 | 13/05/2023 | Oxymetholone | White | Round | Score |
|  |  |  | Metandienone |  |  |  |
| FL23/0272 | 4 | 13/05/2023 | Oxymetholone | Blue coated | Egg | 100 |
| FL23/0274 | 4 | 13/05/2023 | Oxymetholone | White | Round | Score |
|  |  |  | Zopiclone |  |  |  |
|  |  |  | Sildenafil |  |  |  |
| FL23/0275-1 | 4 | 20/04/2023 | Oxandrolone | Yellow | Round | Score |
| FL23/0276-1 | 4 | 17/03/2023 | Oxymetholone | Red | Round | Score |
| FL23/0277 | 4 | 07/04/2023 | Oxymetholone | Orange-red coated | Oval | I/ opp score |
|  |  |  | Mirtazapine |  |  |  |
| FL23/0278 | 4 | 28/04/2023 | Oxymetholone | White | Hexagonal | None |
| FL23/0280 | 4 | 13/05/2023 | Oxymetholone | White | Round | None |
| FL23/0292 | 3 | 23/05/2023 | Metandienone | White | Round | Score |
|  |  |  | Oxymetholone |  |  |  |
|  |  |  | Stanozolol |  |  |  |
| FL23/0308 | 12 | 15/11/2022 | Oxymetholone | White | Oblong | 100 opp score |
|  |  |  | Sertraline |  |  |  |
| FL23/0316 | 5 | 27/02/2023 | MDMB-INACA | White | Round | T 50 opp score |
|  |  |  | Oxymetholone |  |  |  |
| FL23/0317 | 5 | Jul-23 | Methyltestosterone | Green | NA | NA |
| FL23/0320 | 5 | 11/03/2023 | Oxymetholone | Purple | Round | Score |
|  |  |  | Metandienone |  |  |  |
| FL23/0328-1 | 6 | 14/07/2023 | Oxymetholone | Blue | Round | C opp AA |
|  |  |  | Amitriptyline |  |  |  |
| FL23/0341 | 7 | 25/07/2023 | Oxymetholone | White | Round | None |
| FL23/0346 | 7 | 22/06/2023 | Oxymetholone | Yellow | Round | None |
| FL23/0358 | 10 | 15/03/2023 | Oxymetholone | Pink | Round | Score |
|  |  |  | Metandienone |  |  |  |
| FL23/0359 | 10 | 05/04/2023 | Oxymetholone | Green | Round | SHREE opp Score |
| FL23/0360 | 10 | 02/05/2023 | Oxymetholone | Blue | Round | D |
|  |  |  | Amitriptyline |  |  |  |

**Section 11: Full quantitative data for samples found to contain AASs seized in Scottish Prisons.**

**Table 11.1.** Full AAS quantitation data for seized samples.

| **Sample ID** | **Compound Identified** | **Sample Format** | **Mass of single tablet or powder (mg)** | **Response** | **IS Response** | **Response Ratio** | **Conc. (ug/mL)** | **Mass of powder sampled (mg)** | **Dilution** | **Conc. (ug/mg)** | **% AAS in sample** | **AAS per tablet (mg)** |
| --- | --- | --- | --- | --- | --- | --- | --- | --- | --- | --- | --- | --- |
| FL22/0706-1 | Stanozolol | Tablet | 369.6 | 3727367 | 29657727 | 0.13 | 80.36 | 10.0 | 10 | 40.18 | 4.02% | 14.85 |
| FL22/0706-2 | Methyltestosterone | Tablet | 218.3 | <LOQ | | | | | | | | |
| FL22/0741 | Methyltestosterone | Tablet | 174.8 | <LOQ | | | | | | | | |
|  | Metandienone |  |  | 136097318 | 52395888 | 2.60 | 162.92 | 10.0 | 20 | 162.92 | 16.29% |  |
| FL22/0773 | Methyltestosterone | Tablet | 265.7 | <LOQ | | | | | | | | |
|  | Metandienone |  |  | 98945280 | 56034109 | 1.77 | 111.48 | 10.0 | 20 | 111.48 | 11.15% | 29.62 |
| FL22/0815 | Oxandrolone | Tablet | 155.3 | 10334498 | 2972682 | 3.48 | 173.57 | 10.0 | 20 | 173.57 | 17.36% | 26.96 |
|  | Methyltestosterone |  |  | 13826444 | 55801565 | 0.25 | 25.93 | 10.0 | 2 | 2.59 | 0.26% | 0.40 |
|  | Metandienone |  |  | <LOQ | | | | | | | | |
| FL22/0957 | Oxandrolone | Powder | 485.7 | <LOQ | | | | | | | | |
| FL22/0958 | Oxandrolone | Powder | 324.8 | 11579682 | 3227088 | 3.59 | 190.92 | 10.1 | 10 | 95.46 | 9.55% | - |
|  | Stanozolol |  |  | 8565250 | 28229300 | 0.30 | 129.44 | 10.1 | 10 | 64.08 | 6.41% | - |
| FL22/0979 | Oxandrolone | Powder | 197.9 | 7663091 | 3276234 | 2.34 | 130.93 | 10.0 | 10 | 65.47 | 6.55% | - |
| FL22/0982 | Metandienone | Tablet | 253 | 160904788 | 55998620 | 2.87 | 172.34 | 10.0 | 40 | 344.69 | 34.47% | 87.21 |
|  | Methyltestosterone |  |  | 15493329 | 60189004 | 0.26 | 26.41 | 10.0 | 2 | 2.64 | 0.26% | 0.67 |
| FL22/0993-1 | Methyltestosterone | Tablet | 106.6 | 119227308 | 43276992 | 2.75 | 160.16 | 10.0 | 10 | 80.08 | 8.01% | 8.54 |
|  | Metandienone |  |  | 126514787 | 48578489 | 2.60 | 160.35 | 10.0 | 30 | 240.53 | 24.05% | 25.64 |
| FL22/0993-2 | Oxandrolone | Tablet | 197.5 | 4878136 | 3090370 | 1.58 | 91.30 | 10.0 | 10 | 45.65 | 4.56% | 9.02 |
|  | Methyltestosterone |  |  | <LOQ | | | | | | | | |
|  | Metandienone |  |  | <LOQ | | | | | | | | |
| FL22/0993-3 | Oxandrolone | Tablet | 207.3 | 4892111 | 3135738 | 1.56 | 90.31 | 10.0 | 10 | 45.15 | 4.52% | 9.36 |
| FL22/0993-4 | Oxymetholone | Tablet | 123.3 | 4351221 | 3584098 | 1.21 | 156.20 | 10.0 | 30 | 234.31 | 23.43% | 28.89 |
| FL23/0055 | Methyltestosterone | Tablet | 147.1 | 78061549 | 44015263 | 1.77 | 103.52 | 10.0 | 10 | 51.76 | 5.18% | 7.61 |
|  | Oxymetholone |  |  | 8593726 | 50216311 | 0.17 | 57.95 | 10.0 | 2 | 5.80 | 0.58% | 0.85 |
|  | Androstenedione |  |  | NA | | | | | | | | |
| FL23/0081 | Mestanolone | Tablet | 114.7 | 36309112 | 57278663 | 0.63 | 40.86 | 10.0 | 2 | 4.09 | 0.41% | 0.47 |
|  | Oxymetholone |  |  | 1546516 | 3475962 | 0.44 | 91.03 | 10.1 | 30 | 135.19 | 13.52% | 15.51 |
| FL23/0095 | Oxandrolone | Tablet | 188.8 | 13583352 | 2979031 | 4.56 | 219.34 | 10.0 | 20 | 219.34 | 21.93% | 41.41 |
| FL23/0097-1 | Oxymetholone | Tablet | 183.4 | 6768608 | 3632270 | 1.86 | 190.63 | 10.0 | 20 | 190.63 | 19.06% | 34.96 |
| FL23/0097-2 | Oxymetholone | Tablet | 143.2 | <LOQ | | | | | | | | |
| FL23/0099 | Oxandrolone | Tablet | 185.5 | 10134169 | 2953614 | 3.43 | 171.59 | 10.0 | 20 | 171.59 | 17.16% | 31.83 |
| FL23/0102 | Metandienone | Tablet | 217.6 | 107847503 | 54931965 | 1.96 | 123.56 | 10.0 | 10 | 61.78 | 6.18% | 13.44 |
|  | Methyltestosterone |  |  | <LOQ | | | | | | | | |
| FL23/0124 | Oxandrolone | Powder | 840.7 | 13346767 | 2795196 | 4.77 | 228.14 | 10.0 | 20 | 228.14 | 22.81% | - |
| FL23/0133 | Oxandrolone | Powder | 1017.8 | 9919017 | 3303592 | 3.00 | 152.69 | 10.0 | 20 | 152.69 | 15.27% | - |
| FL23/0180-2 | Stanozolol | Tablet | 124.5 | 15185472 | 30843627 | 0.49 | 174.16 | 10.0 | 10 | 87.08 | 8.71% | 10.84 |
|  | Oxymetholone |  |  | 93246976 | 47168086 | 1.98 | 214.93 | 9.9 | 10 | 108.55 | 10.86% | 13.51 |
|  | Metandienone |  |  | 180869581 | 57022841 | 3.17 | 199.36 | 10.0 | 10 | 99.68 | 9.97% | 12.41 |
|  | Methyltestosterone |  |  | <LOQ | | | | | | | | |
| FL23/0189 | Oxymetholone | Tablet | 153.9 | 90567330 | 48005932 | 1.89 | 207.99 | 10.0 | 20 | 207.99 | 20.80% | 32.01 |
| FL23/0203 | Oxandrolone | Tablet | 196 | 3502614 | 3081443 | 1.14 | 66.94 | 10.0 | 10 | 33.47 | 3.35% | 6.56 |
| FL23/0205-1 | Metandienone | Tablet | 22.4 | 207360837 | 62816118 | 3.30 | 207.66 | 10.0 | 10 | 103.83 | 10.38% | 2.33 |
|  | Methyltestosterone |  |  | <LOQ | | | | | | | | |
| FL23/0205-2 | Metandienone | Tablet | 122 | 190777996 | 60096167 | 3.17 | 199.53 | 10.0 | 10 | 99.77 | 9.98% | 12.17 |
|  | Methyltestosterone |  |  | <LOQ | | | | | | | | |
| FL23/0205-3 | Metandienone | Powder | 2.5 | NA | | | | | | | | |
|  | Methyltestosterone |  |  |  |  |  |  |  |  |  |  |  |
| FL23/0210 | Metandienone | Tablet | 212.4 | 32318254 | 55835113 | 0.58 | 40.52 | 10.0 | 10 | 20.26 | 2.03% | 4.30 |
|  | Methyltestosterone |  |  | <LOQ | | | | | | | | |
| FL23/0227 | Metandienone | Tablet | 204.1 | 79233679 | 54103372 | 1.46 | 93.20 | 10.0 | 10 | 46.60 | 4.66% | 9.51 |
|  | Methyltestosterone |  |  | <LOQ | | | | | | | | |
| FL23/0251 | Oxymetholone | Tablet | 235.3 | 3968540 | 3367315 | 1.18 | 153.45 | 10.0 | 20 | 153.45 | 15.35% | 36.11 |
| FL23/0269 | Oxandrolone | Tablet | 195.2 | 12062589 | 2876576 | 4.19 | 204.15 | 10.0 | 20 | 204.15 | 20.41% | 39.85 |
| FL23/0270-1 | Oxymetholone | Tablet | 181.5 | 16456963 | 43808774 | 0.38 | 78.16 | 10.1 | 20 | 77.38 | 7.74% | 14.04 |
| FL23/0270-2 | Oxymetholone | Tablet | 197.3 | 19192641 | 43450426 | 0.44 | 84.51 | 10.1 | 20 | 83.68 | 8.37% | 16.51 |
| FL23/0271 | Oxymetholone | Tablet | 116.6 | 93438923 | 42106880 | 2.22 | 233.18 | 10.0 | 30 | 349.77 | 34.98% | 40.78 |
| FL23/0272 | Oxymetholone | Tablet | 611.4 | <LOQ | | | | | | | | |
| FL23/0274 | Oxymetholone | Tablet | 172.7 | <LOQ | | | | | | | | |
| FL23/0275-1 | Oxandrolone | Tablet | 185.2 | 10169314 | 2751390 | 3.70 | 183.06 | 10.0 | 20 | 183.06 | 18.31% | 33.90 |
| FL23/0276-1 | Oxymetholone | Tablet | 201.1 | 62089770 | 41709779 | 1.49 | 176.48 | 10.2 | 20 | 173.02 | 17.30% | 34.79 |
| FL23/0277 | Oxymetholone | Tablet | 302.1 | <LOQ | | | | | | | | |
| FL23/0278 | Oxymetholone | Tablet | 209.8 | 17721847 | 48859997 | 0.36 | 76.90 | 10.1 | 20 | 76.14 | 7.61% | 15.97 |
| FL23/0280 | Oxymetholone | Tablet | 307.7 | 3517858 | 3497024 | 1.01 | 139.77 | 10.0 | 20 | 139.77 | 13.98% | 43.01 |
| FL23/0292 | Metandienone | Tablet | 126.4 | 77455758 | 59932457 | 1.29 | 76.53 | 10.0 | 10 | 38.26 | 3.83% | 4.84 |
|  | Oxymetholone |  |  | 1302951 | 3420942 | 0.38 | 84.96 | 10.0 | 20 | 84.96 | 8.50% | 10.74 |
|  | Stanozolol |  |  | 7507079 | 32273422 | 0.23 | 110.90 | 10.0 | 10 | 55.45 | 5.54% | 7.01 |
| FL23/0308 | Oxymetholone | Tablet | 313.9 | 14336873 | 66008513 | 0.22 | 82.40 | 9.9 | 1 | 4.16 | 0.42% | 1.31 |
| FL23/0316 | Oxymetholone | Tablet | 205.1 | <LOQ | | | | | | | | |
| FL23/0317 | Methyltestosterone | Tablet | 94.8 | 137754398 | 56619777 | 2.43 | 140.72 | 10.0 | 5 | 35.18 | 3.52% | - |
|  | Mestanolone |  |  | 12235345 | 56619777 | 0.22 | 24.34 | 10.0 | 5 | 6.09 | 0.61% | - |
| FL23/0320 | Oxymetholone | Tablet | 183.9 | 125716 | 3513612 | 0.04 | 49.79 | 10.1 | 20 | 49.30 | 4.93% | 9.07 |
|  | Metandienone |  |  | <LOQ | | | | | | | | |
| FL23/0328-1 | Oxymetholone | Tablet | 103.7 | <LOQ | | | | | | | | |
| FL23/0341 | Oxymetholone | Tablet | 1523.5 | <LOQ | | | | | | | | |
| FL23/0344 | Oxymetholone | Powder | 10.1 | NA | | | | | | | | |
|  | Mestanolone |  |  |  |  |  |  |  |  |  |  |  |
| FL23/0346 | Oxymetholone | Tablet | 761.2 | <LOQ | | | | | | | | |
| FL23/0358 | Oxymetholone | Tablet | 185.2 | 6792607 | 3225965 | 2.11 | 204.74 | 9.9 | 20 | 206.81 | 20.68% | 38.30 |
|  | Metandienone |  |  | 184450485 | 59369682 | 3.11 | 189.18 | 10.0 | 10 | 94.59 | 9.46% | 17.52 |
| FL23/0359 | Oxymetholone | Tablet | 152.6 | 100154176 | 40648790 | 2.46 | 251.13 | 10.0 | 20 | 251.13 | 25.11% | 38.32 |
| FL23/0360 | Oxymetholone | Tablet | 128.1 | <LOQ | | | | | | | | |

**Section 12: R Script for quantitation**

#========================

# CALIBRATION CURVE

#========================

library(readxl)

library(writexl)

#Import the Responses of the Calibration Standards (With Columns of Sample, Response, ISResponse)

CalibData <- read_xlsx("AASQuant_13112023_Oxymetholone.xlsx",1) ; is.numeric(CalibData$Sample[1:11]) ; CalibData

#Calculate the Response Ratios for the three data sets

CalibData[4] <- CalibData[2] / CalibData[3] ; names(CalibData)[4] <- "ResponseRatio" ; CalibData

#Calculate a Quadratic Regression for the Calibration Curve

QuadRegression <- function(x,y){

xi4 <- 0

xi3 <- 0

xi2 <- 0

for(i in 1:length(x)) {

xi4 <- c(xi4, ((x[i])^4))

xi3 <- c(xi3, ((x[i])^3))

xi2 <- c(xi2, ((x[i])^2))

}

sxi4 <- sum(xi4)

sxi3 <- sum(xi3)

sxi2 <- sum(xi2)

sxi <- sum(x)

xi2y <- xi2[-1] * y

sxi2y <- sum(xi2y)

xiy <- x*y

sxiy <- sum(xiy)

syi <- sum(y)

Matrix1 <- matrix(c(sxi4, sxi3, sxi2, sxi3, sxi2, sxi, sxi2, sxi, length(x)), nrow=3)

Matrix2 <- solve(Matrix1)

Matrix3 <- matrix(c(sxi2y, sxiy, syi), nrow=1)

Matrix4 <- Matrix3 %*% Matrix2

return(Matrix4)

}

QR <- QuadRegression(as.numeric(CalibData$Sample[1:6]) , CalibData$ResponseRatio[1:6]) ; QR

#Plot the Calibration Curve

png(filename="Oxymetholone_AASCalibrationCurve_13112023.png",units="in",width=6,height=5,res=300)

plot(as.numeric(CalibData$Sample[1:6]),CalibData$ResponseRatio[1:6], type = "p", pch=19, xlim=c(0,250), ylim=c(0,3),xlab="Concentration",ylab="Response Ratio")

curve(QR[1,1]*x^2 + QR[1,2]*x + QR[1,3], from = 50, to = 250, n=101, add = TRUE)

dev.off()

#Calculate the Coefficient of Determination

R2 <- function(x,y) {

pred <- 0

m <- sum(y)/length(y)

for(i in 1:length(x)) {

pred <- c(pred, (QR[1,1]*x[i]^2 + QR[1,2]*x[i] + QR[1,3]))

}

SE <- (pred[-1] - y)^2

SSE <- sum(SE)

STO <- (y - m)^2

SSTO <- sum(STO)

Rsq <- 1 - (SSE/SSTO)

return(Rsq)

}

Rsquared <- R2(as.numeric(CalibData$Sample[1:6]) , CalibData$ResponseRatio[1:6]) ; Rsquared

#Export Regression Data

RegressionData <- data.frame(QR[1],QR[2],QR[3],Rsquared) ; names(RegressionData) <- c("a","b","c","R^2") ; RegressionData

write_xlsx(RegressionData, "Oxymetholone_CalibRegressionData_13112023.xlsx")

#=========================

# QUANTITATION VALIDATION

#=========================

#Calculation of the Concentration Ratio

CalcConc <- function(x){

Conc <- 0

for(i in 1:length(x)) {

Conc <- c(Conc, (-QR[1,2] + sqrt(((QR[1,2])^2) - 4*(QR[1,1])*(QR[1,3]-x[i])))/(2*QR[1,1]))

}

return(Conc[-1])

}

CalibData[5] <- c(50, 75, 100, 150, 200, 250, CalcConc(CalibData$ResponseRatio[-(1:6)])) ; names(CalibData)[5] <- "Calculated Concentration"

# % Bias

CalibData[6] <- c(rep("NA",6),((abs(CalibData$`Calculated Concentration`[7:10]-as.numeric(CalibData$Sample[7:10])))/as.numeric(CalibData$Sample[7:10]))*100,rep("NA", NROW(as.vector(CalibData$Response[-(1:10)])))) ; names(CalibData)[6] <-"% Bias"

write_xlsx(CalibData,"/Users/CYNorman/Documents/Papers/Anabolic Steroids/Quant/Oxymetholone_SampleData_13112023.xlsx")
